# PINK1 ameliorates acute-on-chronic liver failure by inhibiting apoptosis through mTORC2/AKT signaling

**DOI:** 10.1038/s41420-022-01021-5

**Published:** 2022-04-23

**Authors:** Xuehong Yin, Ran Xue, Jing Wu, Muchen Wu, Bangxiang Xie, Qinghua Meng

**Affiliations:** 1 Department of Critical Care Medicine of Liver Disease, Beijing You-An Hospital, Capital Medical University, Beijing, China; 2 Key Laboratory of Carcinogenesis and Translational Research (Ministry of Education), Department of Gastrointestinal Oncology, Peking University Cancer Hospital & Institute, Beijing, China; 3 Beijing Institute of Hepatology, Beijing You-An Hospital, Capital Medical University, Beijing, China

**Keywords:** Liver diseases, Medical research

## Abstract

Acute-on-chronic liver failure (ACLF) is a lethal syndrome with a remarkable short-term death rate. Even worse, effective internal medicine therapies are currently lacking. Increasing evidence indicates apoptosis plays a critical role in the progression of liver failure. PINK1 has an essential function in maintaining cell survival. However, the role and underlying mechanism of PINK1 in apoptosis in ACLF are incompletely understood. Herein, our team discovered that PINK1 remarkably improved ACLF, featured by a reduction in aspartate aminotransferase (AST) and alanine aminotransferase (ALT) and an amelioration in the gross and microscopy histopathology appearance of hepatic tissues. Meanwhile, PINK1 affected cleaved caspase-3 expression via mTORC2/AKT, and this effect was eliminated after further intervention with Rictor or AKT. Overall, these findings indicate that PINK1 participates in the regulation of multiple biological functions, including hepatic cell growth and apoptosis in ACLF via the mTORC2/AKT signaling pathway. The present research offers a solid theory-wise foundation for the clinic applications of PINK1 as a valid target for ACLF treatment to reverse or postpone the development of ACLF.

## Introduction

Acute-on-chronic liver failure (ACLF) is characterized by acute decompensation of chronic liver disease, multi-organ failure, and high short-term mortality [1]. Despite progress in treatments, ACLF remains a high morbidity and mortality rate [2]. Efficient internal medicine treatments are urgently needed. While the progression and pathogenesis of ACLF are not poorly understood. Therefore, there is an urgent need to conduct in-depth research on ACLF to explore new effective treatments.

Apoptosis is a form of cell death characterized by cell shrinkage, membrane blebbing, nuclear fragmentation, and chromatin condensation [3]. Its primary role is to maintain tissue homeostasis and health by eliminating damaged and/or senescent cells and balancing cell proliferation. This function is especially critical for organs frequently exposed to toxins and viruses, especially the liver [4]. Pathological apoptosis occurs in an unregulated fashion and can be sustained and injurious [5]. Although an increasing number of studies have demonstrated that apoptosis is one of the most predominant modes of cell death in patients with ACLF [6], the pathogenesis of hepatocyte apoptosis has not been clearly illuminated.

PINK1 is a 581-amino-acid-long putative serine/threonine protein kinase [7], whose physiological roles are the regulation of mitochondrial quality, morphology, and function. PINK1-depleted mice displayed oxidation stress, aberrant mitochondria functions, and changed mitochondria shape [8]. In addition to its effect on mitochondrial function, PINK1 is also involved in regulating cell survival. Initial researches displayed that a pivotal function of the PINK1 protein was to defend cells against stress-triggered death. PINK1-depleted cells were more sensitive to programmed cell death posterior to the activation by mitochondria toxins [9]. Furthermore, overexpression of wild-type PINK1 protected cells from chemical-mediated death effects such as MPTP and MG-132 [10]. Most previous studies about PINK1 focused on neurodegenerative diseases, such as Parkinson’s disease. Recent studies suggest that PINK1 may also have a protective role in multiple liver diseases, including alcoholic liver disease, nonalcoholic steatohepatitis (NASH), and acetaminophen (APAP)-induced liver injury (AILI) [11–13]. Despite accumulating evidence has shown that PINK1 plays an essential role in cells protection, there is a lack of understanding regarding how it protects ACLF patients from apoptosis.

The mechanistic target of rapamycin (mTOR) is an evolutionarily conserved serine/threonine protein kinase. It plays a vital role in the regulation of cell growth and metabolism [14–16]. mTOR forms two complexes with different regulatory functions, mTOR complex 1 (mTORC1) and mTOR complex (mTORC2) [17]. Then, mTORC2 has the core constituents mTOR, mSIN1, mLST8, and Rictor and is remarkably non-sensitive to rapamycin. In particular, Rictor is an essential component for mTORC2 activation [18]. A study identified the PINK1-dependent activation of mTOR signaling as the major pathway activated in apoptotic cell death induced by carbonyl cyanide 3-chlorophenylhydrazone (CCCP) [19]. In contrast to mTORC1, studies on mTORC2 are very limited. More recently, mTORC2 regulates cell survival, cellular metabolism, and the cytoskeleton [20–22]. Moreover, Enhanced Akt through activation of mTORC2 provides cytoprotection. Rictor, a special constituent of mTORC2, is subjected to phosphonation via PINK1 overexpression [23–25]. However, the mechanisms underlying the regulation of the PINK1-mediated mTORC2/AKT remain elusive.

Therefore, from the clinical point of view, the related molecular mechanisms were explored through cell assays, and animal experiments were performed to confirm this hypothesis. The primary purpose of this study was to explore the expression of PINK1, mTORC2/Rictor, and p-AKT in ACLF tissue and liver cell samples. We also investigated its roles in regulating apoptosis in ACLF and the potential molecular pathways involved. Our findings provide a new direction for the treatment of ACLF.

## Results

### Apoptosis and ROS levels are significantly higher in the liver tissue of acute-on-chronic liver failure patients

**Fig. 1:**
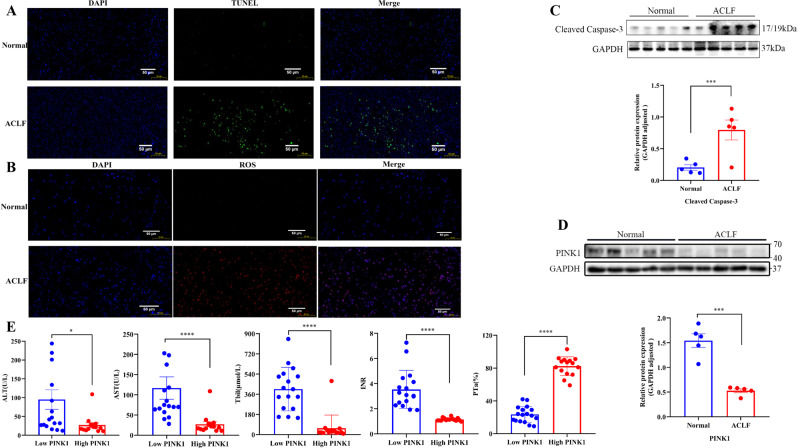
Apoptosis and ROS are induced in the liver tissue of ACLF patients. **A** TUNEL staining of liver tissue samples (green: TUNEL-positive cells; blue: DAPI; scale bar, 50 µm). **B** DHE staining of liver tissue samples (red: ROS-positive cells; blue: DAPI; scale bar, 50 µm). **C** The protein levels of cleaved caspase-3 in human liver tissue samples were detected by western blotting and analyzed with the Image J software. **D** Protein levels of PINK1 in human liver tissue samples were detected by western blotting and quantitated with the Image J software. **E** ALT (U/L), AST (U/L), Tbil (μmol/L), INR, and PTa (%) at different levels of PINK1. *P < 0.05; **P < 0.01; ***P < 0.001; ****P < 0.0001; ns not significant.

TUNEL assay and DHE staining were used to analyze the changes in the levels of apoptosis and ROS in liver tissues of ACLF patients and normal humans. ACLF liver tissues had higher TUNEL and ROS expression than normal human liver tissues (Fig. 1A, B). Western blot assay was used to determine the changes of the cleaved caspase-3 protein. The results showed the expression of the apoptotic protein cleaved caspase-3 protein was significantly upregulated in ACLF patients (Fig. 1C).

### PINK1 protein expression is significantly decreased in ACLF and correlated with liver function

Western blot analyses showed significant downregulation of PINK1 in liver samples from ACLF patients (Fig. 1D). Meanwhile, PINK1 levels were compared with liver function indicators, and patients with low PINK1 levels had significantly higher levels of ALT, AST, and total bilirubin (Tbil), and lower levels of international normalized ratio (INR) and prothrombin activity (PTa%) (Fig. 1E).

### H_2_O_2_-induced cell model

It is well known that H_2_O_2_ is a commonly used apoptosis inducer, so we first used H_2_O_2_ to establish a model in accordance with the characteristics of ACLF for studying the role of PINK1 in ACLF.

**Fig. 2:**
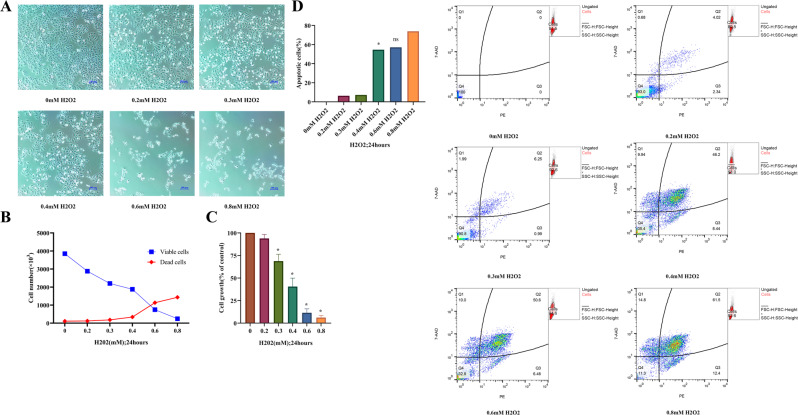
Cell model establishment. **A** Microscopic observation of the effect of different concentrations of H_2_O_2_ (0–0.8 mM) on L02 cells for 24 h (scale bar, 100 µm). **B** Effect of different concentrations of H_2_O_2_ (0–0.8 mM) on the number of L02 cells for 24 h. **C** Growth inhibition of L02 cells by H_2_O_2_ was first assessed with the CCK8 assay following treatment with different concentrations of H_2_O_2_ for 24 h. **D** Flow cytometry was performed to examine the effects of various concentrations of H_2_O_2_ (0–0.8 mM) for 24 h on L02 cell apoptosis. Annexin V-PE/7-AAD double staining and flow cytometry. *P < 0.05; **P < 0.01; ***P < 0.001; ****P < 0.0001; ns not significant.

With the increase of H_2_O_2_ concentration (0–0.8 mM, 24 h), bright-field light micrographs showed that the proliferation of L02 cells decreased significantly, and the floating cells were markedly increased. At 0.6 mM H_2_O_2_, a large number of cells were necrotic and deformed (Fig. 2A). The exposure to H_2_O_2_ (0–0.8 mM, 24 h) remarkably reduced the viable (trypan blue-negative) cellular quantity in L02 cells dosage-dependently, but H_2_O_2_ elevated the quantity of dead (trypan blue-positive) cells in a dosage-depend way (Fig. 2B, C). Based on the CCK8 assay, H_2_O_2_ (0–0.8 mM, 24 h) significantly inhibited the growth of L02 cells with an IC50 (half-maximal inhibitory concentration) of ~509 mM (95% Confidence Interval [CI] 396 to 634 mM). To evaluate the effect of apoptosis on L02 cells, flow cytometry after Annexin PE/7-AAD staining was utilized. Flow cytometry also confirmed the above apoptosis experiments. After induction with 0.4 mM H_2_O_2_ for 24 h, the apoptotic rate of L02 cells was sharply increased to 54.64%; however, after induction with 0.6 mM H_2_O_2_, the apoptotic rate of L02 cells was increased to 57.08% compared with the 0.4 mM H_2_O_2_ group (54.64%) (Fig. 2D). Then, the number of floating cells was increased significantly (Fig. 2A). So, 0.4 mM H_2_O_2_ was used as the induced condition.

### Overexpression of PINK1 confers resistance to induced cell death in L02 cells by activating AKT

**Fig. 3:**
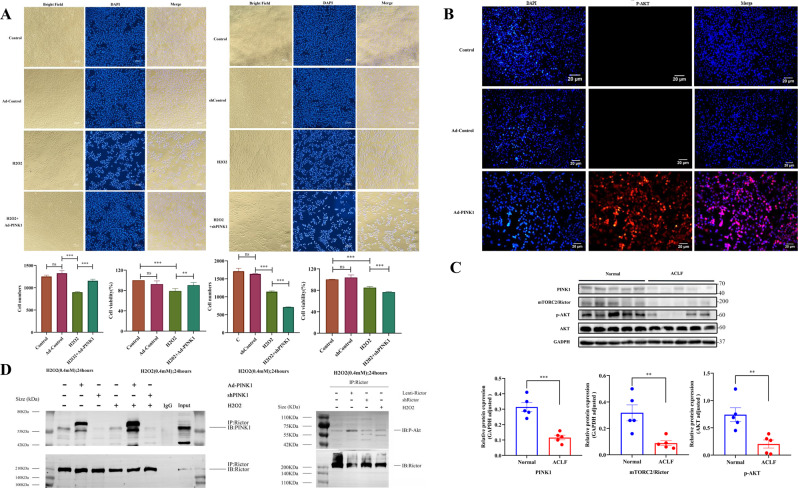
PINK1 regulates cell proliferation and enhances the phosphorylation level of Akt via activation of mTORC2. **A** L02 cells were transfected with PINK1 overexpression or PINK1 shRNA. The CCK8 assay was conducted to examine the effects of Ad-PINK1 and shPINK1 on the proliferation of H_2_O_2_-induced cells (0.4 mM, 24 h). Cells were counted, and cell morphology was observed under a light microscope (scale bar, 100 µm). **B** Immunostaining for Ser-473-phosphorylated Akt detection in L02 cells infected with indicated constructs in adenovirus vectors (Ad-PINK1) (scale bar, 20 µm). **C** Western blot analysis was performed to detect the protein expression levels of PINK1, mTORC2/Rictor, and p-Akt (S473) in human liver tissue samples and analyzed with the Image J software. **D** L02 cells cultured with Ad-PINK1 for 72 h were coimmunoprecipitation. PINK1 interacts with Rictor in L02 cells and the H_2_O_2_-induced cell model. Cell lysates were subjected to coimmunoprecipitation with anti-PINK1 antibody or control rabbit IgG, followed by western blot analysis, phosphorylation of Akt by coimmunoprecipitation(Co-IP) in vitro. Immunoprecipitates with designated antibodies were used for the kinase assay using recombinant Akt protein as a substrate in vitro. Rictor is known to be a component of mTORC2. Cell lysates were subjected to immunoprecipitation with anti-Rictor antibody or control rabbit IgG. *P < 0.05; **P < 0.01; ***P < 0.001; ****P < 0.0001; ns not significant.

First, we examined the effects of cell survival by overexpression and knockdown of PINK1, respectively. The CCK8 assay revealed that PINK1 overexpression promoted the proliferation of L02 cells after H_2_O_2_ injury (0.4 mM, 24 h). In contrast, cell proliferation was significantly inhibited by the knockdown of PINK1 (Fig. 3A). To explore the downstream targets of PINK1, we examined the change of AKT phosphorylation levels. As shown in Fig. 3B, PINK1 overexpression significantly enhanced the phosphorylation of Akt at Ser-473. The result was confirmed by immunostaining for phosphorylated Akt detection.

### PINK1-mediated AKT phosphorylation via mTORC2/Rictor in apoptosis

Some studies suggested that the mTOR signaling pathway may be regulated by PINK1 [23]. The mTORC2 complex is composed of mSIN1, mLST8, and mTOR, while Rictor is its core component [26]. We found that the changes of PINK1, Rictor, and p-AKT in the liver tissue of ACLF patients were consistent. Namely, Rictor and p-AKT were decreased significantly in the liver tissue of ACLF patients (Fig. 3C). A coimmunoprecipitation (Co-IP) was performed, and PINK1 was found to coimmunoprecipitate with Rictor. Furthermore, immunoprecipitates with an antibody against rictor phosphorylated Akt at Ser-473 in vitro (Fig. 3D). This confirms the interaction between PINK1, mTORC2, and AKT in vitro.

**Fig. 4:**
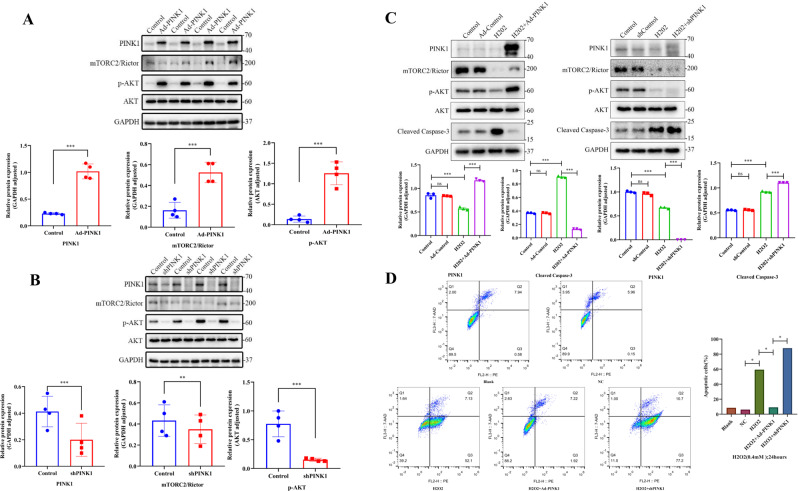
Mechanism of PINK1-mediated caspase-3-mediated apoptosis. **A** Changes in mTORC2/Rictor and p-AKT expression levels, detected by western blot analysis in PINK1-overexpressed cells for 72 h. **B** L02 cells were transduced with PINK1 shRNA; the protein levels of mTORC2/Rictor and p-AKT were examined and analyzed by Image J. **C** Effect of PINK1 overexpression or knockdown on caspase-3-mediated apoptosis in the H_2_O_2_-induced cell model, detected by western blotting and analyzed with the Image J software. **D** Flow cytometry further confirmed these results. *P < 0.05; **P < 0.01; ***P < 0.001; ****P < 0.0001; ns not significant.

We further demonstrated that PINK1 mediated AKT phosphorylation via mTORC2/Rictor in apoptosis. After overexpression of PINK1 in L02 cells, the expression level of PINK1 was markedly increased, and the expression levels of mTORC2/Rictor and p-AKT were increased significantly in the PINK1-overexpression group (Fig. 4A). After PINK1 downregulation using siRNA, WB also demonstrated that the expression levels of mTORC2/Rictor and p-AKT proteins were obviously reduced (Fig. 4B).

Then, we tested the apoptosis-related mechanism mediated by PINK1 in the H_2_O_2_-induced cell model. Likewise, mTORC2/Rictor and p-AKT amounts increased significantly after PINK1 overexpression in the H_2_O_2_-induced cell model, while cleaved caspase-3 protein expression was significantly decreased. After PINK1 silencing, mTORC2/Rictor and p-AKT expression levels decreased significantly. Meanwhile, cleaved caspase-3 protein expression was significantly elevated (Fig. 4C). Flow cytometry further confirmed that PINK1 overexpression decreased apoptosis, while its deficiency enhanced apoptosis (Fig. 4D).

**Fig. 5:**
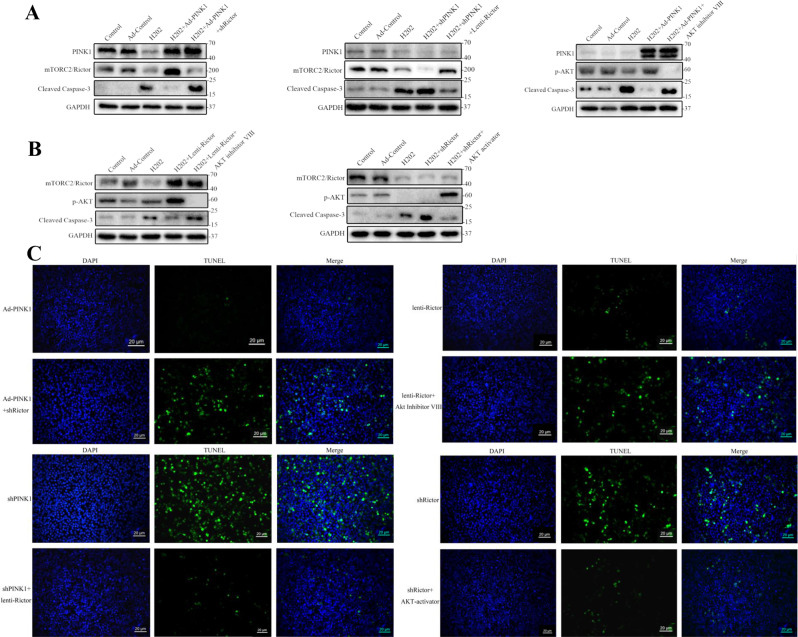
The relationship between the PINK1/mTORC2/p-AKT pathway and apoptosis. **A** In the H_2_O_2_-induced cell model, overexpression of PINK1 followed by knockdown of Rictor or inhibition of AKT activity with AKT inhibitors VIII (10 µM), and knockdown of PINK1 with subsequent overexpression of Rictor, we further detected the expression of the apoptosis-related protein cleaved caspase-3 by WB. **B** Overexpression of Rictor followed by inhibition of AKT activity with AKT inhibitors VIII, and further activation of AKT after knockdown of Rictor, with cleaved caspase-3 detection by western blotting. **C** TUNEL assay performed in the same experimental conditions as above (green: TUNEL-positive cells; blue: DAPI; scale bar, 20 µm).

To further demonstrate the relationship between the PINK1/mTORC2/p-AKT pathway and apoptosis, we found that cleaved caspase-3 was highly expressed when Rictor was knocked down after PINK1 overexpression. Overexpression of Rictor after knockdown of PINK1 resulted in a significant decrease in the apoptosis-related protein cleaved caspase-3. Comparing the changes of apoptotic proteins after overexpression of PINK1 in the context of AKT inhibition, the expression of cleaved caspase-3 was increased (Fig. 5A). Meanwhile, treatment with Rictor overexpression (Lenti-Rictor) in L02 cells increased Rictor protein levels significantly, which was also accompanied by a marked rise in the expression of p-Akt (Ser-473). Similarly, knockdown Rictor (shRictor) markedly decreased protein expression of rictor and inhibited the phosphorylation of Akt at Ser-473. The expression of cleaved caspase-3 in the Rictor overexpression and AKT inhibitor group was significantly higher than that of the Rictor-overexpression group alone. In addition, knockdown of Rictor followed by reactivation of AKT showed a decrease in cleaved caspase-3 (Fig. 5B). Meanwhile, these results were confirmed by the TUNEL assay (Fig. 5C). The above results confirm that the regulation of apoptosis by PINK1 can be influenced by Ritor/AKT.

### PINK1 inhibited apoptosis through the activation of the mTORC2/p-AKT pathway in an ACLF mouse model

**Fig. 6:**
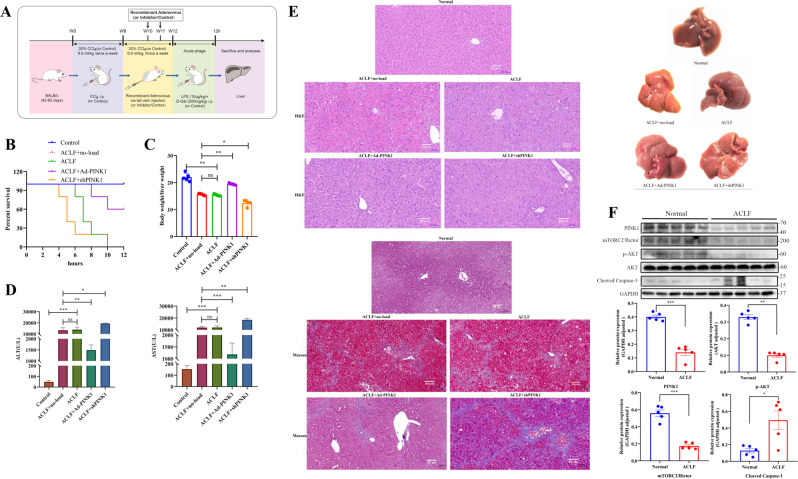
In vivo experiments confirmed the results of the above in vitro findings. **A** Schematic representation of mouse model establishment and lentivirus injection. **B** Survival rates in the normal, ACLF + no-load, ACLF, ACLF + Ad-PINK1, and ACLF + shPINK1 mouse groups. **C** Body-to-liver weight ratio in the control group. **D** Serum ALT and AST levels. **E** Morphological comparison of liver samples among the normal, ACLF + no-load, ACLF, ACLF + Ad-PINK1, and ACLF + shPINK1 groups (scale bar, 50 µm). **F** WB experiments were performed to evaluate changes in PINK1, mTORC2, p-AKT, and cleaved caspase-3 expression. *P < 0.05; **P < 0.01; ***P < 0.001; ****P < 0.0001; ns not significant.

For the sake of establishing a mice model of ACLF, our team completed the following combined therapies: 8-week persistent CCl_4_ (20%, 6.6 ml/kg, two times every 7 days), an acute injection of a 1.5-fold dosage of CCl_4_ (20%, 6.6 ml/kg, three times every 7 days) for 28 days and LPS (10 µg/kg)+d-Gal (500 mg/kg) (Fig. 6A).

The mouse model had the closest features to ACLF, showing high short-term mortality within 12 h (100%). The survival times and body-to-liver weight ratios of ACLF mice were improved after PINK1 overexpression. Knockdown of PINK1 further decreased survival time (Fig. 6B, C). Meanwhile, serum ALT and AST levels were significantly higher in the ACLF + shPINK1 group compared with the ACLF + no-load group (*P* < 0.05). Serum ALT and AST levels were lowest in the ACLF + Ad-PINK1 group (Fig. 6D). Importantly, HE and Masson staining of the liver biopsy confirmed an ACLF. Furthermore, severe pathological liver damage, including necrosis, was more pronounced in the PINK1 knockdown group, and this effect was significantly attenuated following PINK1 overexpression (Fig. 6E).

We further examined the expression levels of PINK1, mTORC2, p-AKT, and cleaved caspase-3 in the liver tissue in the mouse model of ACLF by western blotting. Significantly lower PINK1, mTORC2/Rictor, and p-AKT amounts were observed in the model group, while cleaved caspase-3 was upregulated (Fig. 6F). These results are consistent with those of human ACLF, indicating the success of mouse model establishment.

**Fig. 7:**
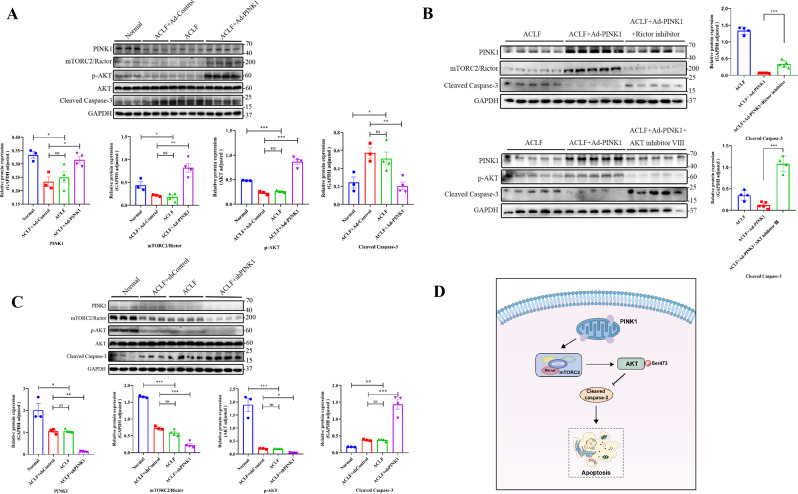
Study of apoptosis after overexpression and/or knockdown of related factors in animal models. **A** Overexpression of PINK1 in the ACLF mouse model, and detection of changes in apoptotic proteins. **B** Based on PINK1 overexpression, Rictor and AKT inhibitors were supplemented, respectively, and cleaved caspase-3 changes were assessed. **C** Further knockdown of PINK1 in the ACLF model was performed to observe the alteration of the apoptosis-related protein cleaved caspase-3 by WB; Image J was used for analysis. **D** The theoretical model demonstrating how PINK1 inhibits apoptosis through the activation of the mTORC2/p-AKT pathway. *P < 0.05; **P < 0.01; ***P < 0.001; ****P < 0.0001; ns not significant.

Finally, adenoviruses expressing Ad-PINK1 or shPINK1 were intravenously injected into BALB/c mice through the tail vein. Cleaved caspase-3 protein levels were significantly decreased for the PINK1-overexpression group (Fig. 7A). On the basis of PINK1 overexpression, we further used an inhibitor of Rictor or AKT and examined cleaved caspase-3. Surprisingly, western blot analysis revealed increased activated cleaved caspase-3 (Fig. 7B). After PINK1 downregulation, cleaved caspase-3 was significantly upregulated (Fig. 7C). These results further indicated that PINK1 inhibited apoptosis through the activation of the mTORC2/p-AKT pathway (Fig. 7D).

## Discussion

Previous studies have demonstrated that PINK1 exerts its hepatoprotective effect by regulating multiple molecular pathways, including apoptosis [11, 27, 28]. However, the involvement and regulation of PINK1 in the pathogenesis of liver diseases remain poorly understood, especially in ACLF. Herein, our team displayed the protection role of PINK1 in hepatocytes in ACLF via both in vitro and in vivo assays. We also showed that apoptosis was involved in the PINK1-mediated hepatoprotective effect in ACLF. We further demonstrated that the protective mechanisms of PINK1-mediated apoptosis were mainly mediated by the mTORC2/Rictor-AKT pathway. Functionally, PINK1 deficiency resulted in increased hepatocyte apoptosis and aggravated ACLF, supporting a protective role for PINK1-mediated apoptosis. This suggested that PINK1 exerted antiapoptotic effects in ACLF. At the same time, PINK1 remarkably decreased the degree of ACLF injury and facilitated the survival of hepatic cells. Therefore, PINK1 may be a potential therapeutic target for patients suffering from ACLF, given its role in antiapoptotic processes.

It is well known that apoptosis has been one of the major pathways that leads to the process of cell death. The appropriate control of programmed cell death is pivotal for healthy tissular development, homoiostasis, and the eradication of impaired or aberrant cells. The programmed cell death of liver cells is vital in liver pathological studies and is pivotal for the majority of hepatic impairment. Despite the fact that the programmed cell death of hepatocytes has been considered a vital pathologic causal link of ACLF [29–31], the causal links of the programmed cell death of hepatic cells and their treatment efficacy still need exploration. Multiple preclinical models and clinical trials have demonstrated that inhibition of hepatocyte apoptosis can reverse or retard liver disease progression. In light of these discoveries, caspase inhibitors may be a potential therapeutic agent for treating liver disease. Other studies in the field have confirmed that serum ALT levels in patients with NASH were significantly decreased after treatment with the caspase inhibitor GS-9450 [32]. Cold ischemia/warm reperfusion (CI/WR)-induced apoptotic injury during liver transplantation was significantly ameliorated when IDN-6556 was administered in cold storage and flush solutions [33]. In this study, apoptosis was found in ACLF. In vitro, PINK1 overexpression was shown to promote cell survival by inhibiting apoptosis. Moreover, loss of PINK1 resulted in apoptotic cell death. In ACLF mice, upon overexpression of PINK1, liver function was significantly improved, while liver damage and apoptosis were significantly decreased. Therefore, PINK1 as a rising star in ACLF triggers apoptosis to alleviate liver injury.

Our team discovered that PINK1 reinforced the phosphonation degree of Akt via stimulating mTORC2 posterior to cell-related and animal assays. mTOR plays a pivotal role in cells, specifically regulating cell survival. It’s known to all that mTORC2 possesses unique components like Rictor. It is still unclear about the upstream of mTORC2. Some researches have revealed that PINK1 was the most promising choice of proximate upstream modulators of mTORC2 [23, 26, 34, 35]. Further, it has been shown that the phosphorylation of Akt at Ser-473 was greatly reduced, and the activity of Akt was significantly decreased in mTORC2-deficient cells [36–39]. Our results showed that PINK1 overexpression affected the phosphorylation levels of Akt at Ser-473, in accordance with the above observations. In the meantime, the Co-IP experiment showed that PINK1 interacts with Rictor. Furthermore, Rictor expression was increased following PINK1 overexpression, whereas its expression was repressed after PINK1 knockdown. It is exciting that apoptosis was downregulated with PINK1 overexpression. Then, apoptosis was again upregulated by knocking down Rictor on the basis of PINK1 overexpression. Cleaved caspase-3 was upregulated after PINK1 knockdown, and Rictor overexpression rescued PINK1 knockdown-induced apoptosis. In vivo experiments also validated the results mentioned above. This suggests that PINK1 inhibits apoptosis through AKT activation via mTORC2 to protect from acute-on-chronic liver failure.

This study had some limitations. It is known that PINK1 is also associated with mitochondrial autophagy and that mitochondrial autophagy is involved in hepatic failure protection, so the protective mechanism of PINK1 against hepatic failure may not only be its mediated apoptotic process, but more mechanisms still need to be further explored. Meanwhile, further exploration is necessary to determine the appropriate target of PINK1 in clinical practice for ACLF.

In summary, hepatocyte apoptosis has been recognized as a prominent driver of liver disease pathogenesis. Nevertheless, the all-round details of the apoptotic signal transmission net warrant more exploration. Apoptosis/anti-apoptosis regulatory targets and their clinical applications may require special attention within this field of study. The research on hepatocyte apoptosis can contribute to developing new approaches for treating liver diseases, especially for ACLF. In this background, our discovery of a novel pathway involving PINK1 offers an essential path to explore new possibilities that may lead to effective strategies for new treatment interventions for patients suffering from ACLF.

## Materials and methods

### Cells, chemicals, and antibodies

The hepatocyte L02 cell line (Beijing Institute of Hepatology, Beijing You-An Hospital, Capital Medical University) was cultured in DMEM (Gibco, USA) supplemented with 10% fetal bovine serum (Gibco, USA). For chemicals and antibodies, see supplemental Materials and Methods.

### Patients, liver samples

Following ethical and institutional guidelines and after informed consent from the tissue donors, human liver samples were collected from surgical resections in Beijing You-An Hospital (Beijing, China). Normal liver tissue was obtained from donor’s livers rejected for clinical transplantation. Detailed clinical and pathological features are listed in Supplementary Table S1. Human sample collection was approved by the Ethics Committee of Beijing You-An Hospital, Capital Medical University of Science and Technology (Ethics approval number: Jing-you-ke-lun-zi[2019]012-hao) and conformed to the Declaration of Helsinki. Importantly, inclusion and exclusion criteria were: ACLF diagnosis by pathological analysis; meeting the diagnostic criteria for ACLF.

### In vitro validation: cell experiments

Adenovirus overexpression of PINK1 (Ad-PINK1), siRNA specific for PINK1 (shPINK1), and no-load control viruses were designed and synthesized by Shanghai Ji Kai Gene Technology Co. Ltd. Lentiviral vectors for Rictor overexpression (Lenti-Rictor) and Rictor-knockdown (shRictor) (GeneChem, China) were used for corresponding cells by lentivirus-mediated transfection. Posterior to the 36-h transfectional process, cells were exposed to 0.5 μg/mL puromycin (Medchemexpress, HY-B1743A) to acquire stable cell lines. To examine the effect of PINK1 overexpression (Ad-PINK1) or downregulation (shPINK1) on mTORC2 and p-AKT expression, treatment regimens were administered as follows: Control group, L02 cells incubated at 37 °C for 72 h; Ad-PINK1 and shPINK1 groups, L02 cells were incubated with the indicated virus (Ad-PINK1 and shPINK1) at the multiplicity of infection (MOI) of 100 at 37 °C for 72 h. Further validation was performed in H_2_O_2_-induced cell models. The treated cells were subjected to the following experiments: Control, no*-*load group, H_2_O_2_-induced model, H_2_O_2_ + Ad-PINK1 or H_2_O_2_ + shPINK1, H202 + Ad-PINK1 + shRictor, H_2_O_2_ + Ad-PINK1 + AKT inhibitor VIII [40], H_2_O_2_ + shRictor+AKT activator [41], and so on. For further detailed experimental procedures, please see Appendix Supplemental Materials and Methods.

### Animal model of ACLF

Healthy male BALB/c mice (42–62 days) were housed under standard conditions. Mice were acclimated to laboratory conditions for one week before experimentation. PINK1 overexpression, PINK1 knockdown, and control adenoviruses were designed and synthesized by Shanghai Ji Kai Gene Technology Co. Ltd. The specific treatment and groups were listed in the Supplemental Materials and Methods. Mice were sacrificed 12 h after the final treatment, and liver tissue and serum samples were collected. Hematoxylin and eosin (H&E) staining and Masson staining were performed to confirm the successful construction of the ACLF mouse model.

The animal experiments were ethically approved and supervised by the Committee on the Ethics of Animal Experiments of Capital Medical University (Ethics Approval Number: AEEI-2020-195).

### CCK8 assay

The cell counting kit-8 (CCK8) assay was used to measure cell viability and growth. For detailed experimental procedures, see Supplemental Materials and Methods.

### TUNEL assay

Cell apoptosis was analyzed using a one-step TUNEL cell apoptosis detection kit (KeyGEN, Nanjing, China). TUNEL staining was described in Supplemental Materials and Methods.

### Western blot and coimmunoprecipitation (Co-IP) analyses

Western blot (WB) analysis was carried out as described previously [42].

Coimmunoprecipitation was performed to determine protein–protein interactions between PINK1 and Rictor or p-AKT.

For detailed experimental procedures, see Supplementary Methods.

For the original image of western blots, see Supplemental Material.

### Annexin V staining and flow cytometry

According to the manufacturer’s instructions, cells were stained with the Annexin V-PE Apoptosis Detection Kit (#559763, BD, USA). Flow cytometry to analyze the percentage of apoptotic cells was performed on a BD FACSCalibur flow cytometer (BD Biosciences). Data were analyzed with the FlowJo software (Treestar, Ashland, OR, USA).

### Immunofluorescence (IF)

Immunofluorescence analysis was performed as follows. Sterile coverslips were placed into 12-well plates. Cells were inoculated at 5 × 10^5^ per well. Cells were treated as indicated prior to fixation in 4% paraformaldehyde and then immersed in 0.1% Triton X-100 for 10 min. The cells were cultured on coverslips, incubated with antibodies specific for p-AKT (CST, USA) at 4 °C overnight, and treated with fluorescent secondary antibody (1:100 dilution) for two hours at room temperature in the dark. Nuclei were stained using the dye DAPI. The fluorescence microscope (Nikon Eclipse Ti-S, Nikon Instruments Inc., USA) was used for imaging.

### Liver function assays

Serum ALT and AST contents were automatically identified via an AU400 biochemistry analyzing apparatus.

### Hematoxylin–eosin (H&E) staining

Liver samples were fixed with 10% formaldehyde, dehydrated with different concentrations of ethanol, sectioned. Paraffin sections were washed with distilled water, stained with hematoxylin and eosin, then sequentially dehydrated, transparent, sealed, finally observed by microscope. Routine H&E dyeing was completed as aforementioned [43]. The dyeing outcomes were studied by two pathological experts in a blind manner.

### Statistical analysis

Statistic assay was completed via SPSS 26.0 (IBM, America) and GraphPad Prism 8.0 (America). Data were displayed as average ± s.d. (Standard deviation). Groups were compared by two-tailed Student’s *t* test, ANOVA, or χ^2^ test. Survival analysis was conducted with the Kaplan–Meier survival method. Differences were statistically significant at *P* < 0.05.

## Supplementary information

original western blots

original western blots

original western blots

original western blots

original western blots

original western blots

original western blots

original western blots

original western blots

original western blots

original western blots

original western blots

original western blots

original western blots

original western blots

original western blots

original western blots

original western blots

original western blots

original western blots

original western blots

original western blots

original western blots

original western blots

original western blots

original western blots

original western blots

original western blots

original western blots

original western blots

original western blots

original western blots

original western blots

original western blots

original western blots

original western blots

original western blots

original western blots

original western blots

original western blots

original western blots

original western blots

original western blots

original western blots

original western blots

original western blots

original western blots

original western blots

original western blots

original western blots

original western blots

original western blots

original western blots

original western blots

original western blots

original western blots

original western blots

original western blots

original western blots

original western blots

original western blots

original western blots

original western blots

original western blots

original western blots

original western blots

original western blots

original western blots

original western blots

original western blots

original western blots

original western blots

original western blots

original western blots

original western blots

original western blots

original western blots

original western blots

original western blots

original western blots

original western blots

original western blots

Supplementary Table S1

Supplemental Materials and Methods

Supplemental Result

SUPPLEMENTAL LEGENDS

Supplemental figure 1

Supplemental figure S1

Supplemental figure S2

Supplemental figure S3

## Data Availability

The datasets used and/or analyzed during this study are available from the corresponding author on reasonable request.
