# Supplementary figures and images for: PINK1 ameliorates acute-on-chronic liver failure by inhibiting apoptosis through mTORC2/AKT signaling
Source: Cell Death Discov. 2022 Apr 23;8:222. doi: 10.1038/s41420-022-01021-5 (PMC9035184; doi:10.1038/s41420-022-01021-5)

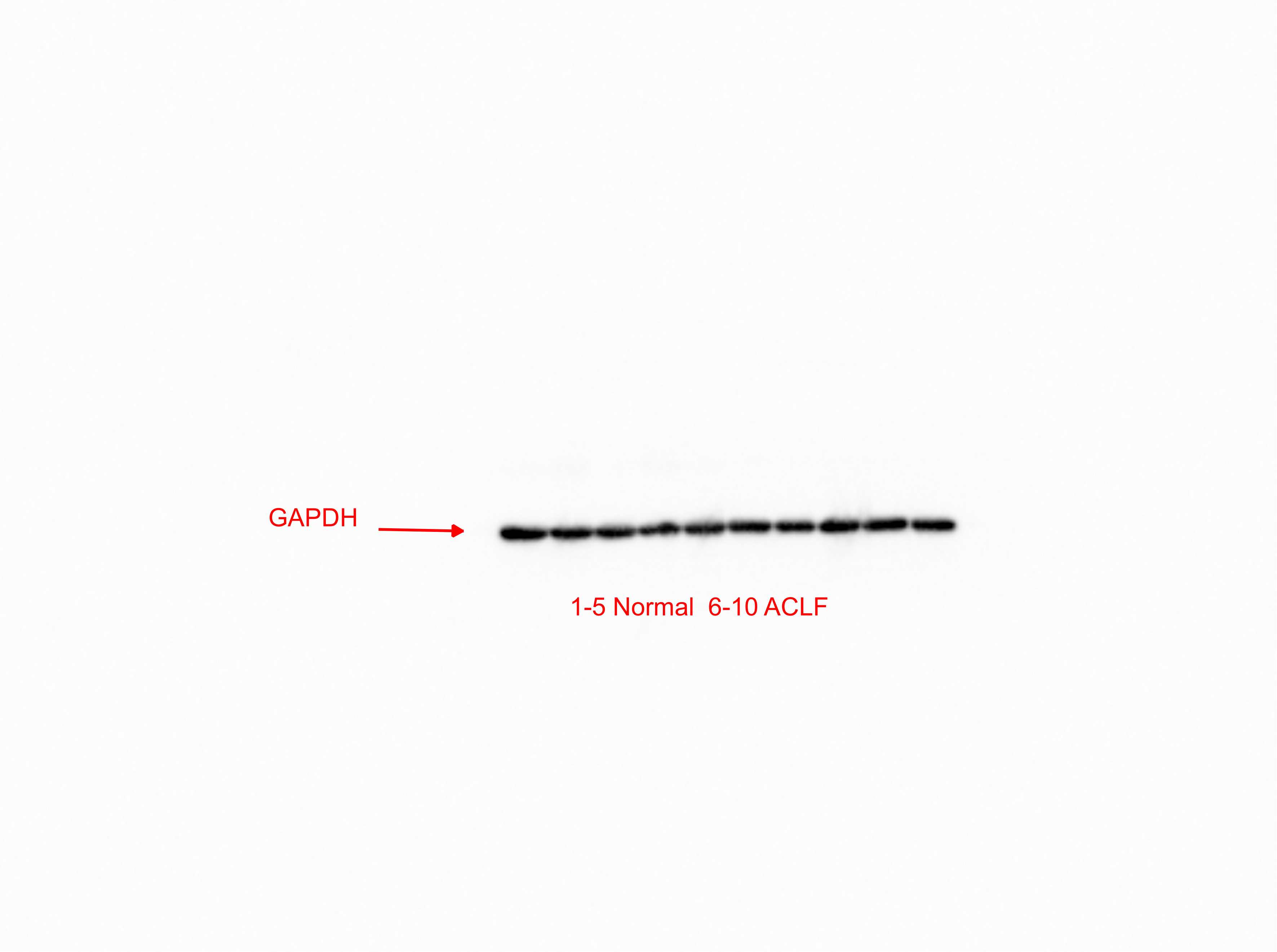

Supplement: Supplementary file 1 — original western blots [file 41420_2022_1021_MOESM1_ESM.jpg]

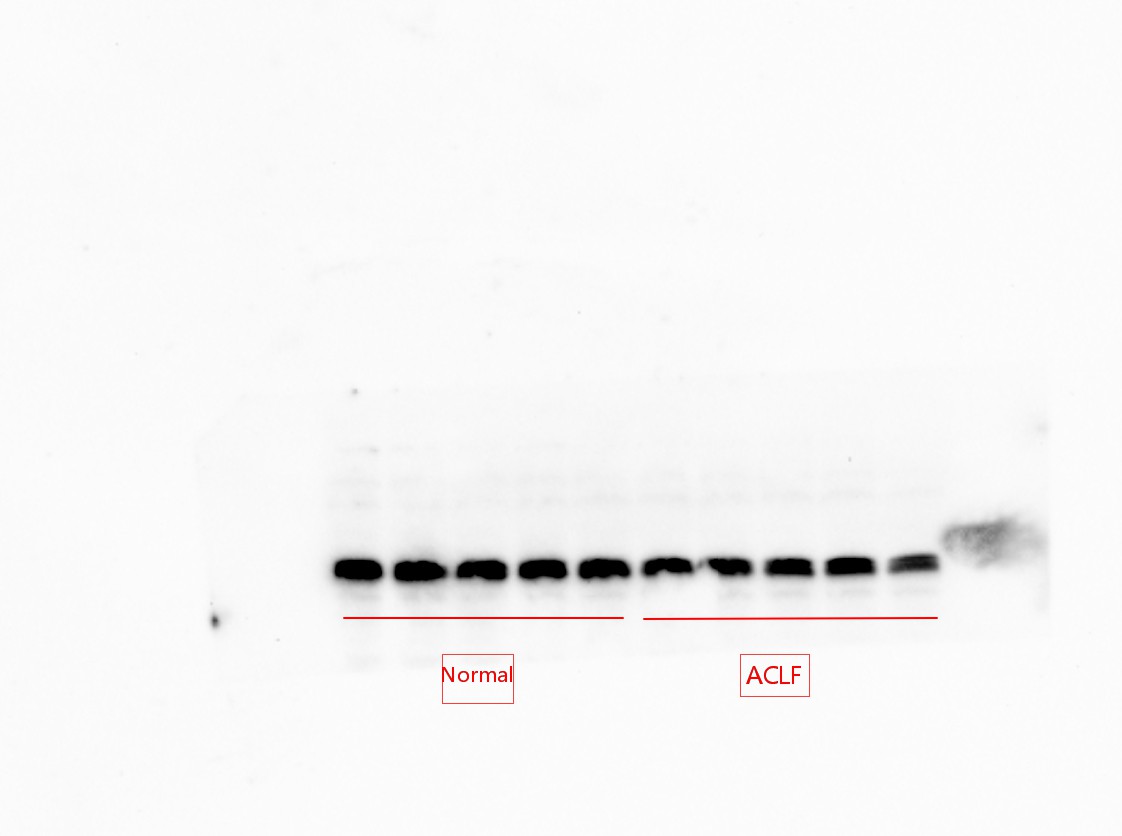

Supplement: Supplementary file 2 — original western blots [file 41420_2022_1021_MOESM2_ESM.jpg]

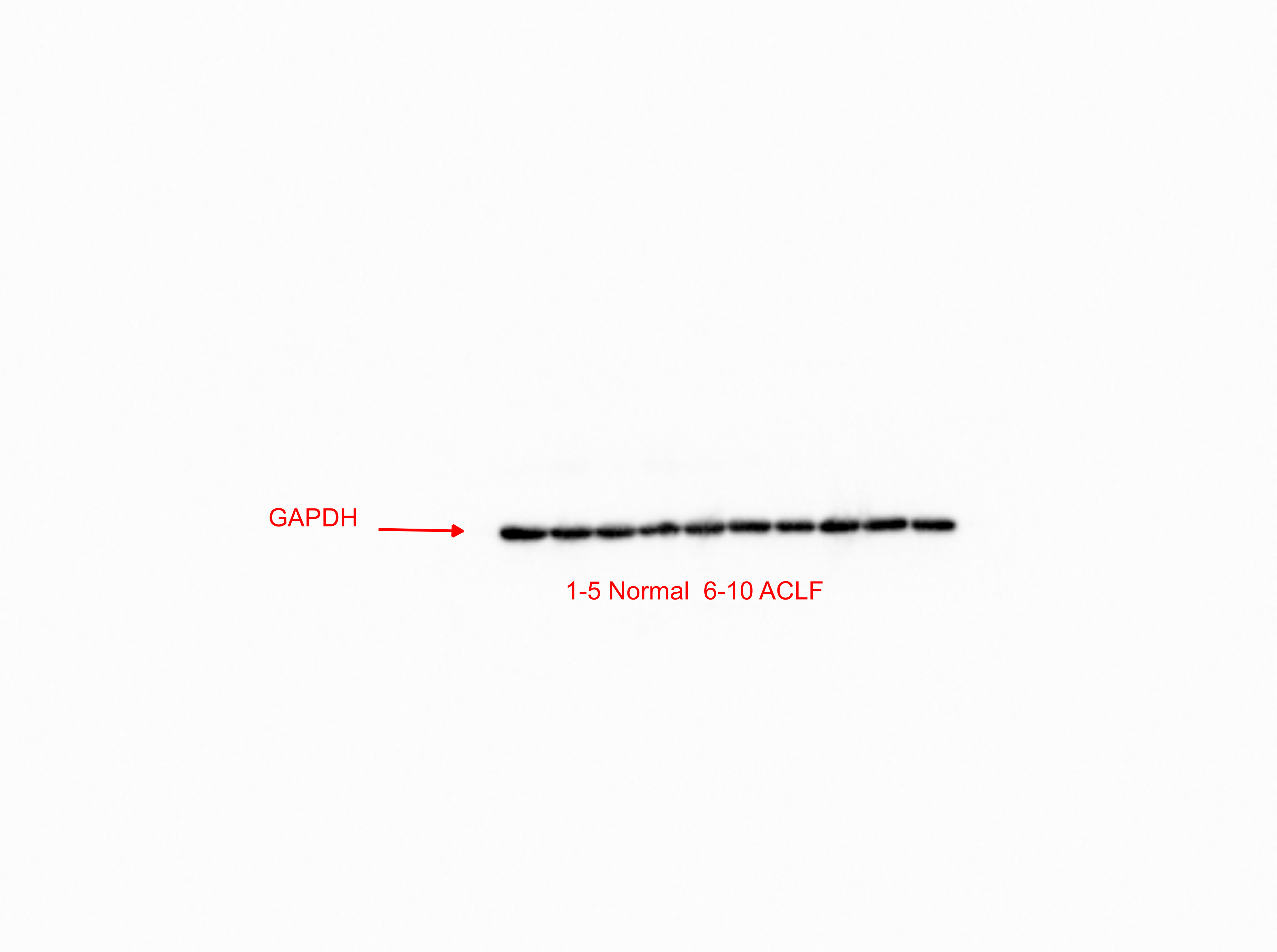

Supplement: Supplementary file 3 — original western blots [file 41420_2022_1021_MOESM3_ESM.jpg]

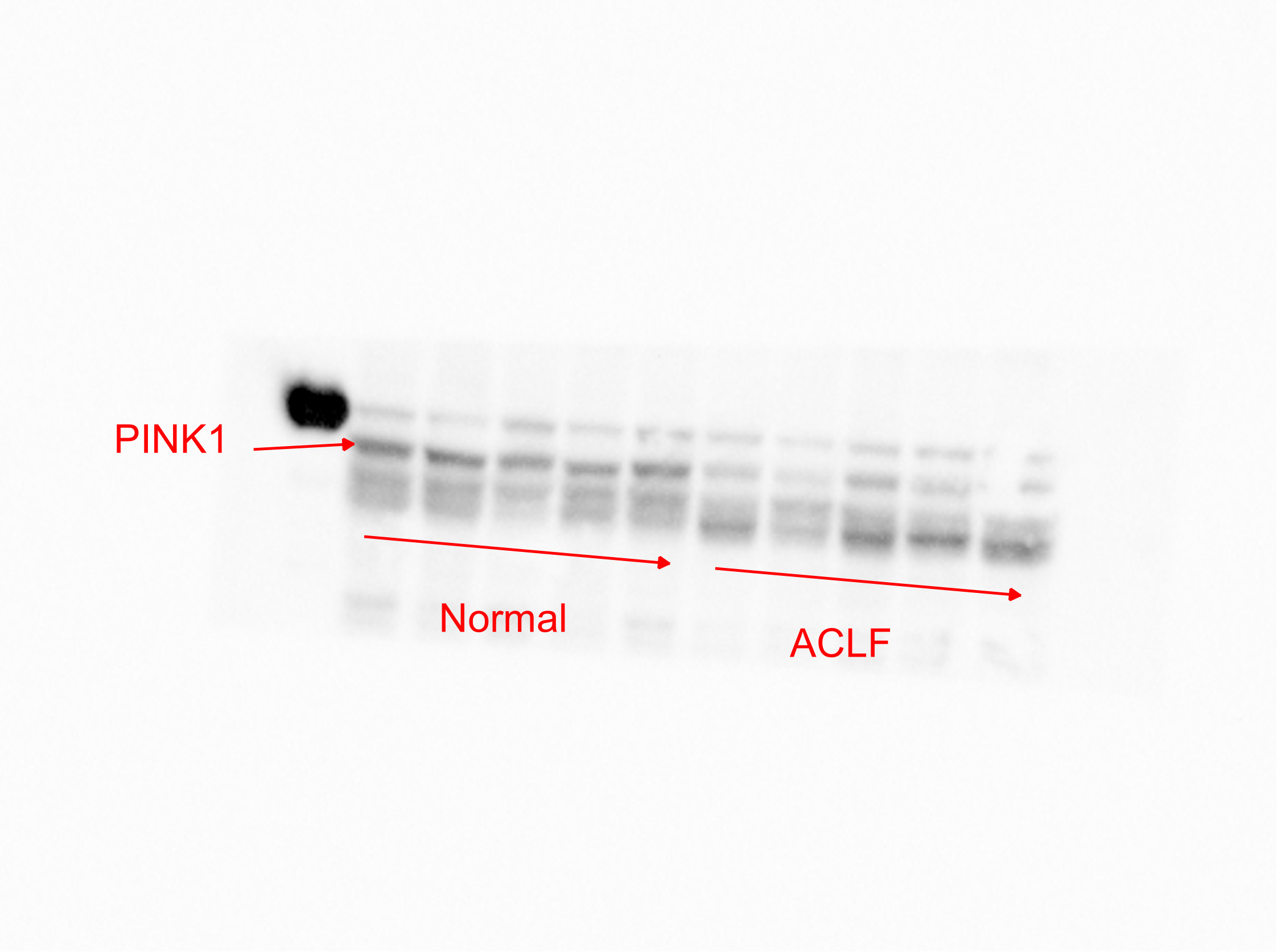

Supplement: Supplementary file 4 — original western blots [file 41420_2022_1021_MOESM4_ESM.jpg]

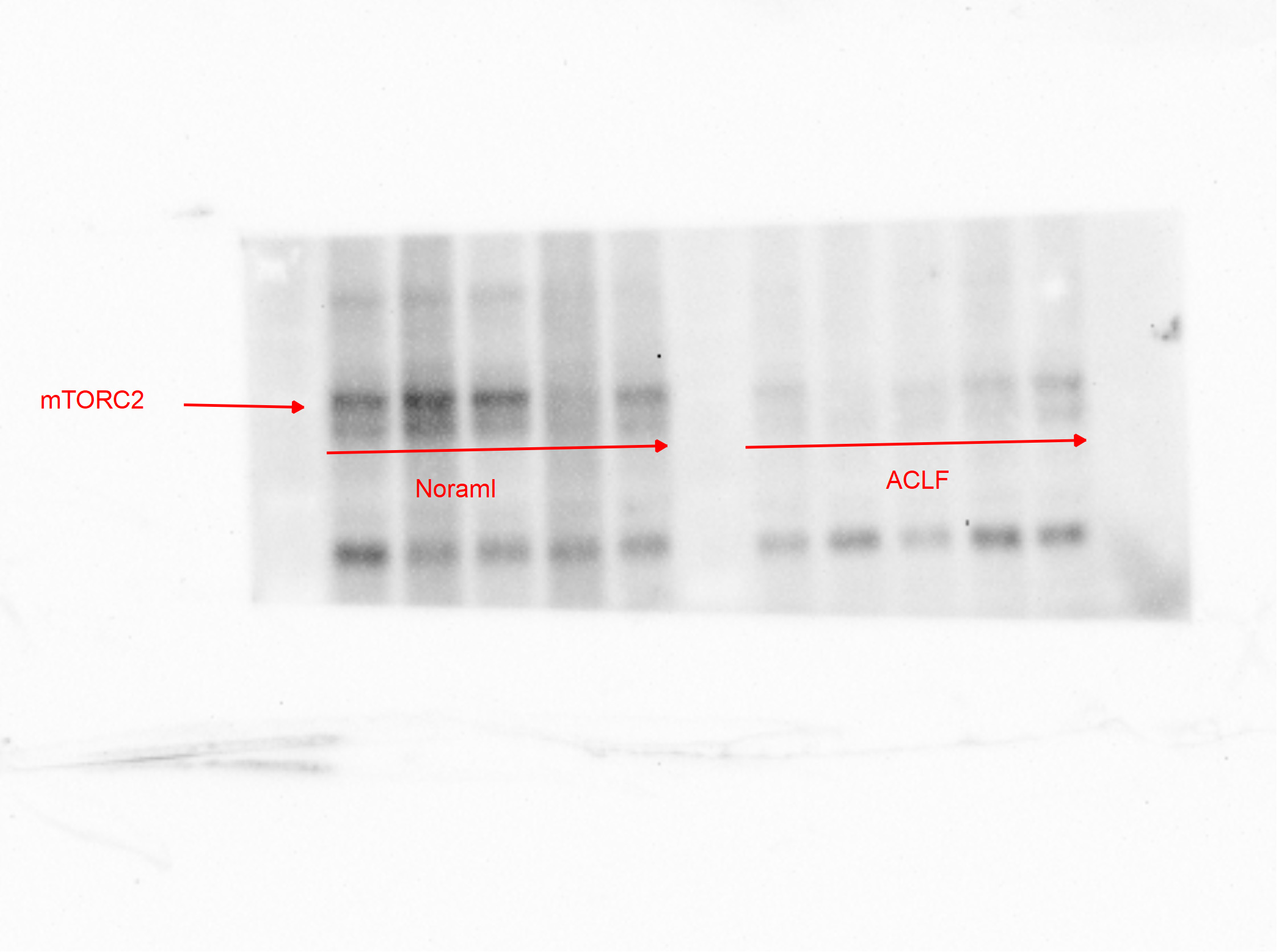

Supplement: Supplementary file 5 — original western blots [file 41420_2022_1021_MOESM5_ESM.jpg]

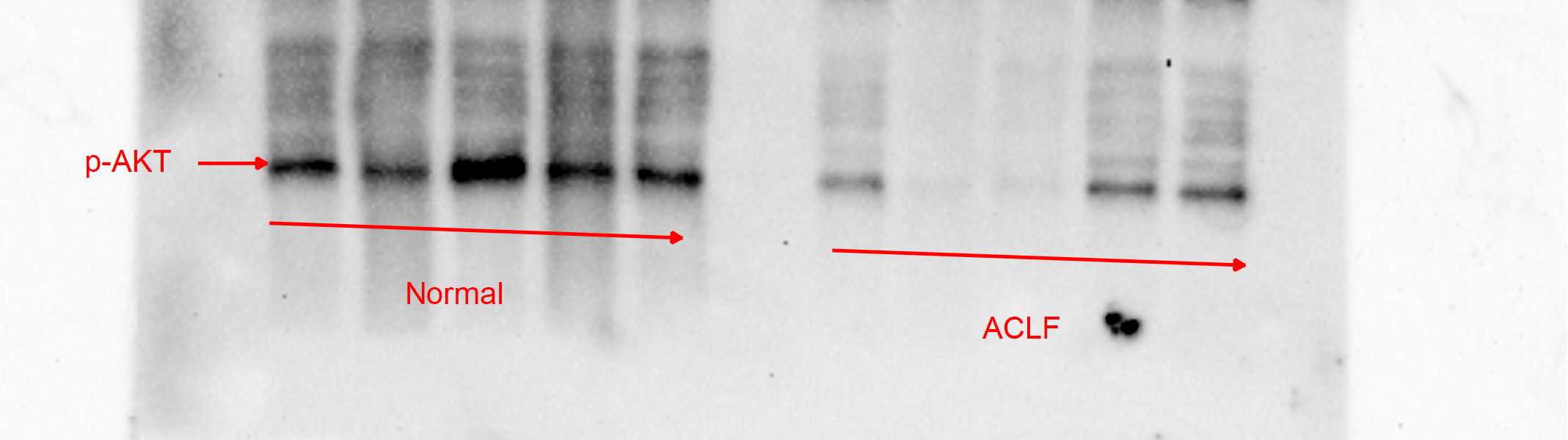

Supplement: Supplementary file 6 — original western blots [file 41420_2022_1021_MOESM6_ESM.jpg]

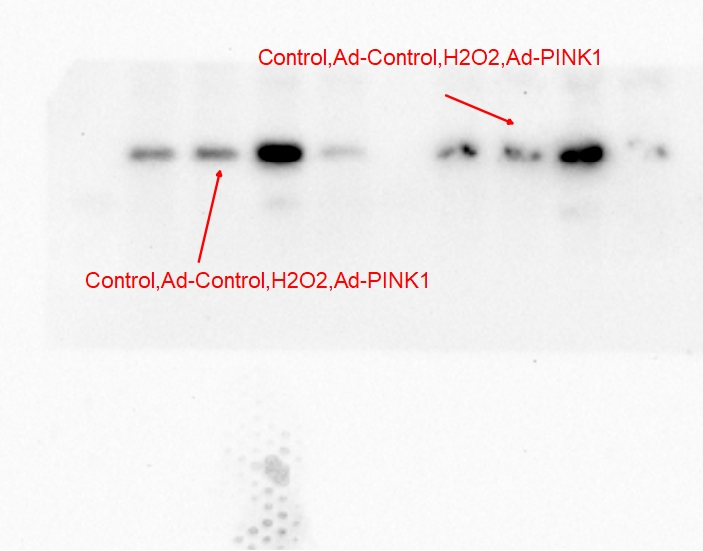

Supplement: Supplementary file 7 — original western blots [file 41420_2022_1021_MOESM7_ESM.jpg]

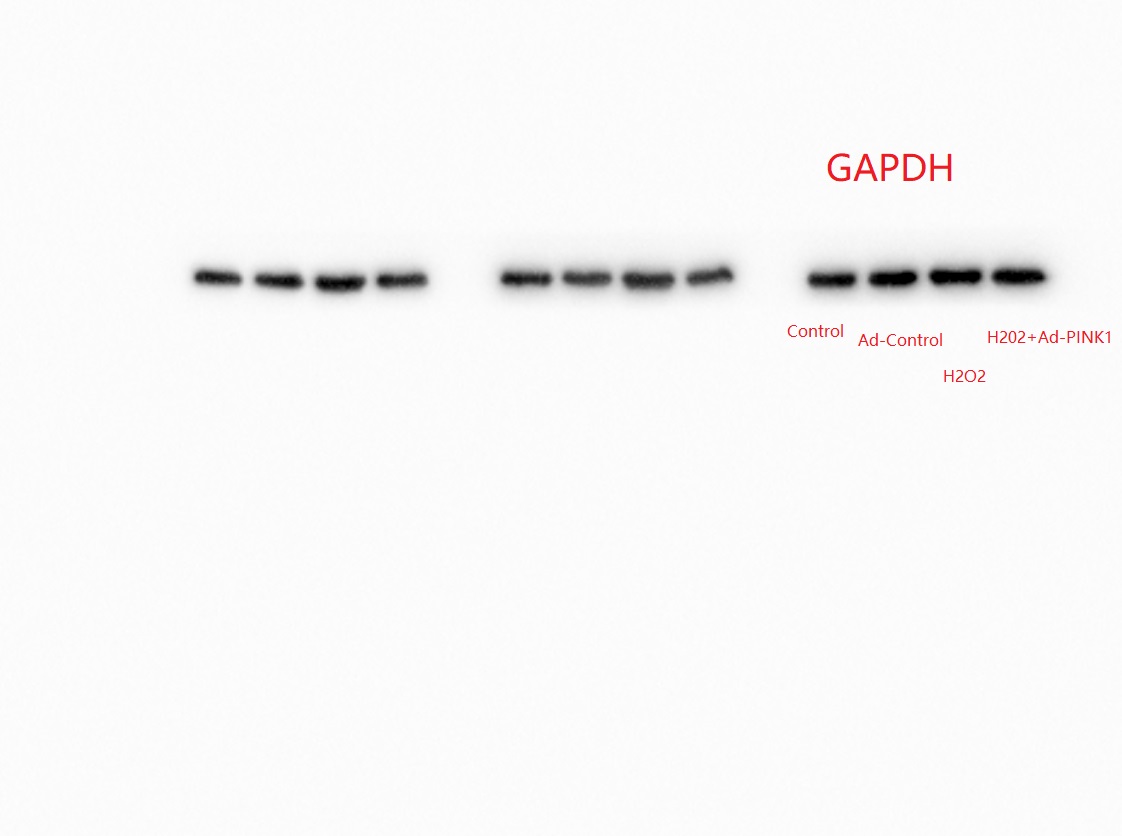

Supplement: Supplementary file 8 — original western blots [file 41420_2022_1021_MOESM8_ESM.jpg]

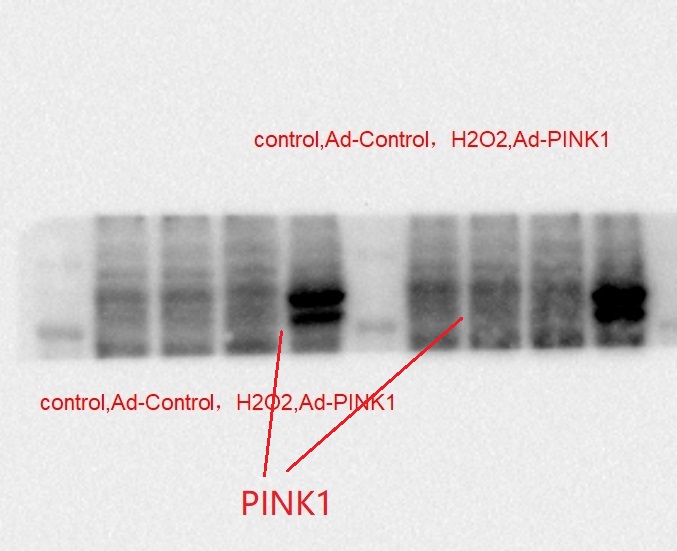

Supplement: Supplementary file 9 — original western blots [file 41420_2022_1021_MOESM9_ESM.jpg]

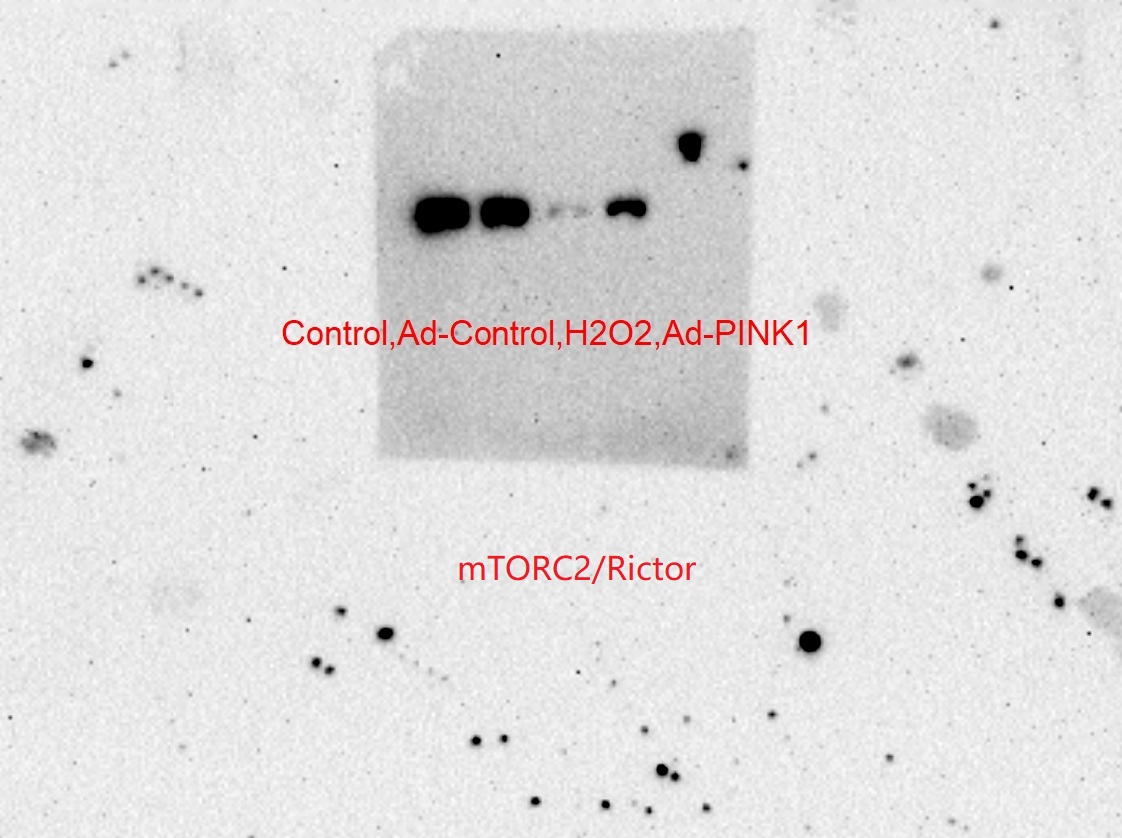

Supplement: Supplementary file 10 — original western blots [file 41420_2022_1021_MOESM10_ESM.jpg]

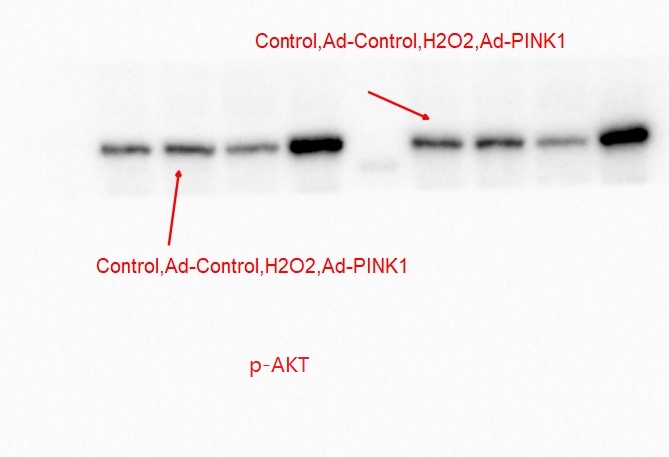

Supplement: Supplementary file 11 — original western blots [file 41420_2022_1021_MOESM11_ESM.jpg]

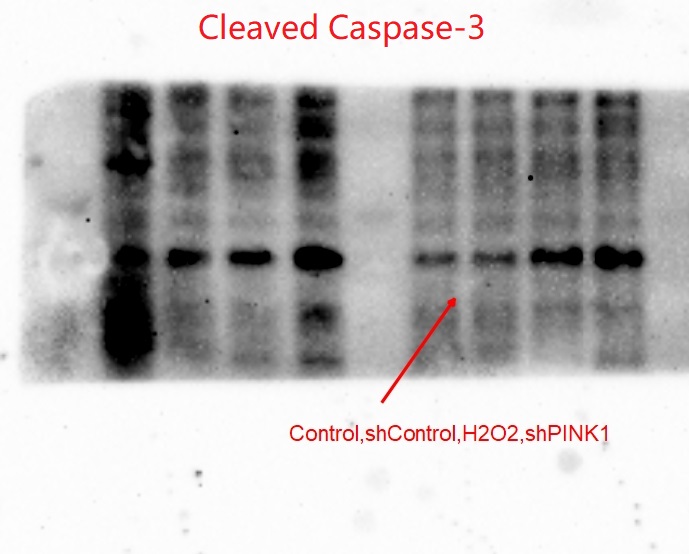

Supplement: Supplementary file 12 — original western blots [file 41420_2022_1021_MOESM12_ESM.jpg]

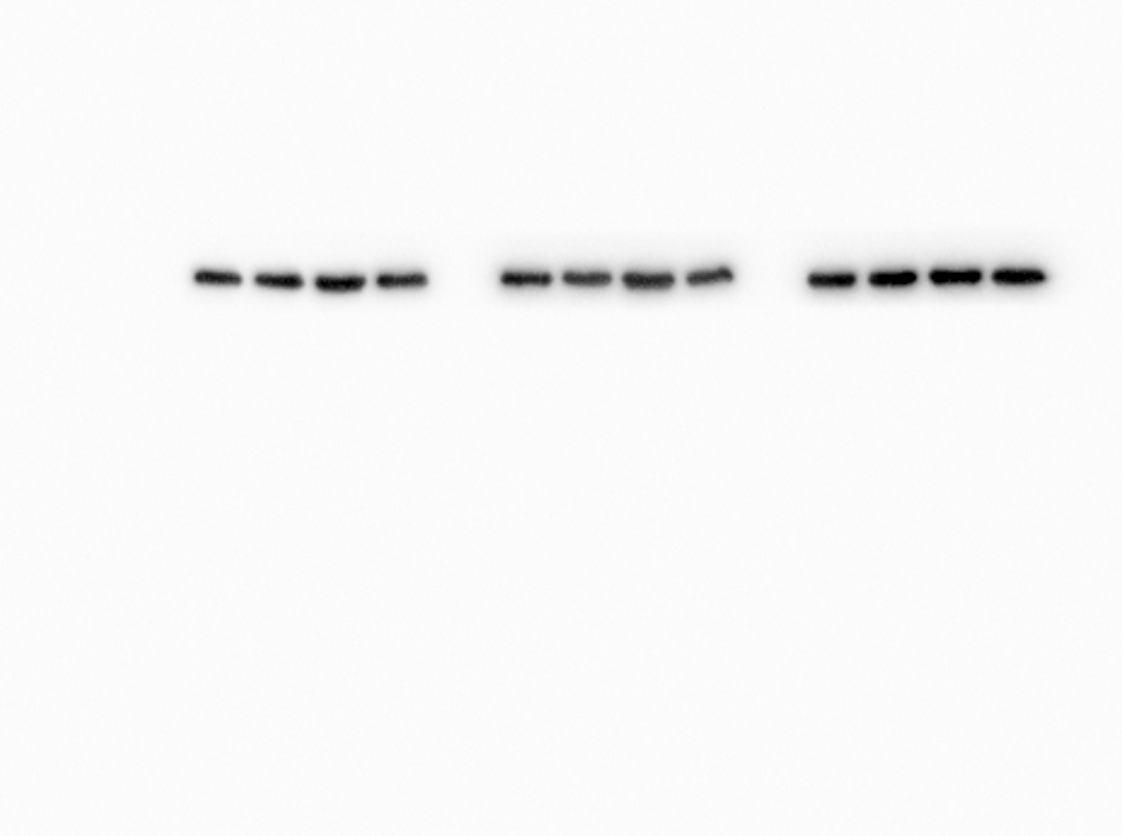

Supplement: Supplementary file 13 — original western blots [file 41420_2022_1021_MOESM13_ESM.jpg]

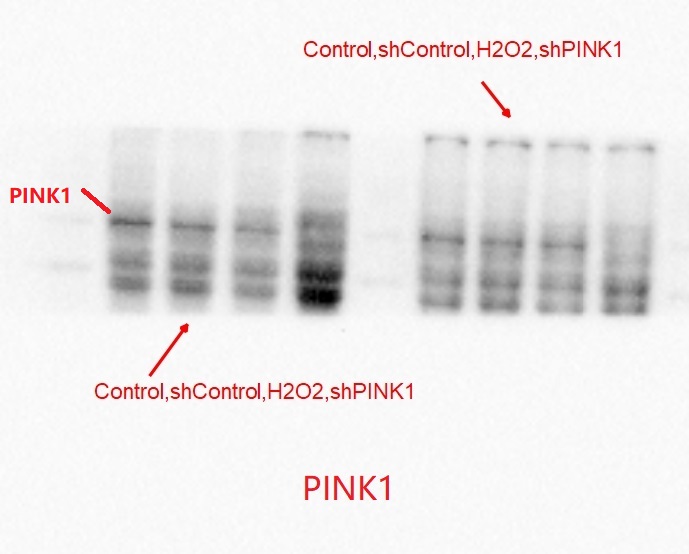

Supplement: Supplementary file 14 — original western blots [file 41420_2022_1021_MOESM14_ESM.jpg]

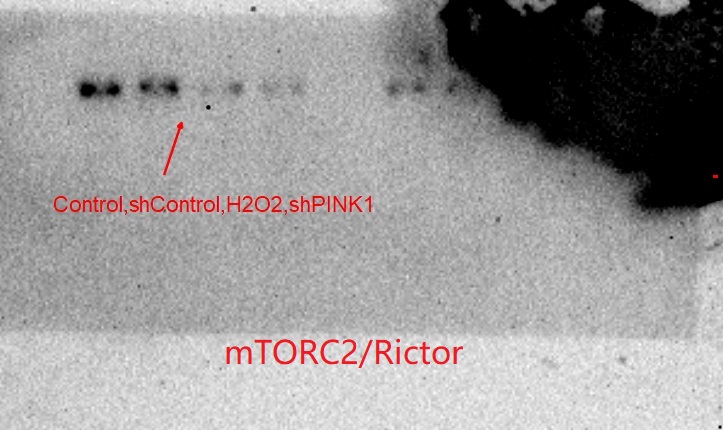

Supplement: Supplementary file 15 — original western blots [file 41420_2022_1021_MOESM15_ESM.jpg]

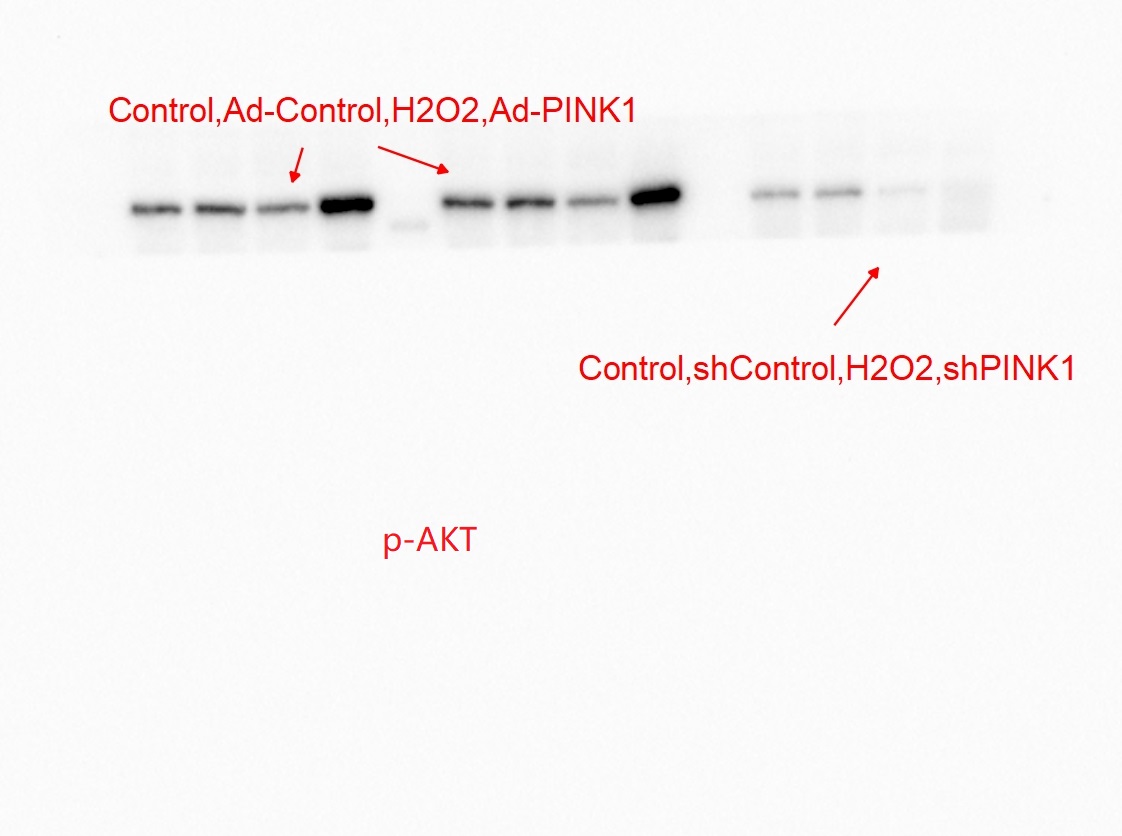

Supplement: Supplementary file 16 — original western blots [file 41420_2022_1021_MOESM16_ESM.jpg]

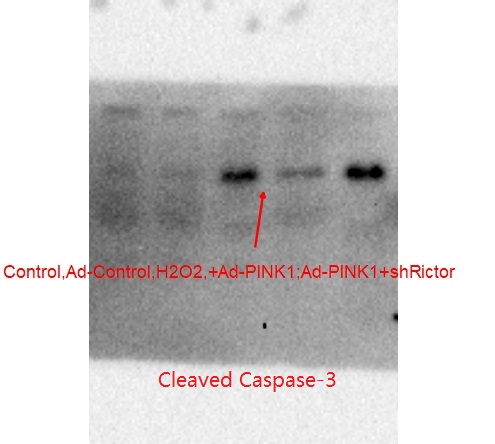

Supplement: Supplementary file 17 — original western blots [file 41420_2022_1021_MOESM17_ESM.jpg]

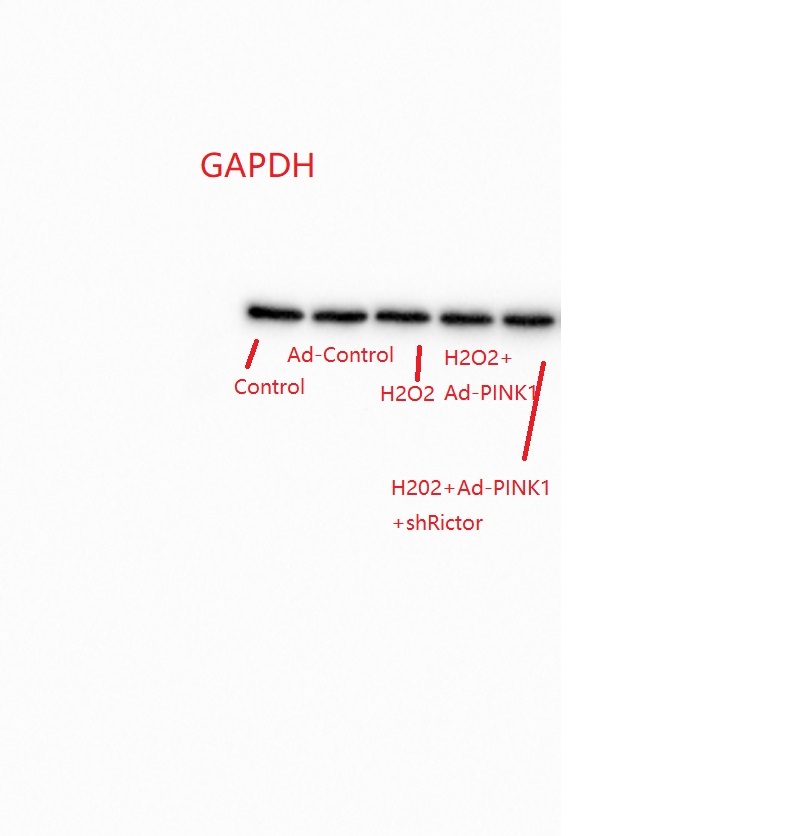

Supplement: Supplementary file 18 — original western blots [file 41420_2022_1021_MOESM18_ESM.jpg]

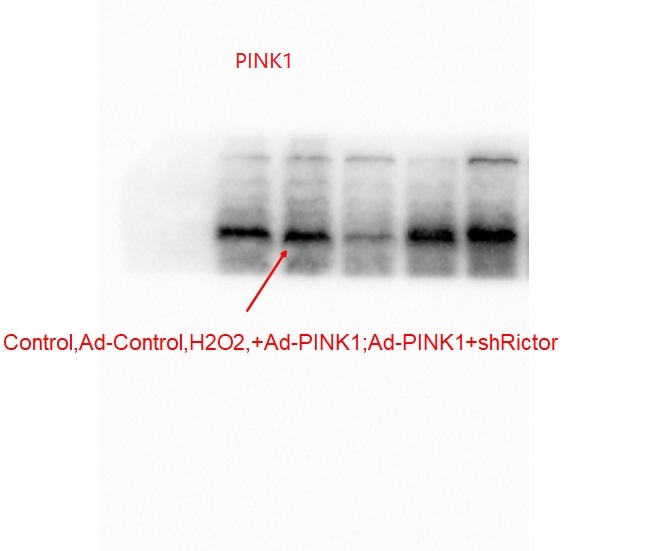

Supplement: Supplementary file 19 — original western blots [file 41420_2022_1021_MOESM19_ESM.jpg]

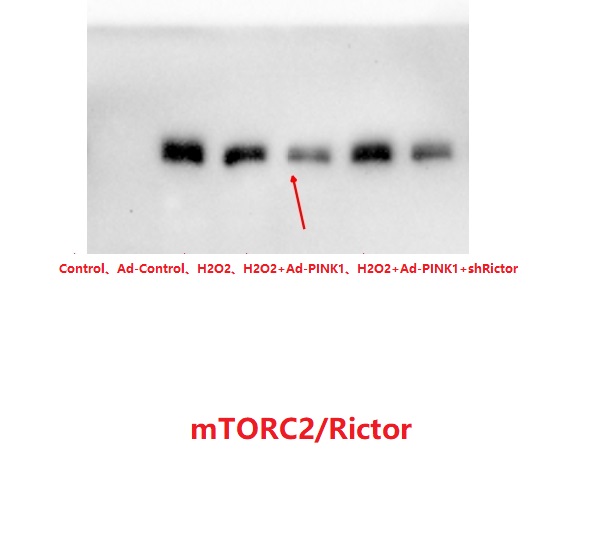

Supplement: Supplementary file 20 — original western blots [file 41420_2022_1021_MOESM20_ESM.jpg]

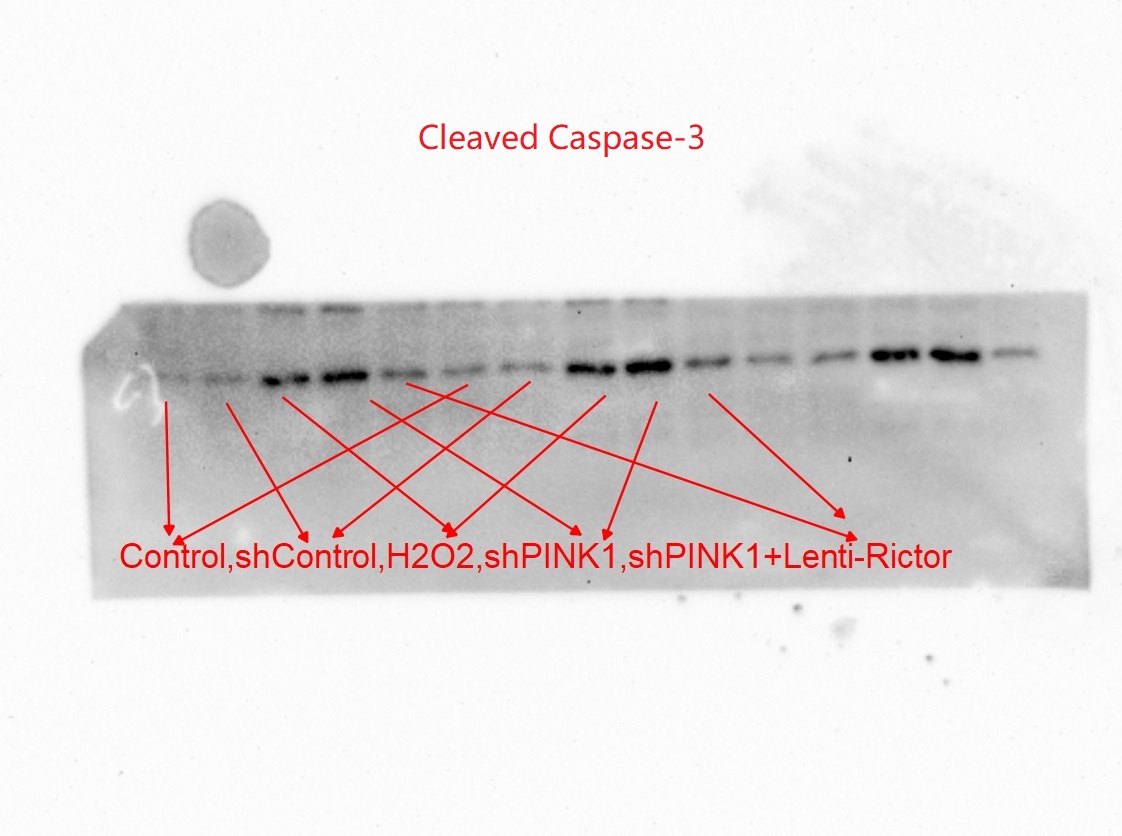

Supplement: Supplementary file 21 — original western blots [file 41420_2022_1021_MOESM21_ESM.jpg]

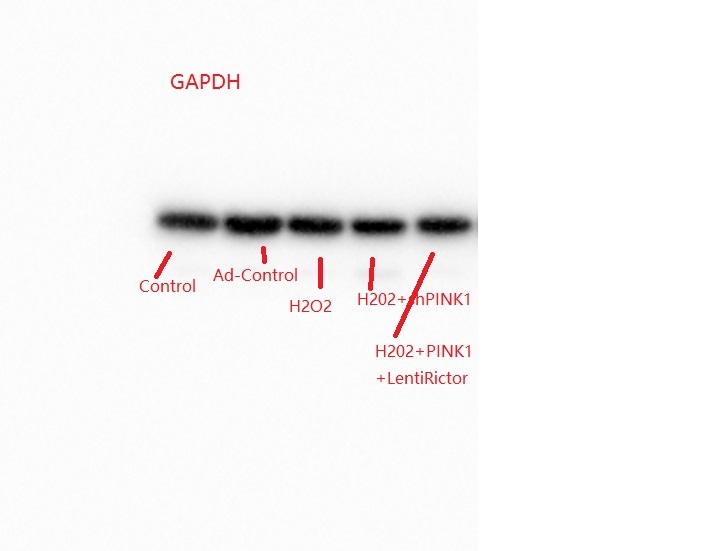

Supplement: Supplementary file 22 — original western blots [file 41420_2022_1021_MOESM22_ESM.jpg]

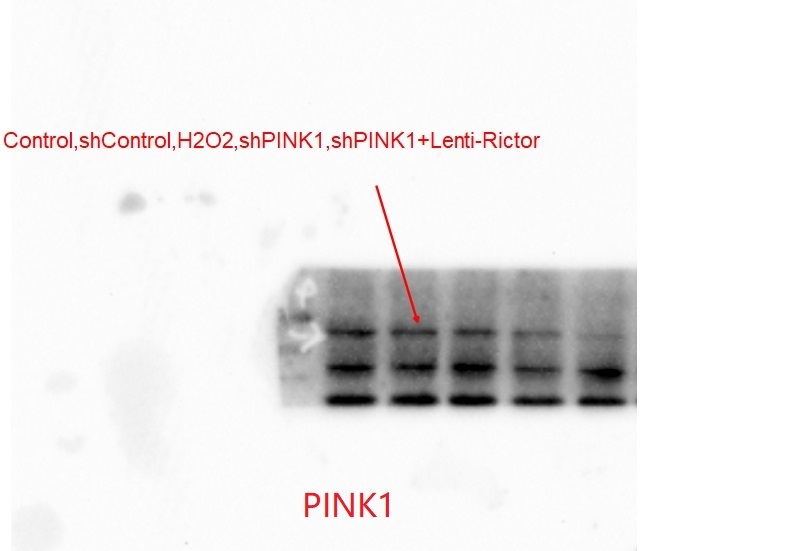

Supplement: Supplementary file 23 — original western blots [file 41420_2022_1021_MOESM23_ESM.jpg]

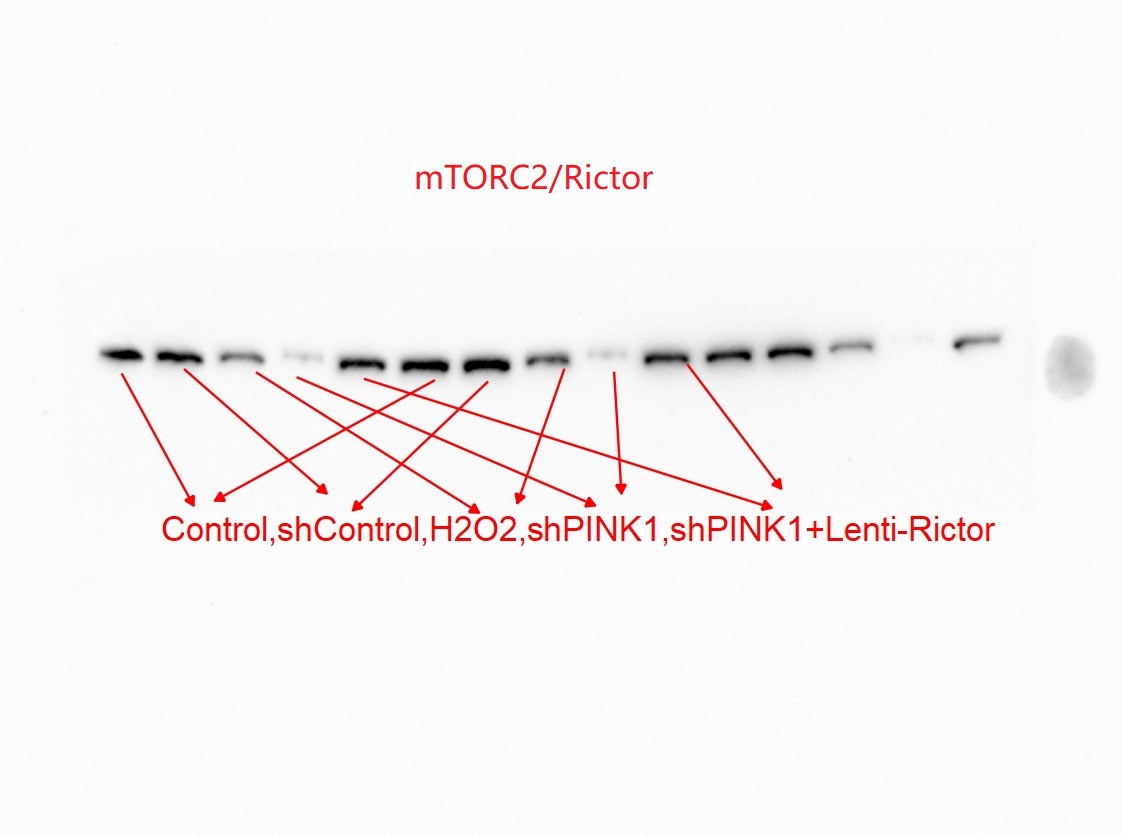

Supplement: Supplementary file 24 — original western blots [file 41420_2022_1021_MOESM24_ESM.jpg]

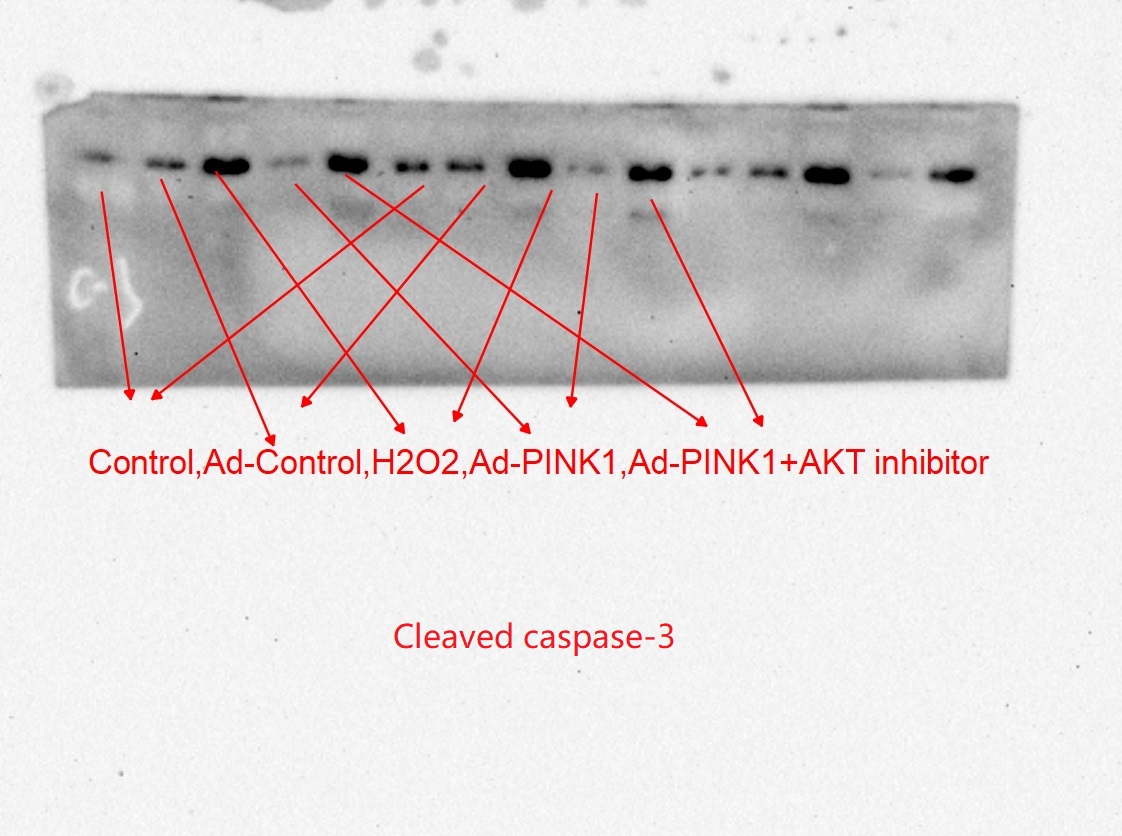

Supplement: Supplementary file 25 — original western blots [file 41420_2022_1021_MOESM25_ESM.jpg]

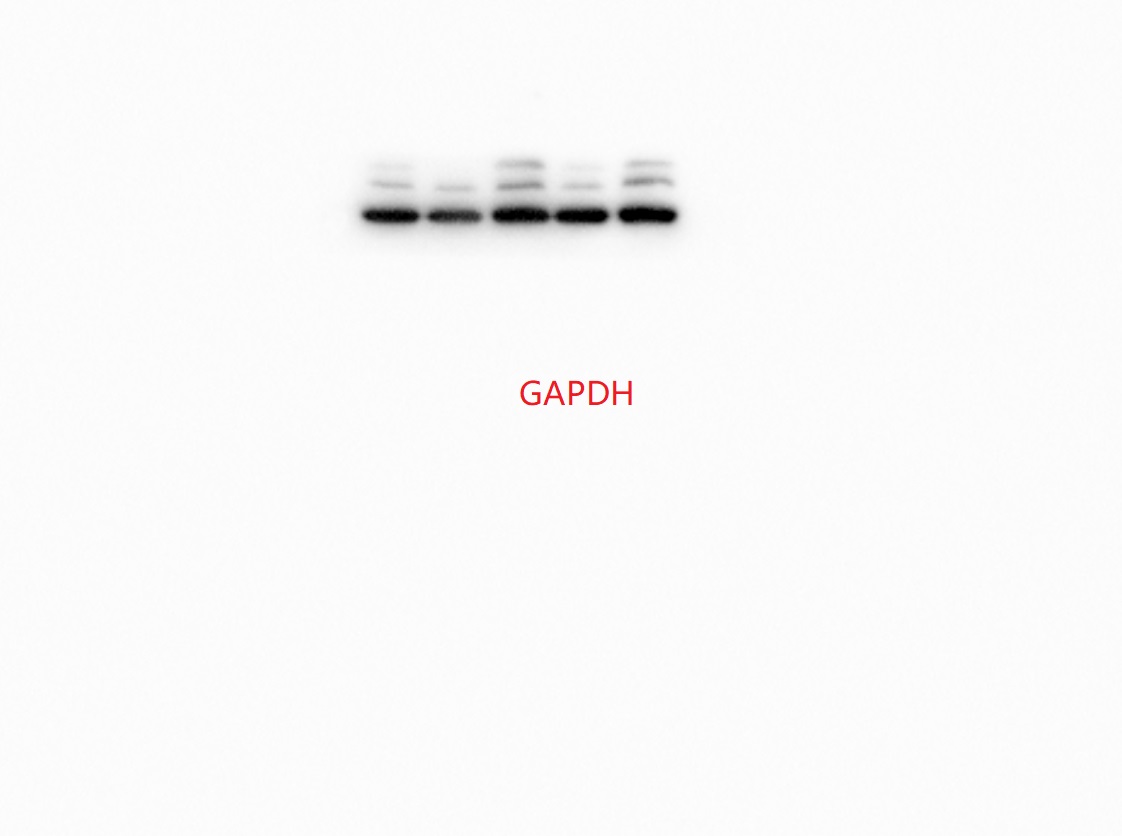

Supplement: Supplementary file 26 — original western blots [file 41420_2022_1021_MOESM26_ESM.jpg]

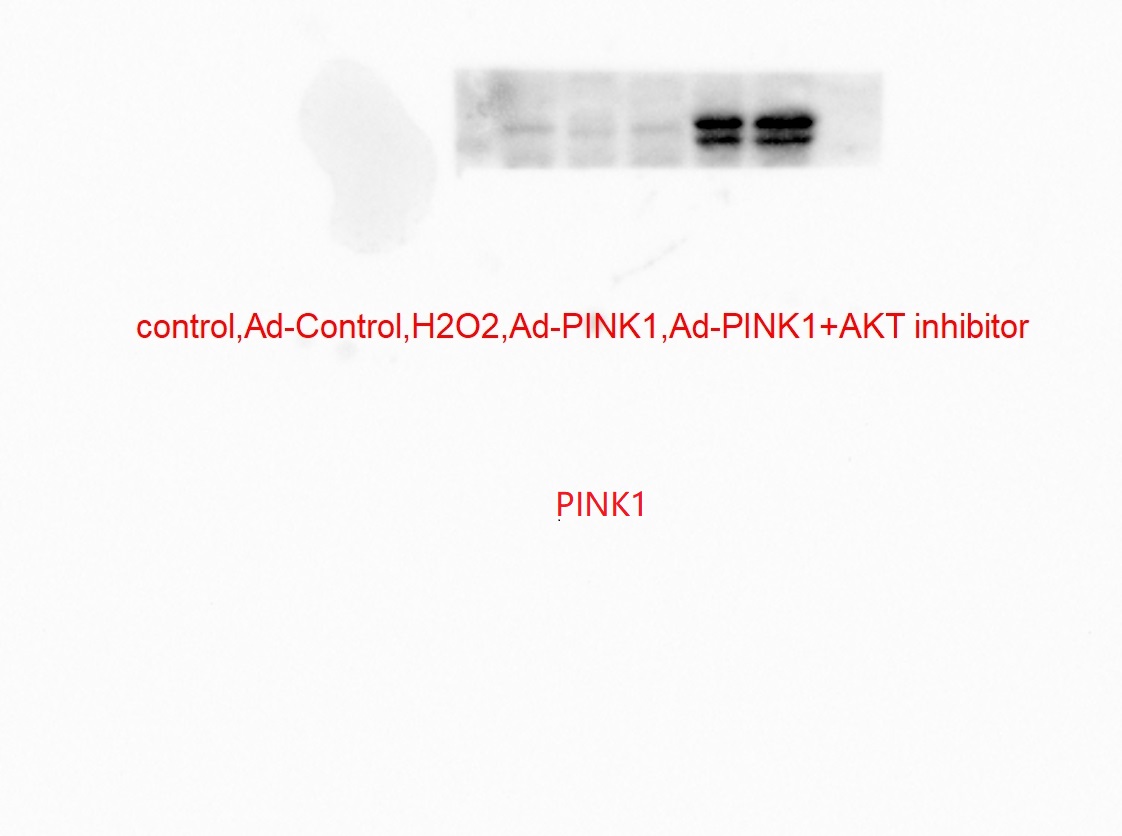

Supplement: Supplementary file 27 — original western blots [file 41420_2022_1021_MOESM27_ESM.jpg]

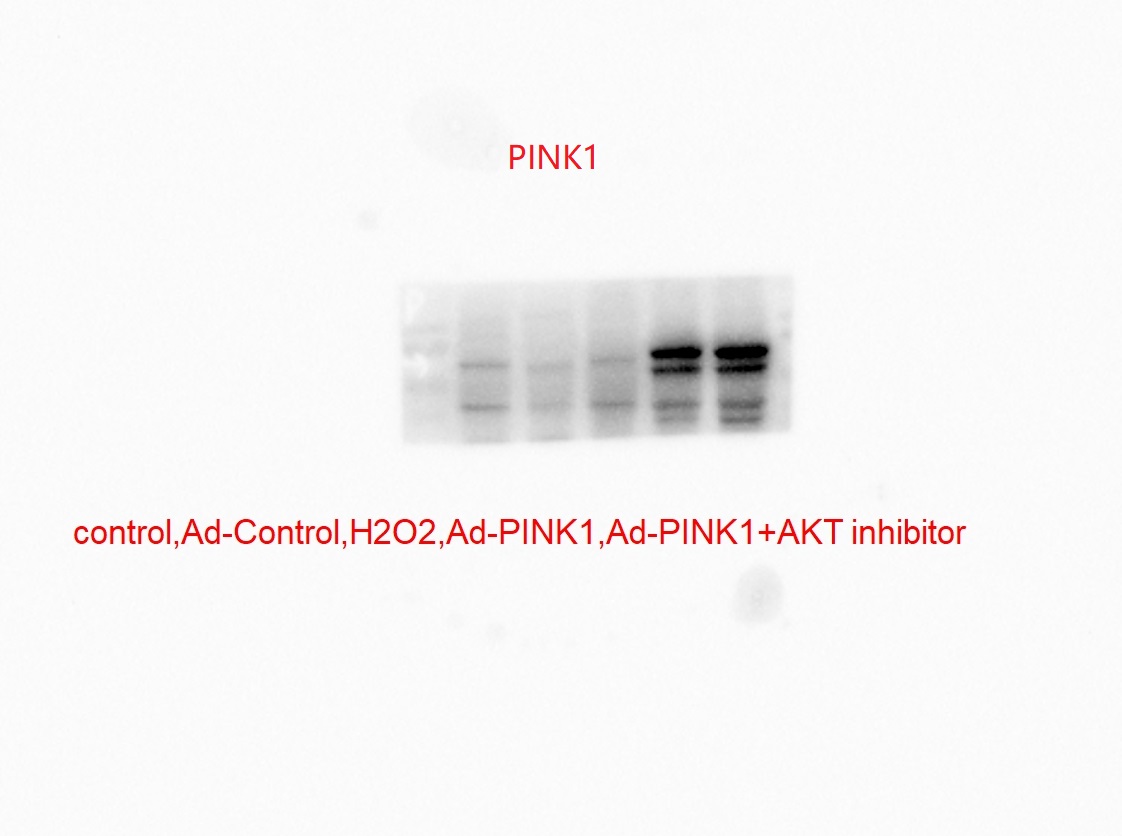

Supplement: Supplementary file 28 — original western blots [file 41420_2022_1021_MOESM28_ESM.jpg]

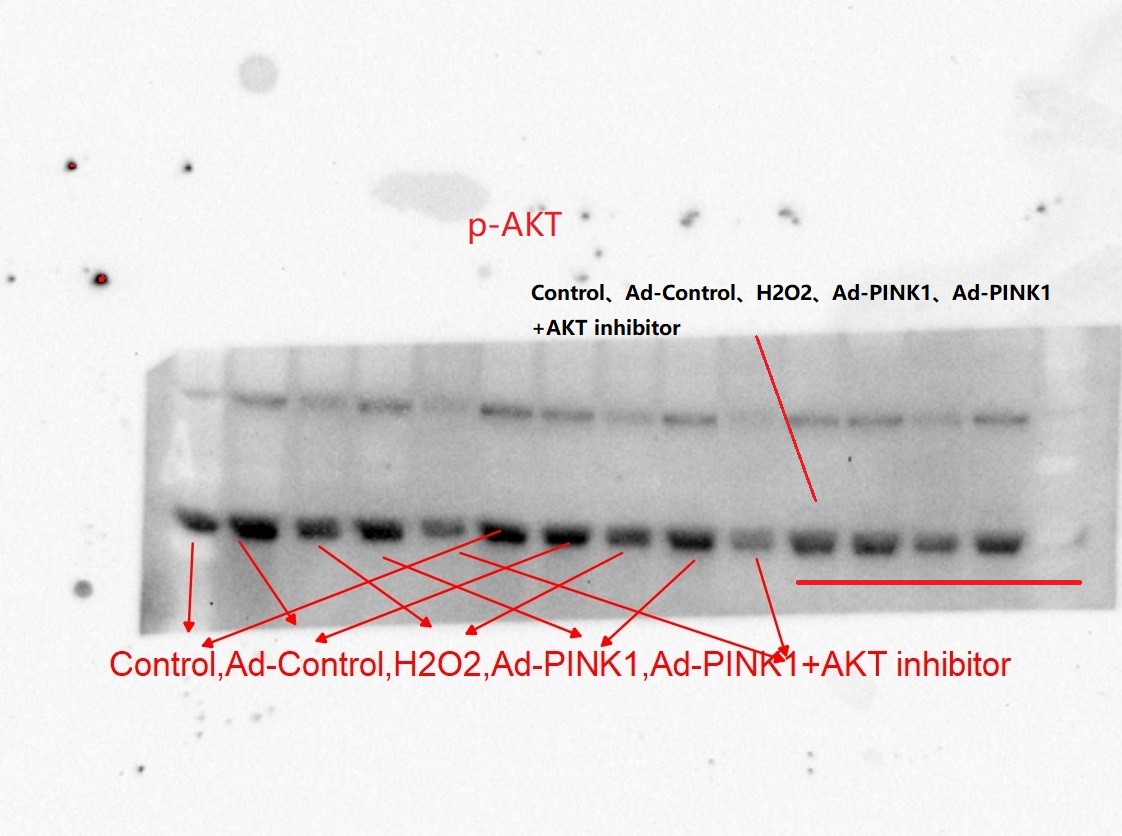

Supplement: Supplementary file 29 — original western blots [file 41420_2022_1021_MOESM29_ESM.jpg]

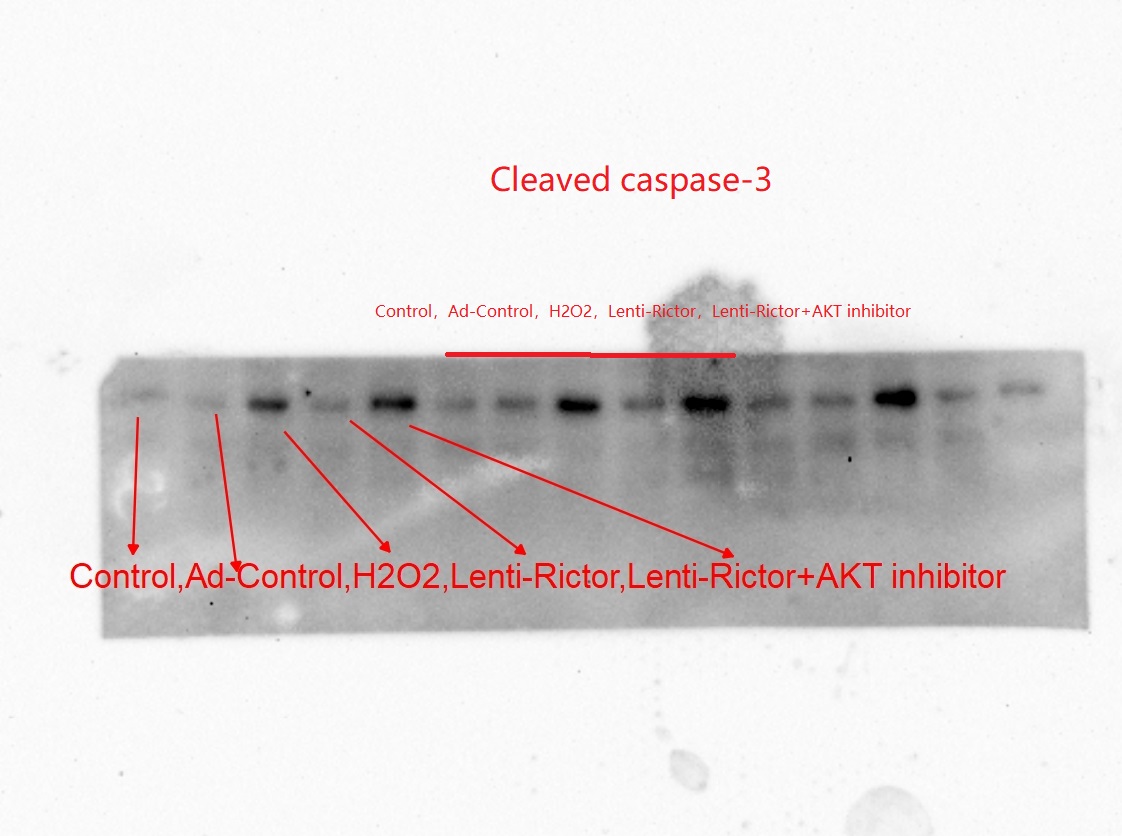

Supplement: Supplementary file 30 — original western blots [file 41420_2022_1021_MOESM30_ESM.jpg]

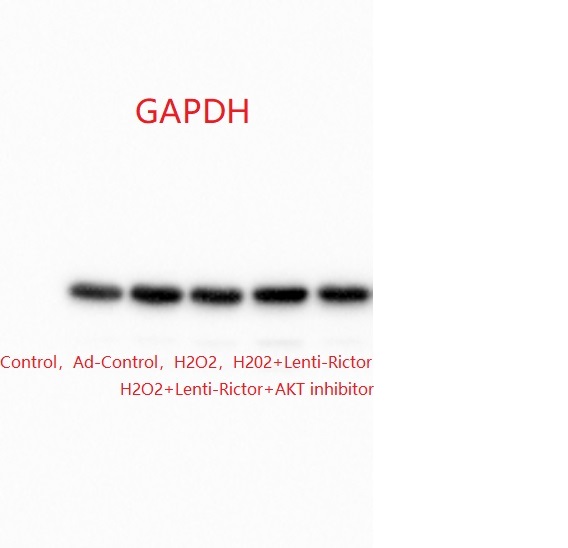

Supplement: Supplementary file 31 — original western blots [file 41420_2022_1021_MOESM31_ESM.jpg]

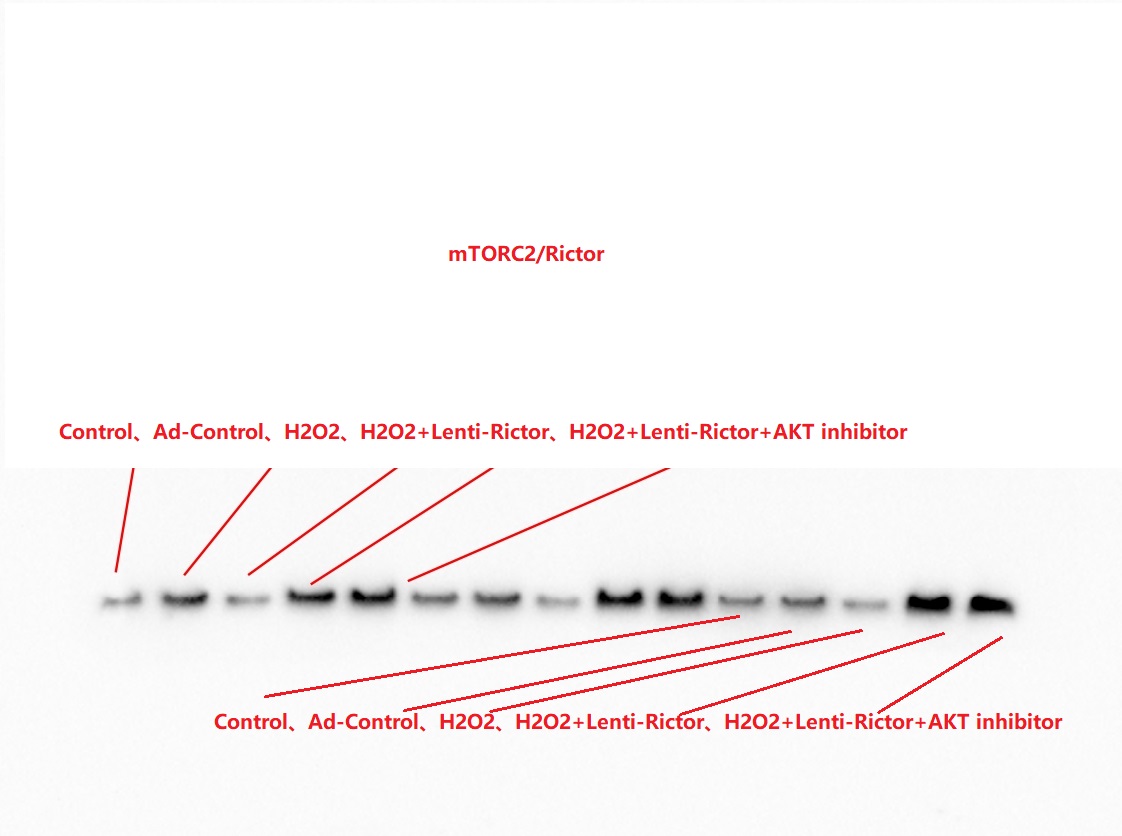

Supplement: Supplementary file 32 — original western blots [file 41420_2022_1021_MOESM32_ESM.jpg]

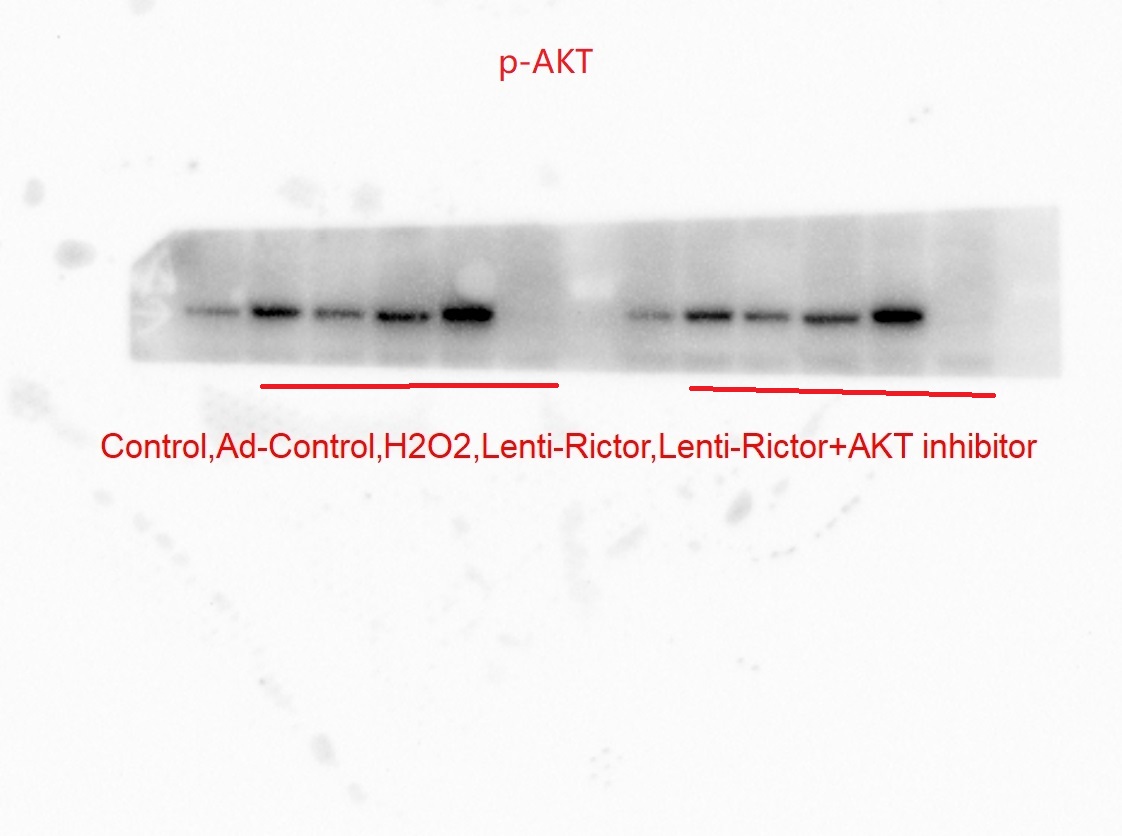

Supplement: Supplementary file 33 — original western blots [file 41420_2022_1021_MOESM33_ESM.jpg]

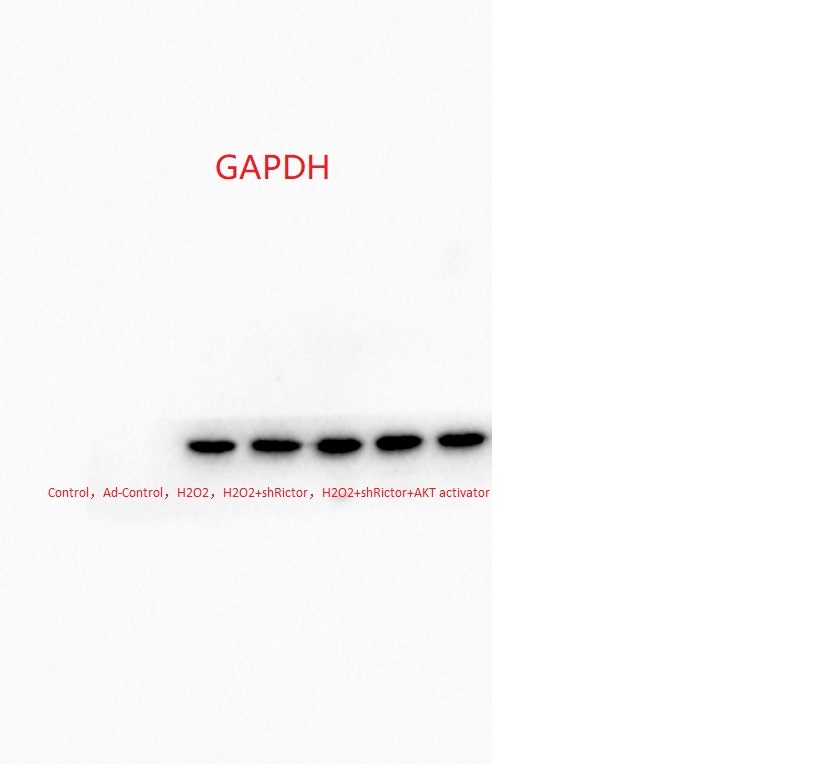

Supplement: Supplementary file 34 — original western blots [file 41420_2022_1021_MOESM34_ESM.jpg]

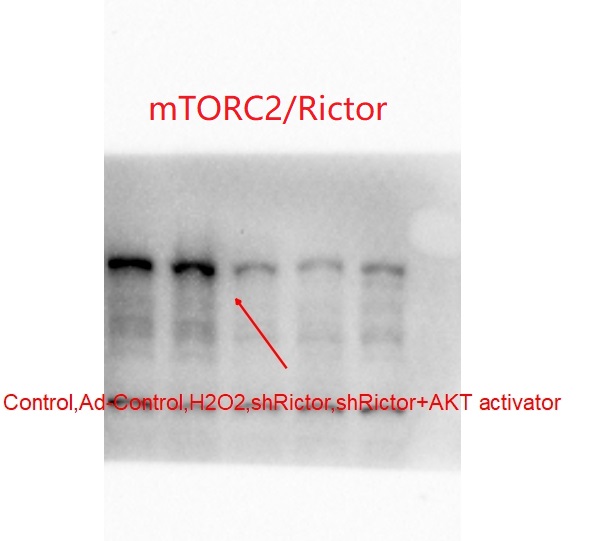

Supplement: Supplementary file 35 — original western blots [file 41420_2022_1021_MOESM35_ESM.jpg]

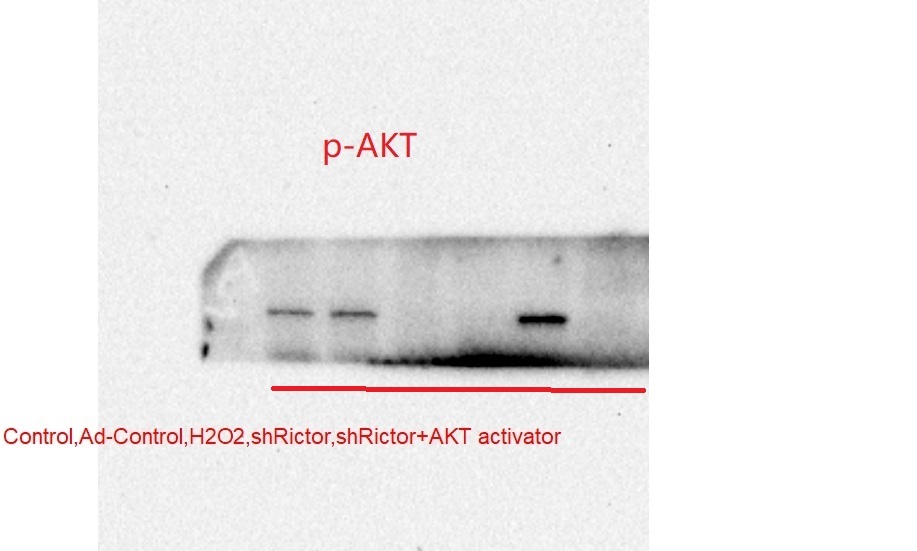

Supplement: Supplementary file 36 — original western blots [file 41420_2022_1021_MOESM36_ESM.jpg]

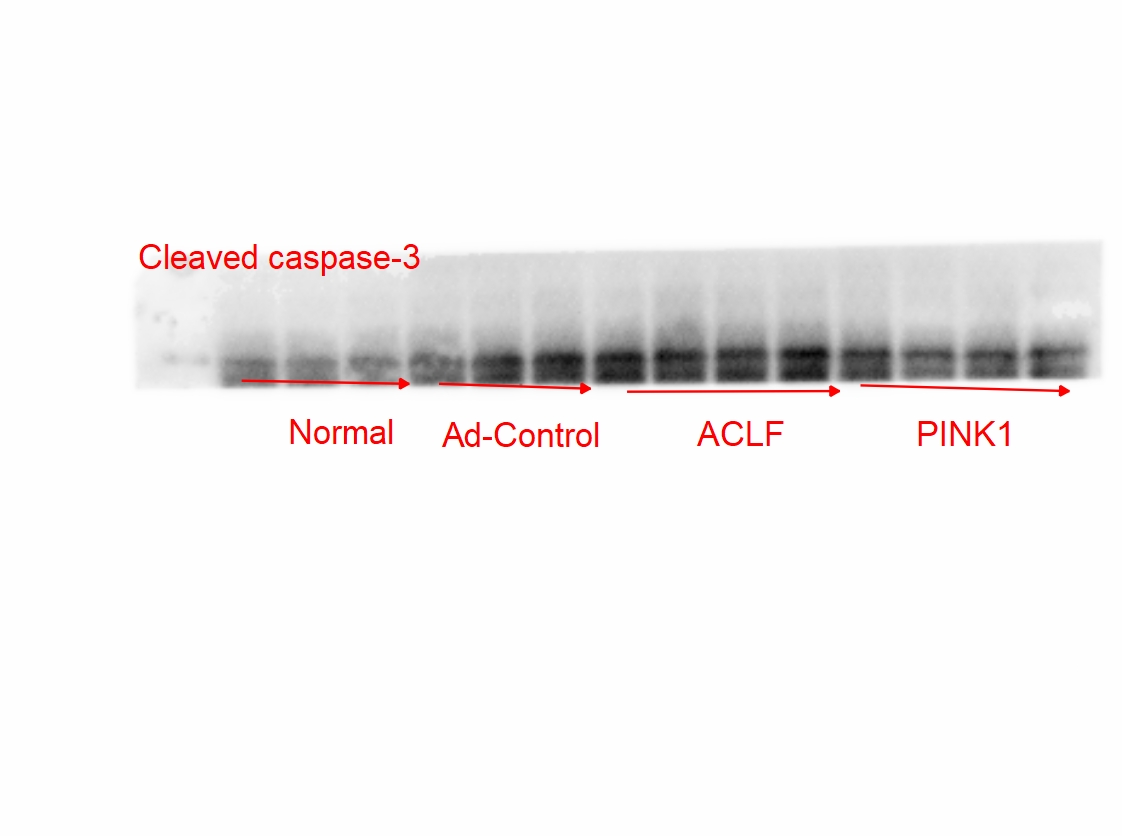

Supplement: Supplementary file 37 — original western blots [file 41420_2022_1021_MOESM37_ESM.jpg]

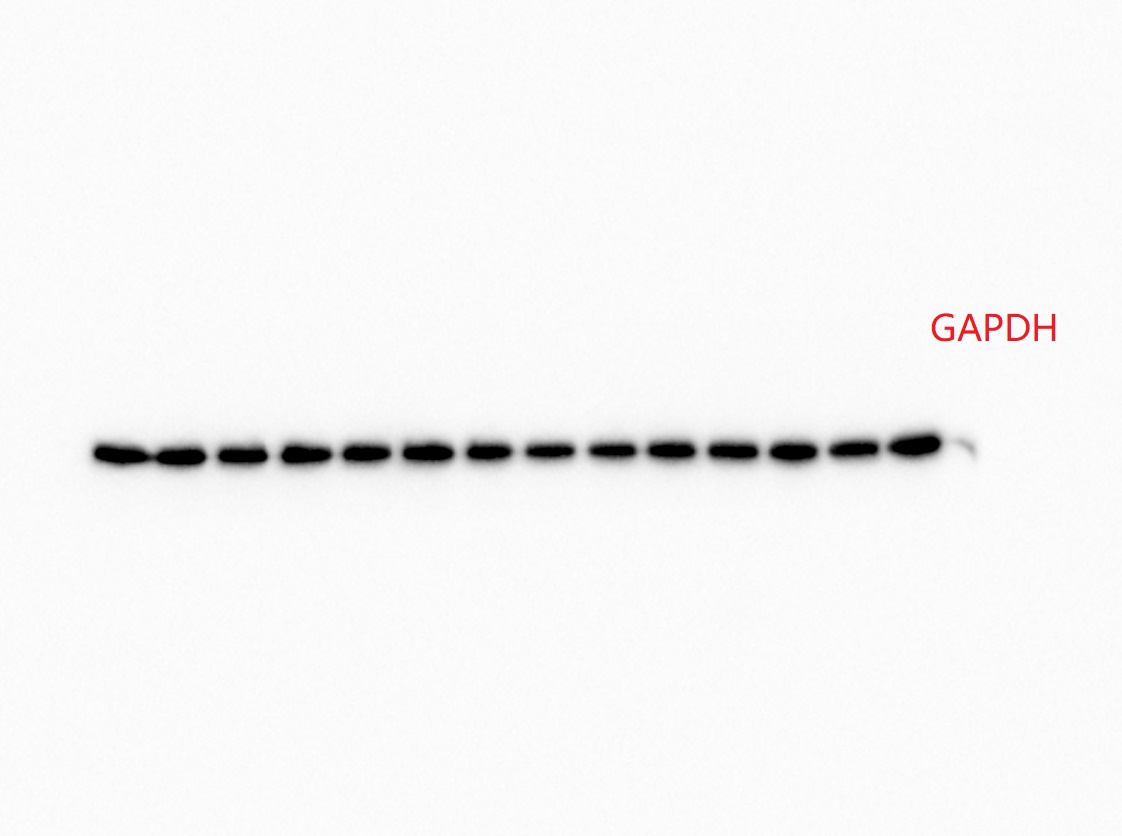

Supplement: Supplementary file 38 — original western blots [file 41420_2022_1021_MOESM38_ESM.jpg]

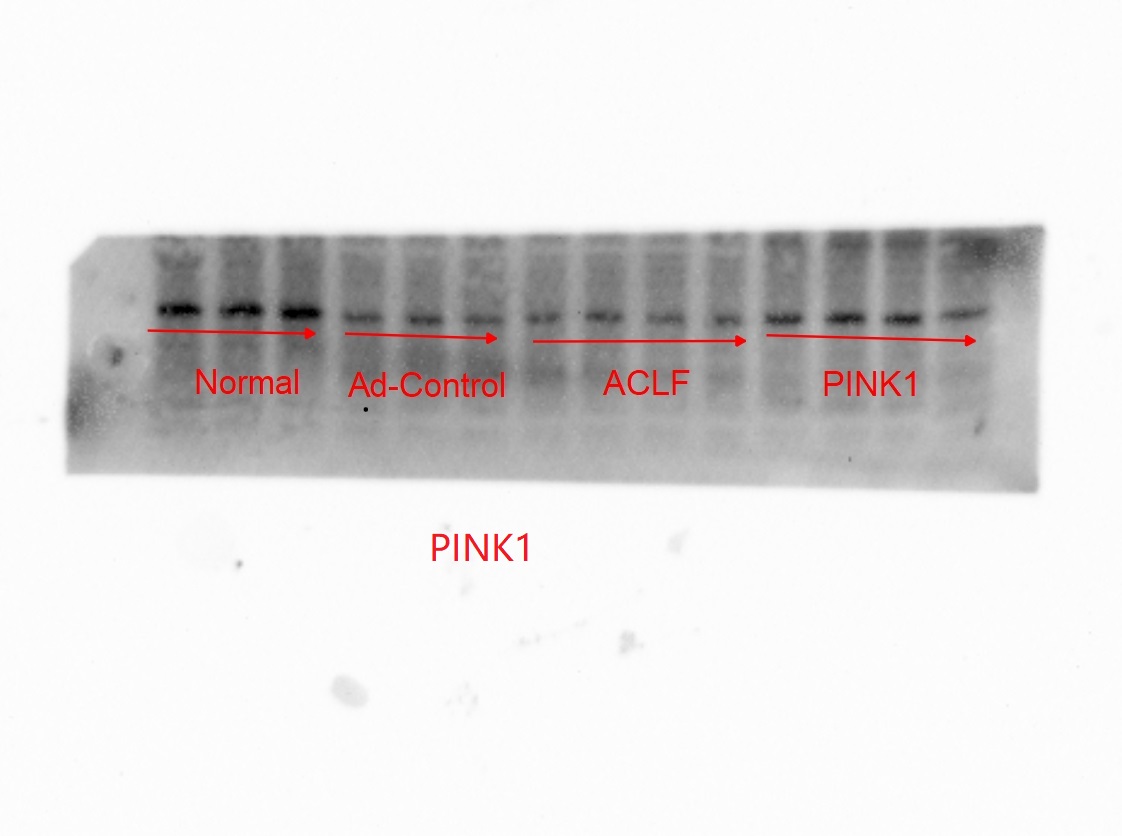

Supplement: Supplementary file 39 — original western blots [file 41420_2022_1021_MOESM39_ESM.jpg]

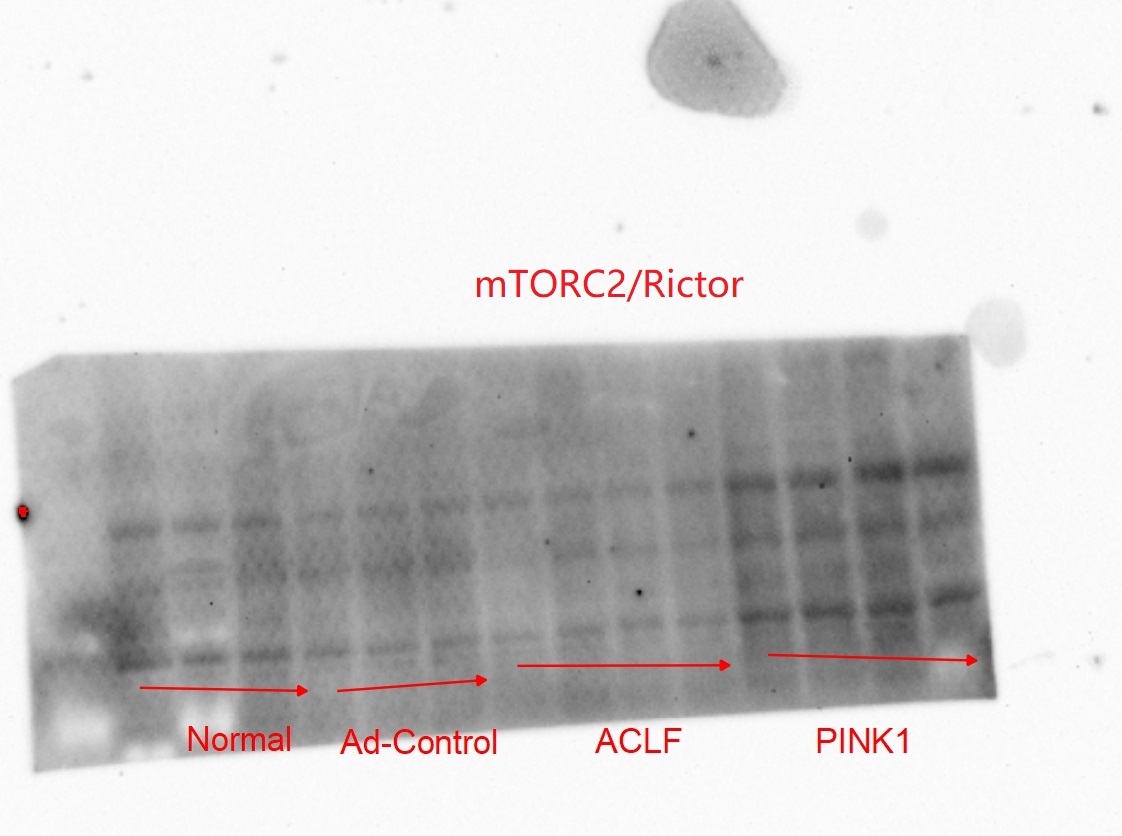

Supplement: Supplementary file 40 — original western blots [file 41420_2022_1021_MOESM40_ESM.jpg]

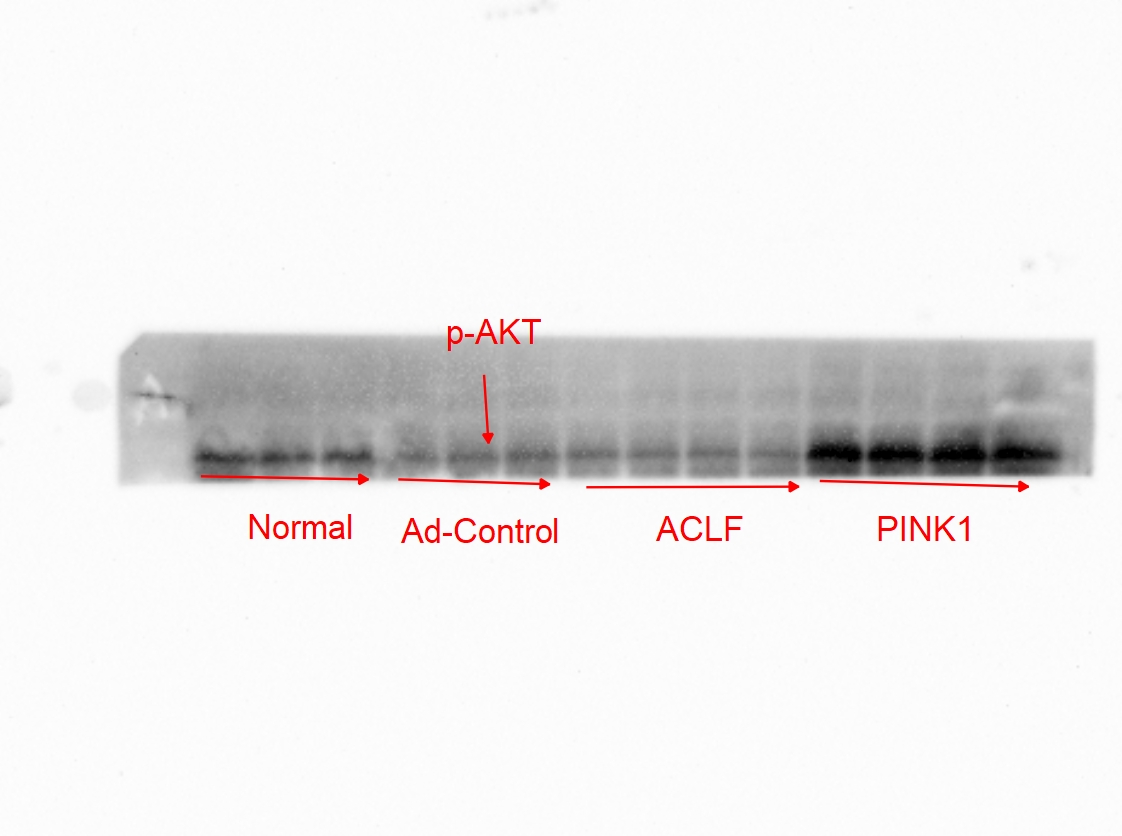

Supplement: Supplementary file 41 — original western blots [file 41420_2022_1021_MOESM41_ESM.jpg]

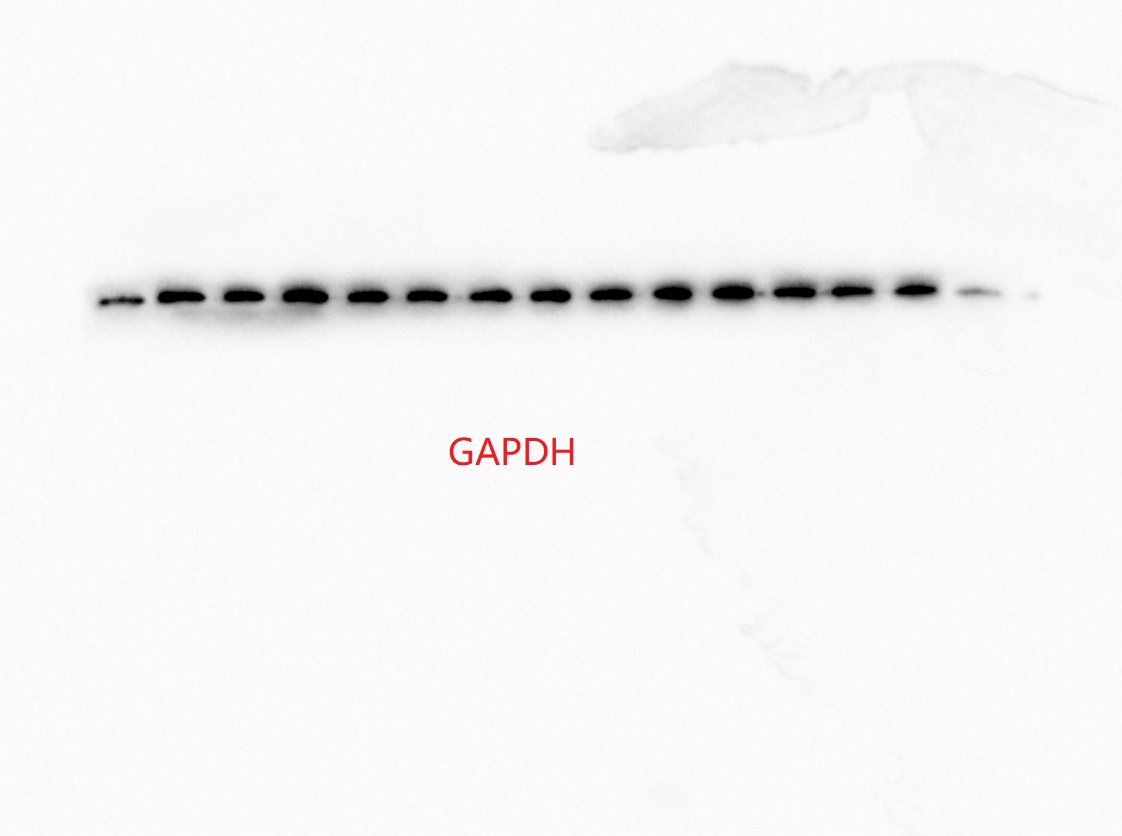

Supplement: Supplementary file 42 — original western blots [file 41420_2022_1021_MOESM42_ESM.jpg]

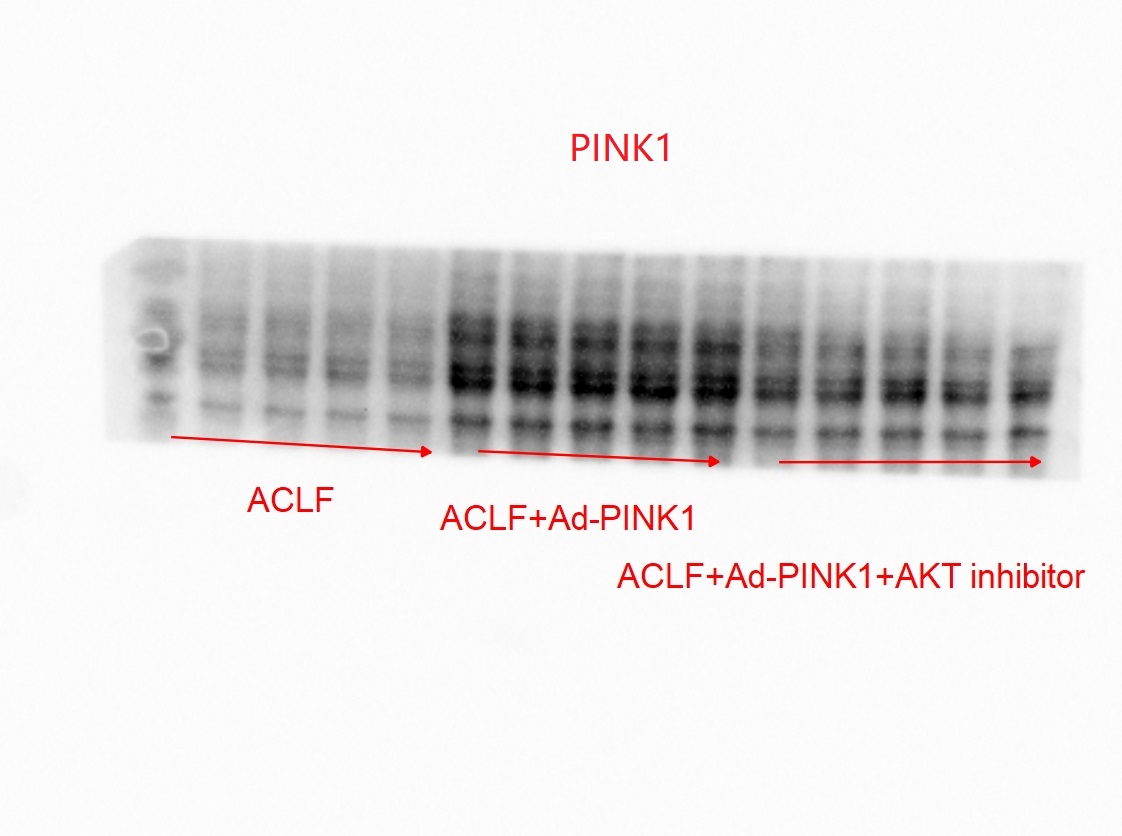

Supplement: Supplementary file 43 — original western blots [file 41420_2022_1021_MOESM43_ESM.jpg]

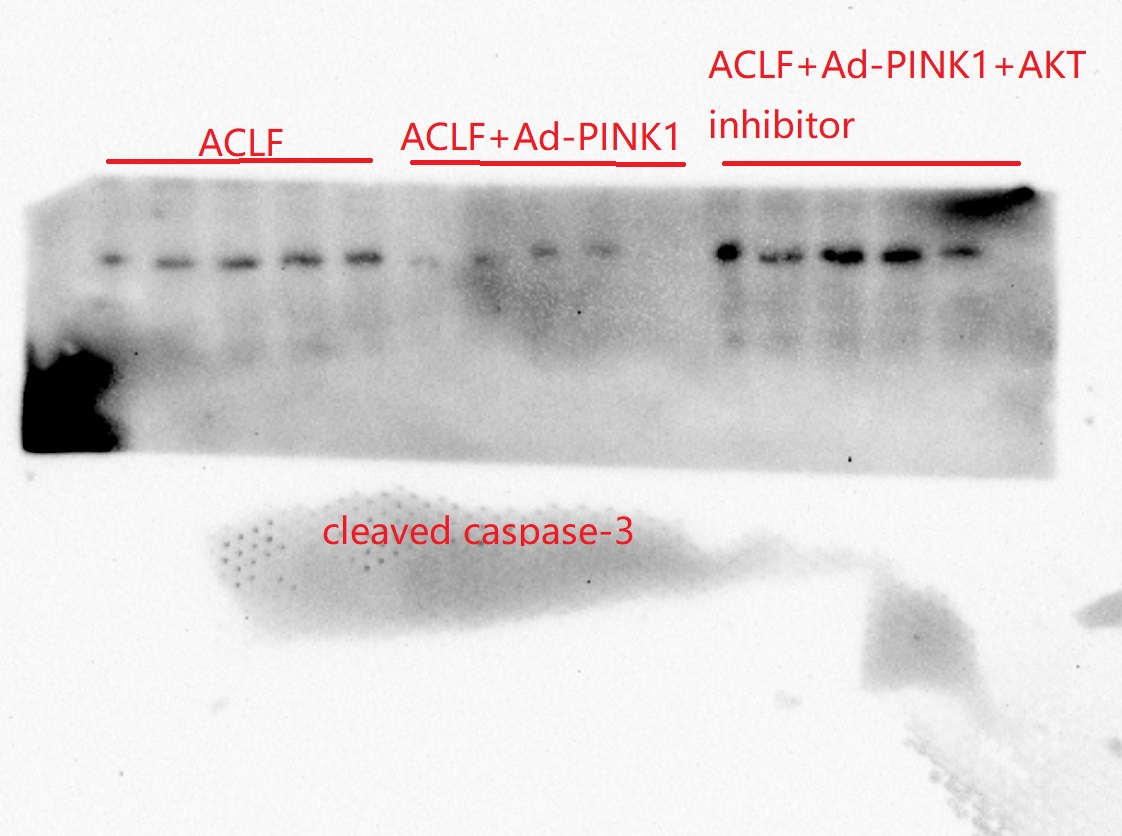

Supplement: Supplementary file 44 — original western blots [file 41420_2022_1021_MOESM44_ESM.jpg]

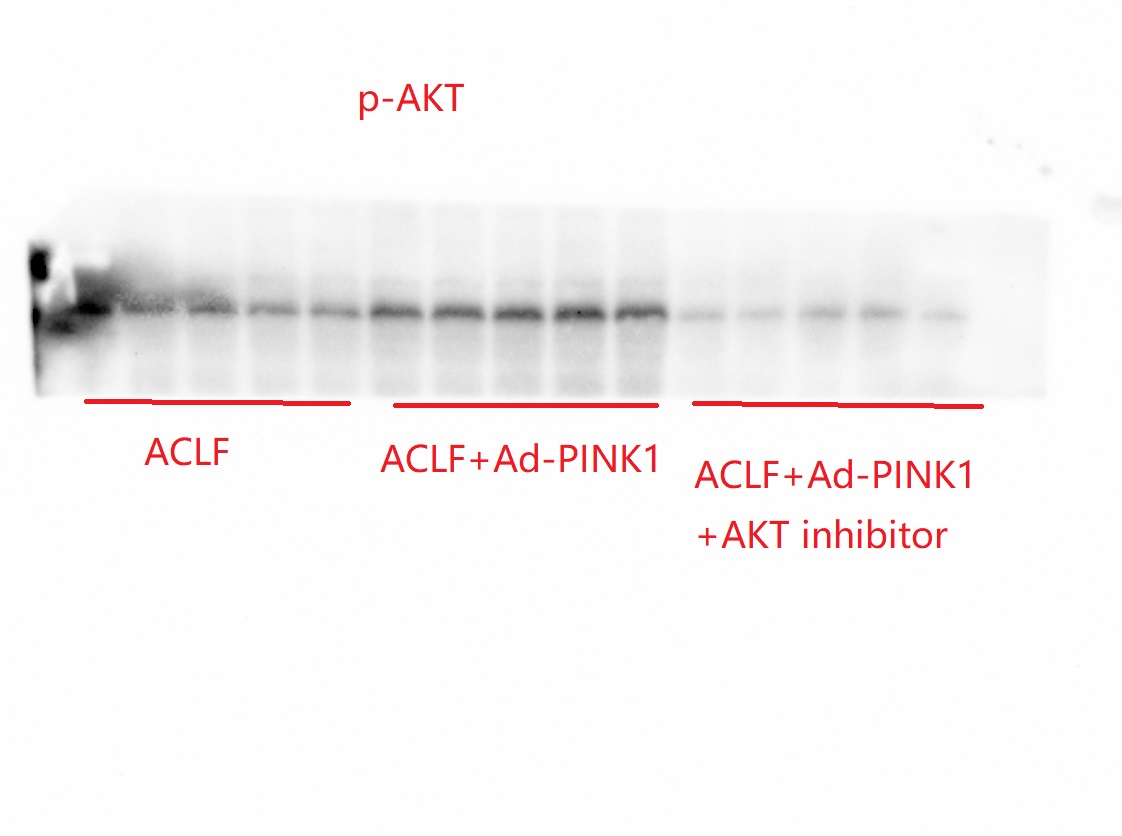

Supplement: Supplementary file 45 — original western blots [file 41420_2022_1021_MOESM45_ESM.jpg]

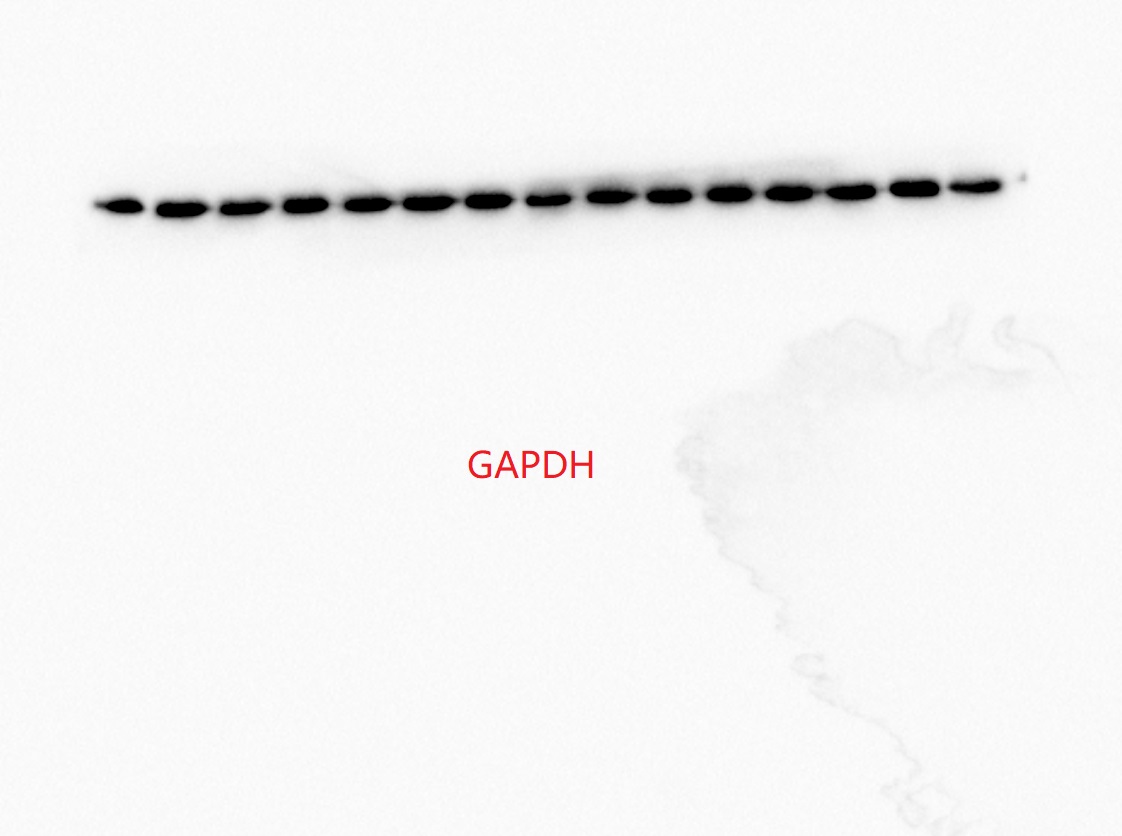

Supplement: Supplementary file 46 — original western blots [file 41420_2022_1021_MOESM46_ESM.jpg]

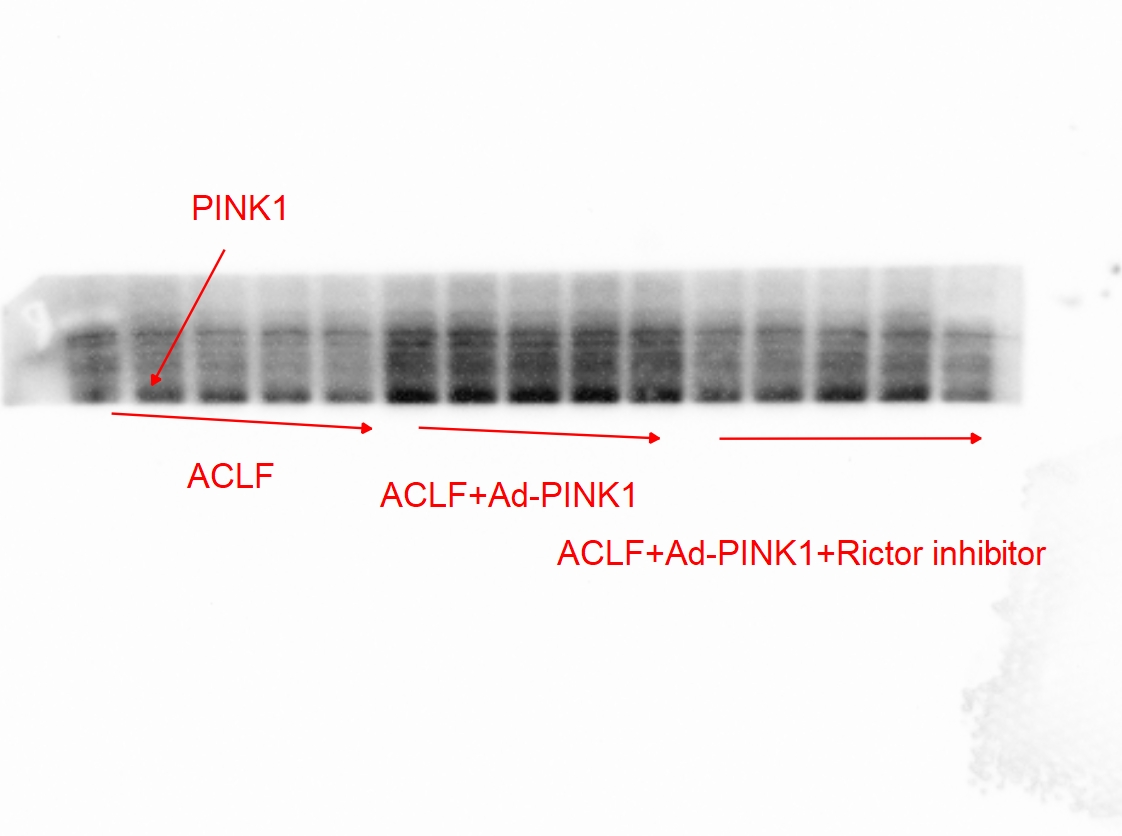

Supplement: Supplementary file 47 — original western blots [file 41420_2022_1021_MOESM47_ESM.jpg]

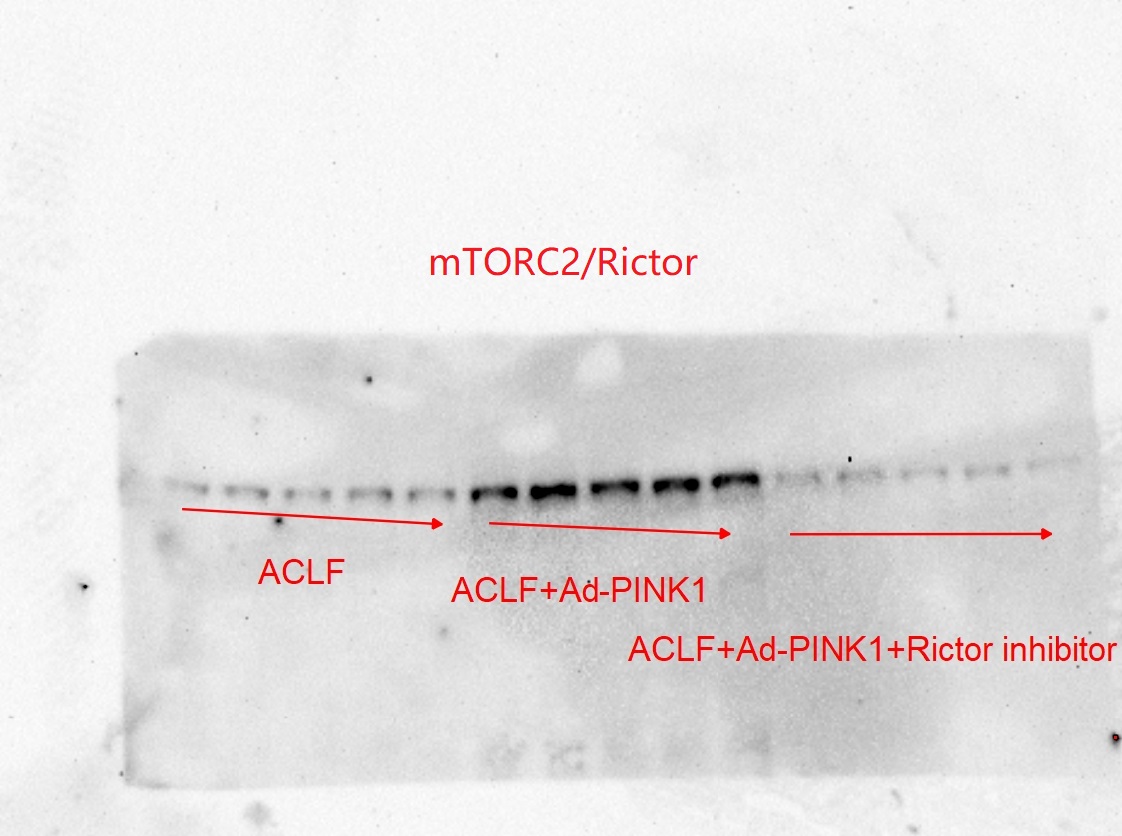

Supplement: Supplementary file 48 — original western blots [file 41420_2022_1021_MOESM48_ESM.jpg]

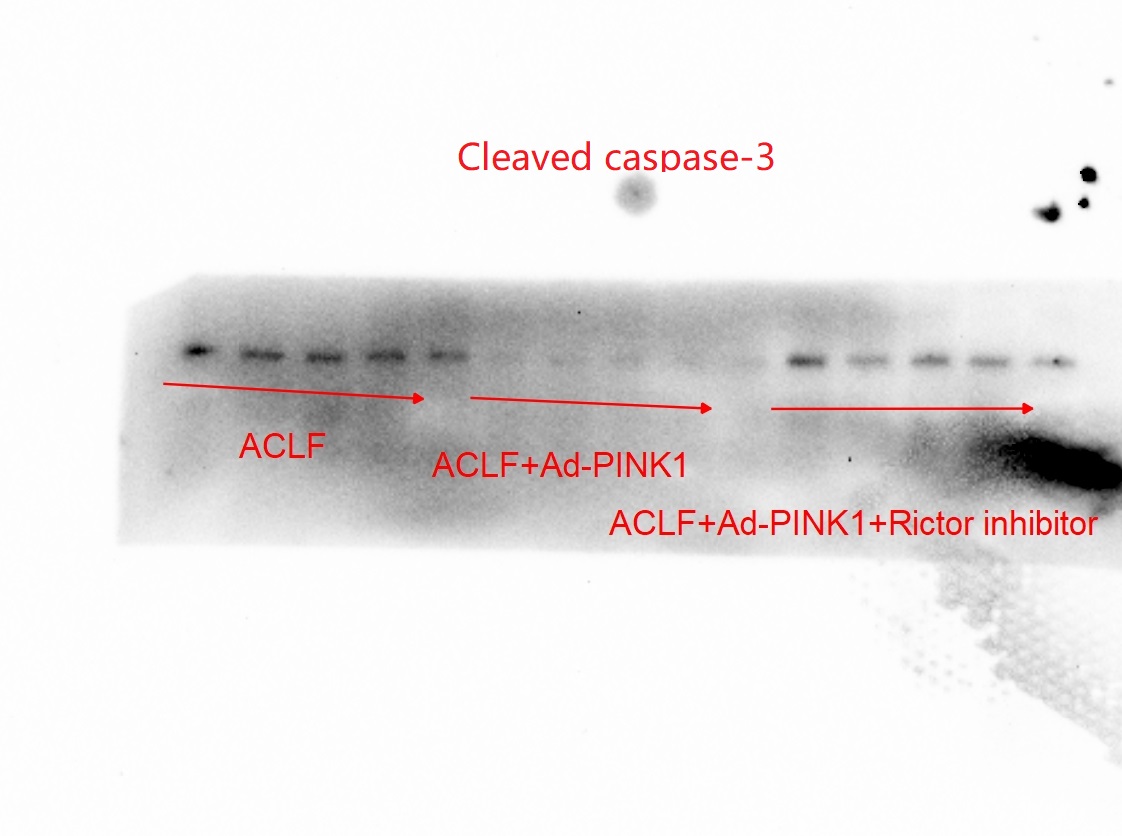

Supplement: Supplementary file 49 — original western blots [file 41420_2022_1021_MOESM49_ESM.jpg]

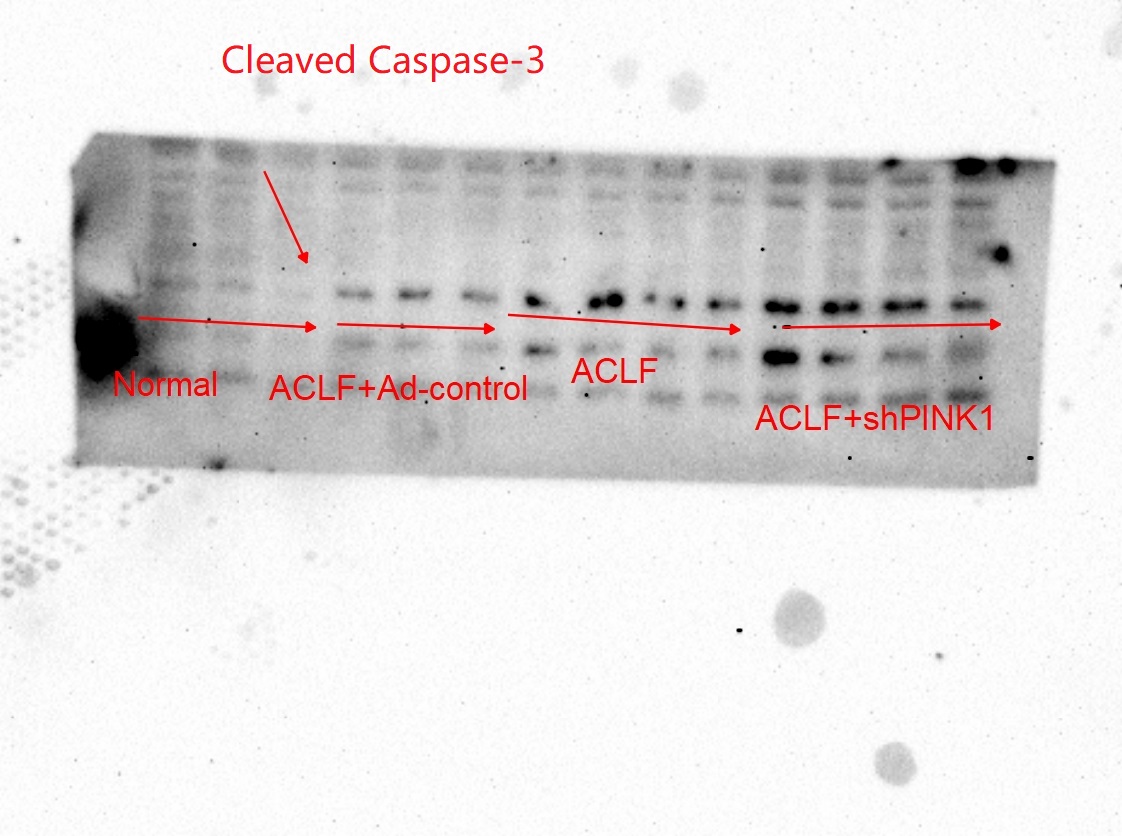

Supplement: Supplementary file 50 — original western blots [file 41420_2022_1021_MOESM50_ESM.jpg]

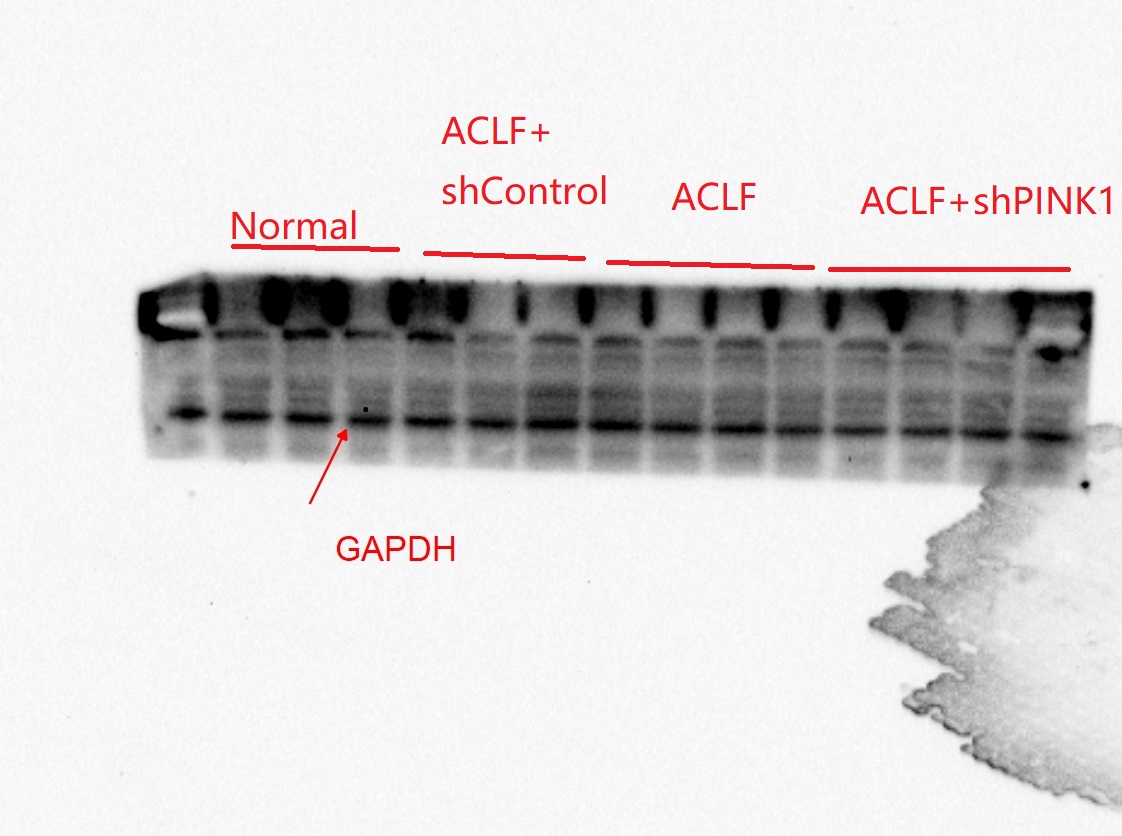

Supplement: Supplementary file 51 — original western blots [file 41420_2022_1021_MOESM51_ESM.jpg]

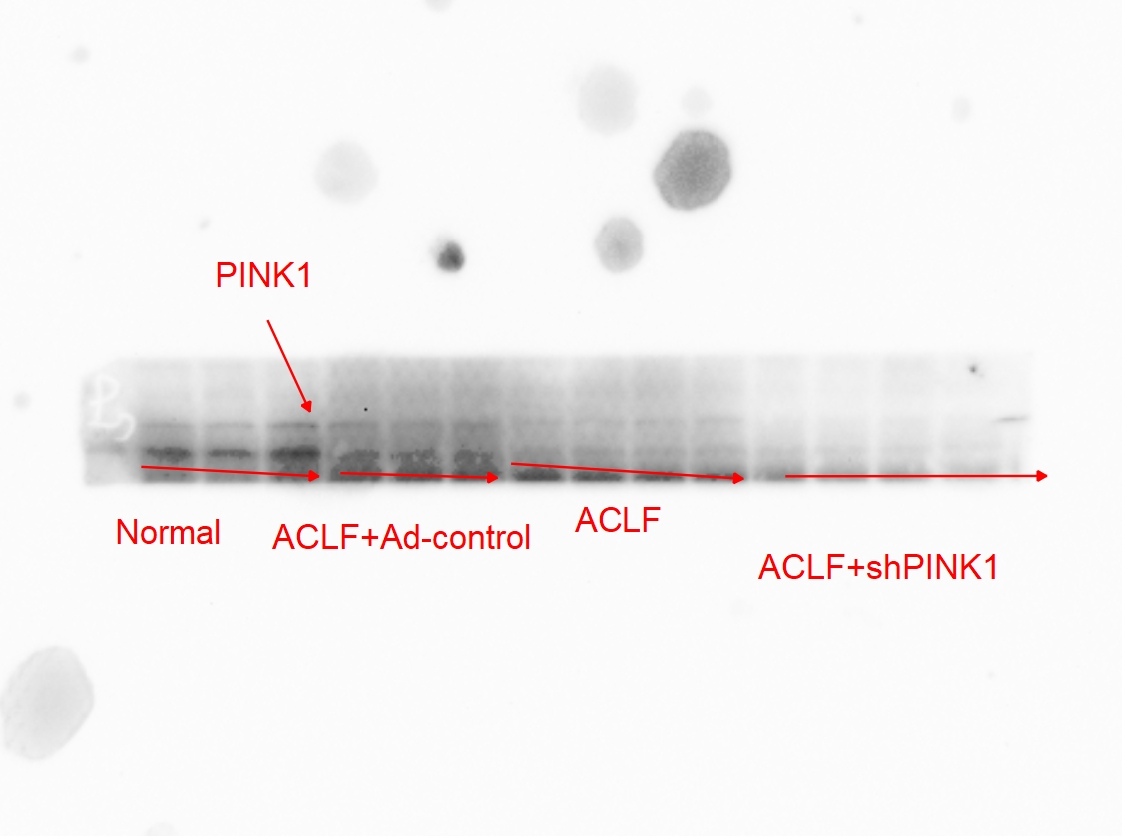

Supplement: Supplementary file 52 — original western blots [file 41420_2022_1021_MOESM52_ESM.jpg]

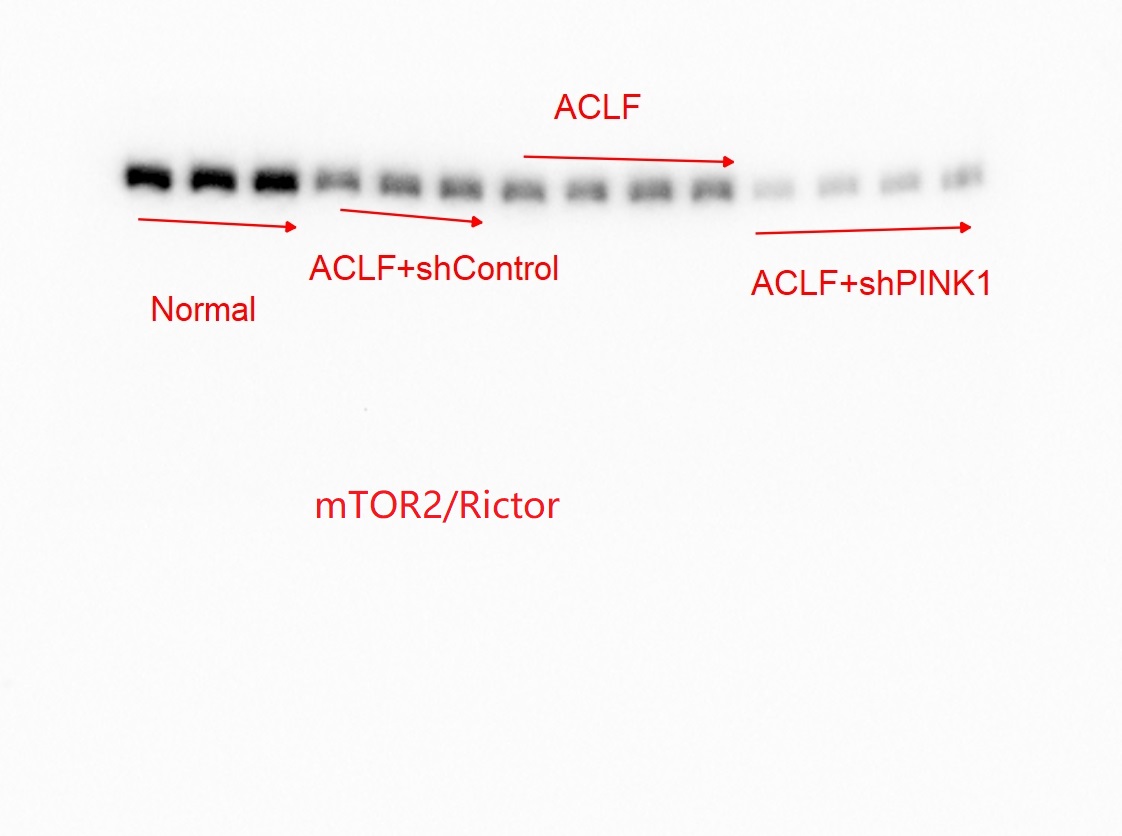

Supplement: Supplementary file 53 — original western blots [file 41420_2022_1021_MOESM53_ESM.jpg]

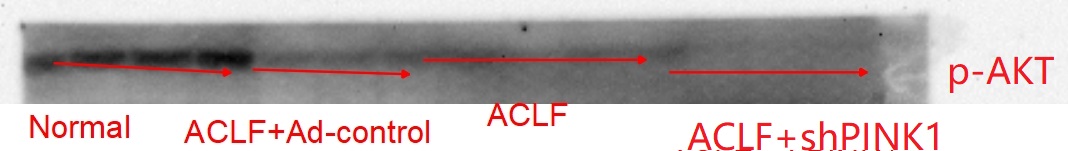

Supplement: Supplementary file 54 — original western blots [file 41420_2022_1021_MOESM54_ESM.jpg]

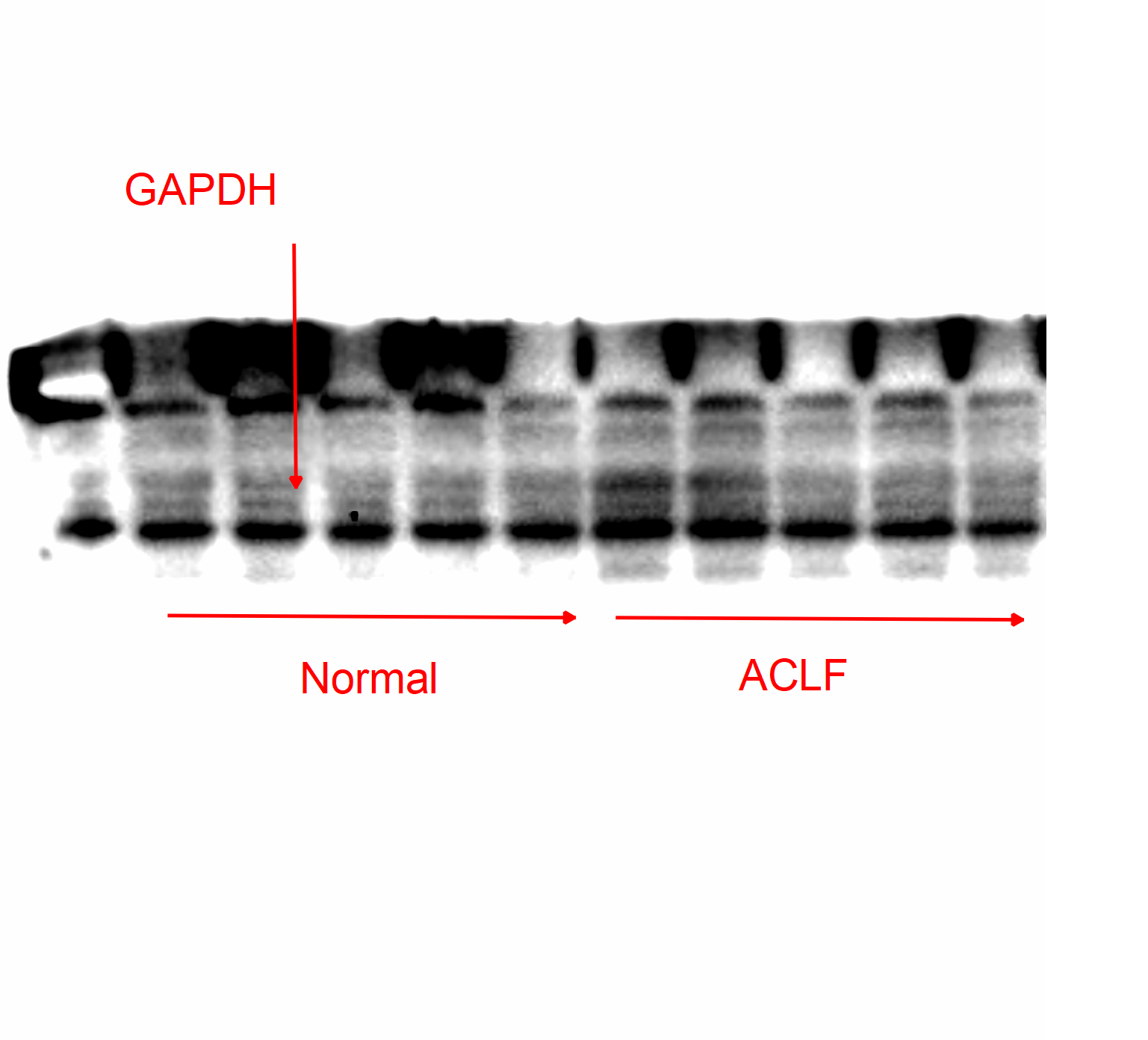

Supplement: Supplementary file 55 — original western blots [file 41420_2022_1021_MOESM55_ESM.tif]

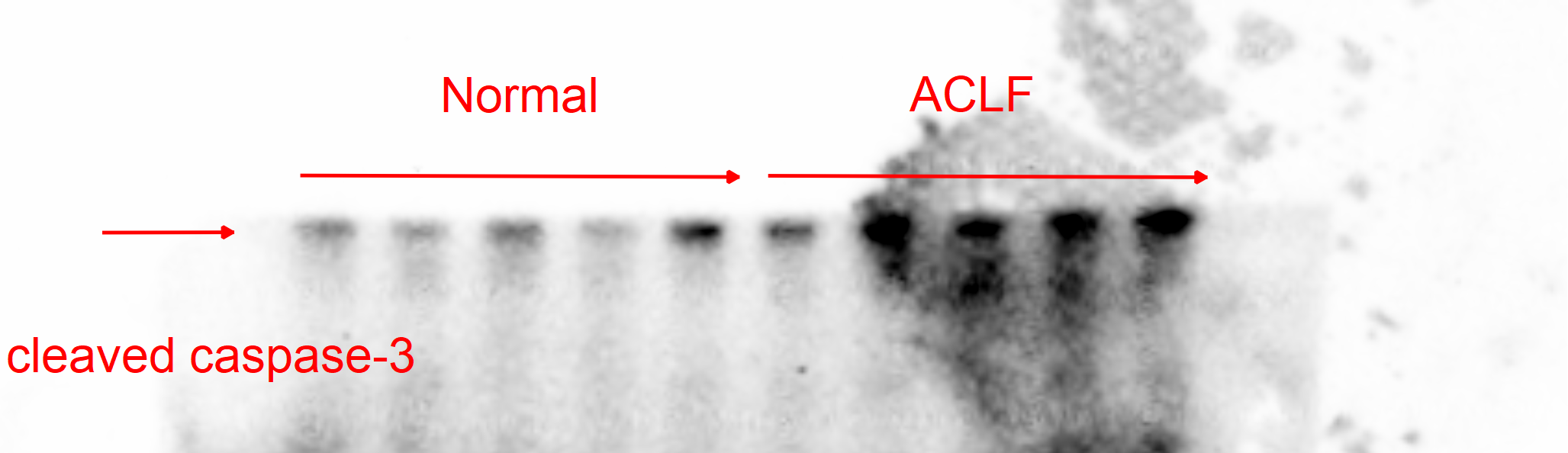

Supplement: Supplementary file 56 — original western blots [file 41420_2022_1021_MOESM56_ESM.tif]

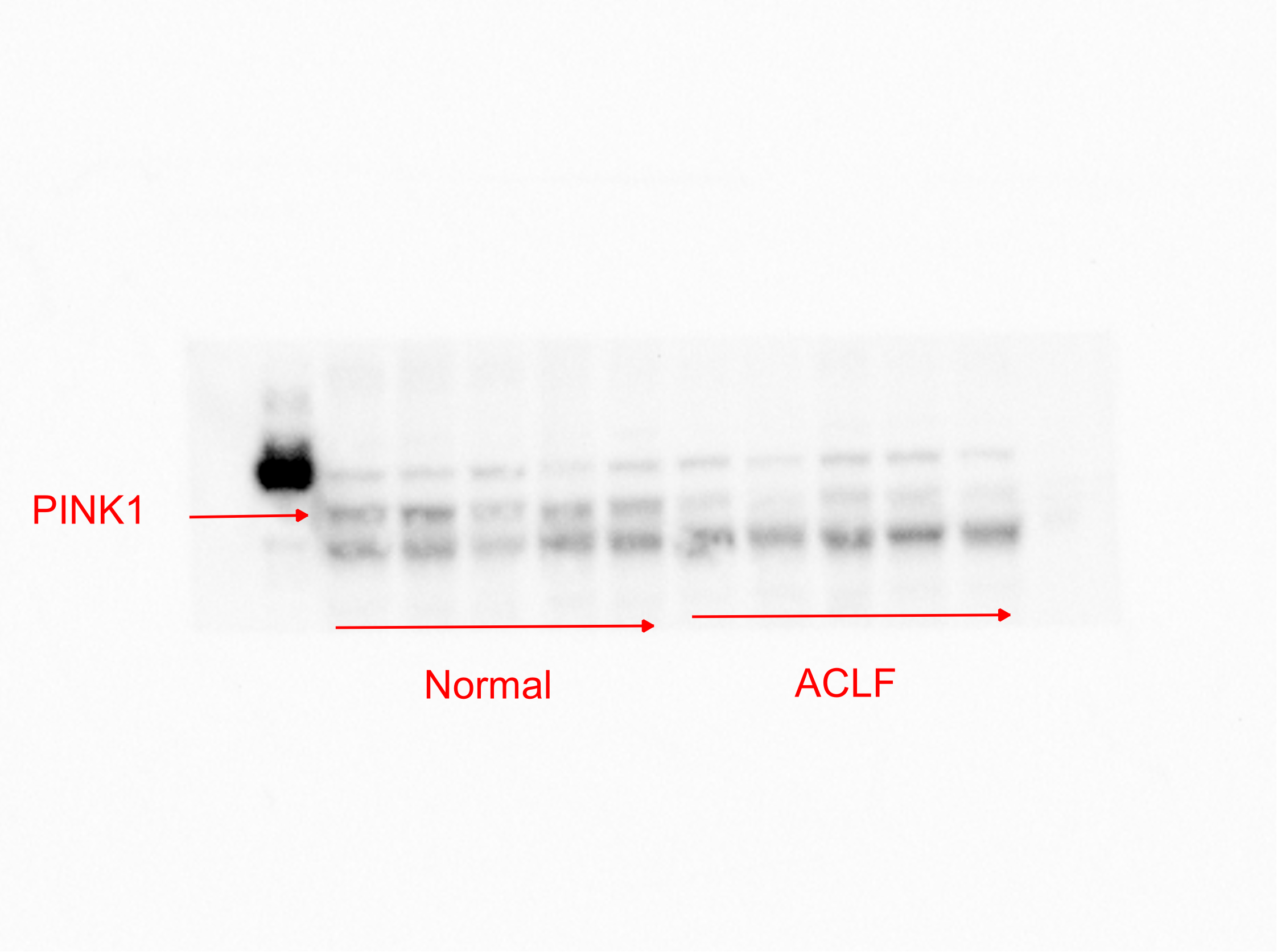

Supplement: Supplementary file 57 — original western blots [file 41420_2022_1021_MOESM57_ESM.tif]

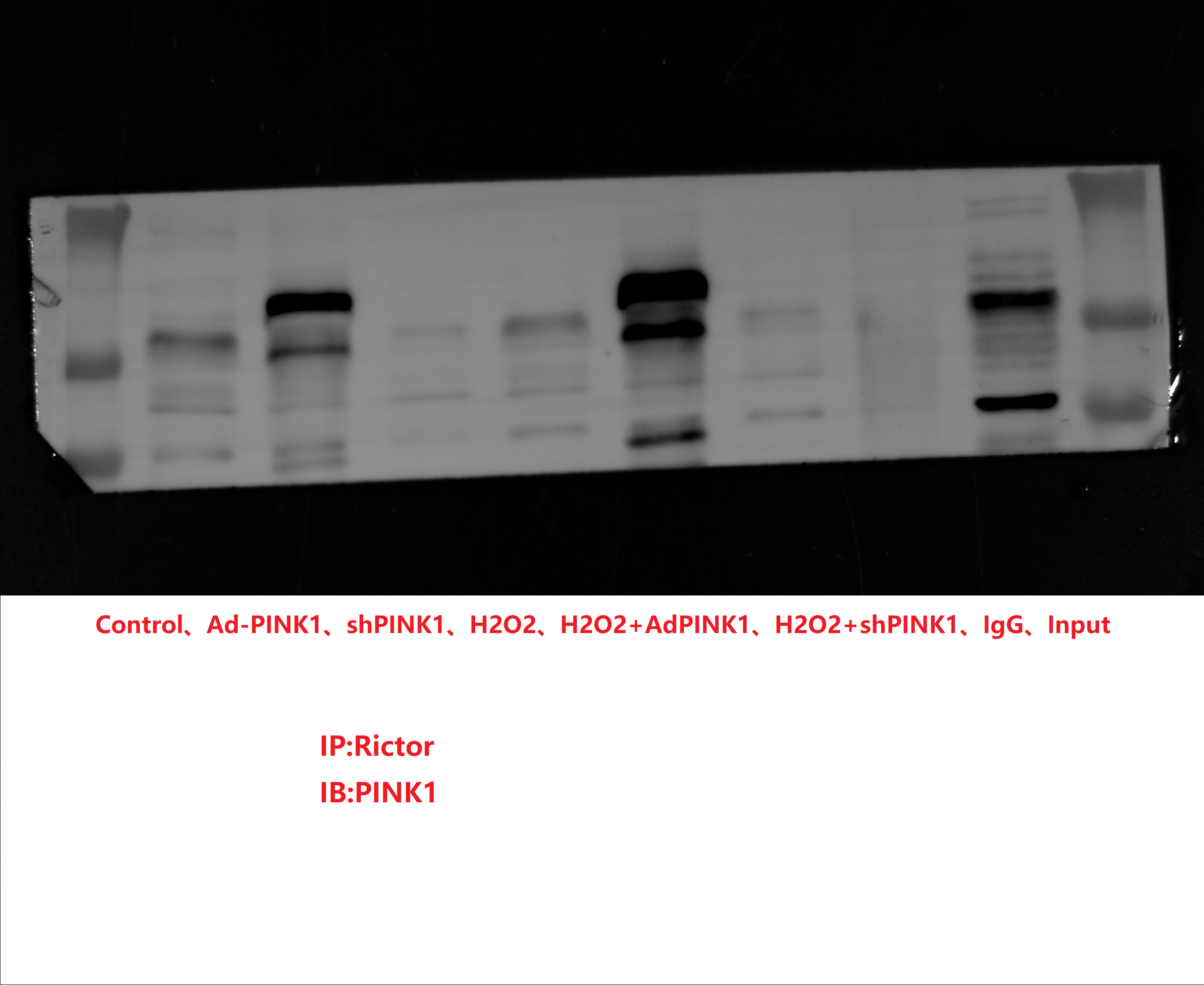

Supplement: Supplementary file 58 — original western blots [file 41420_2022_1021_MOESM58_ESM.tif]

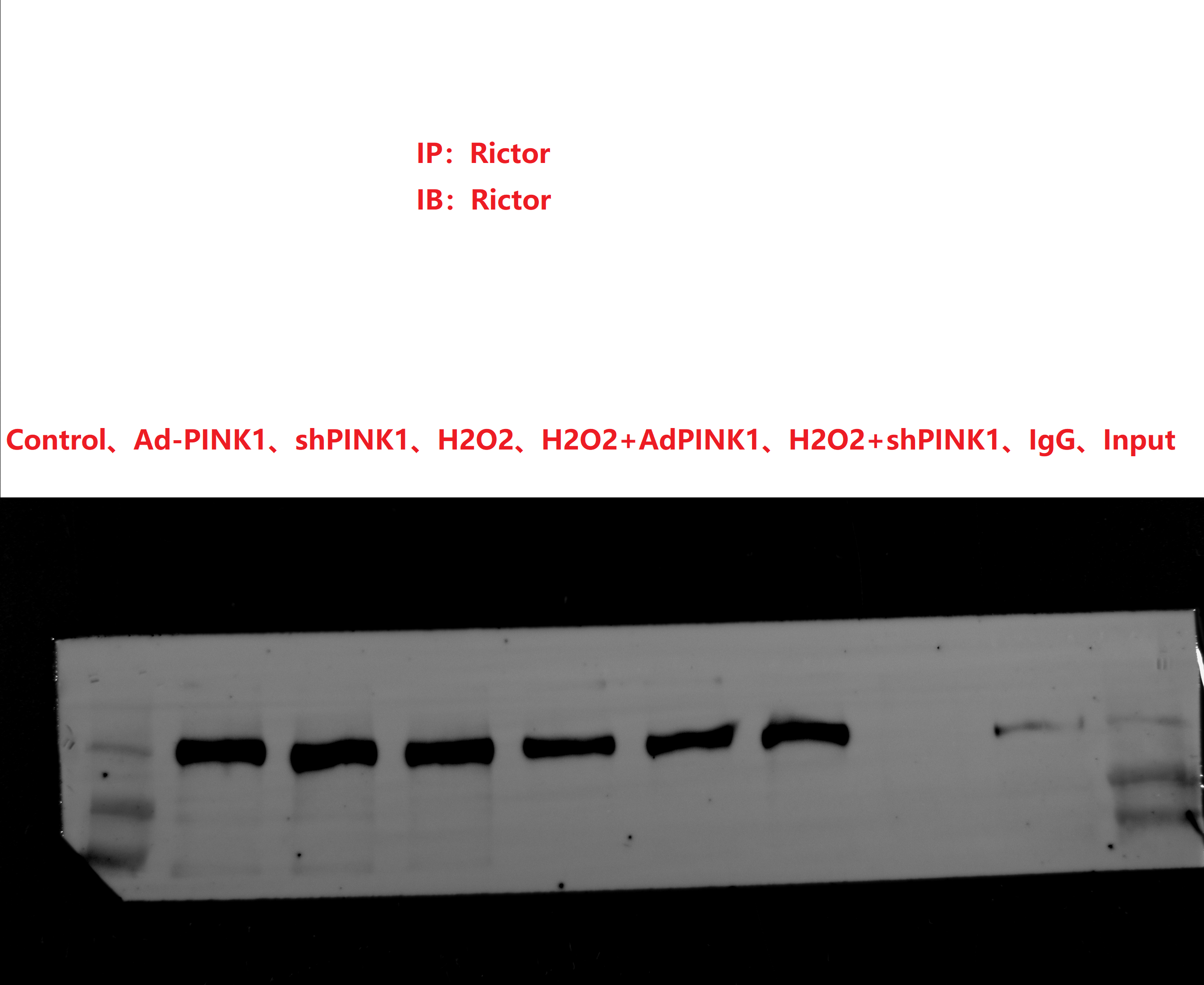

Supplement: Supplementary file 59 — original western blots [file 41420_2022_1021_MOESM59_ESM.tif]

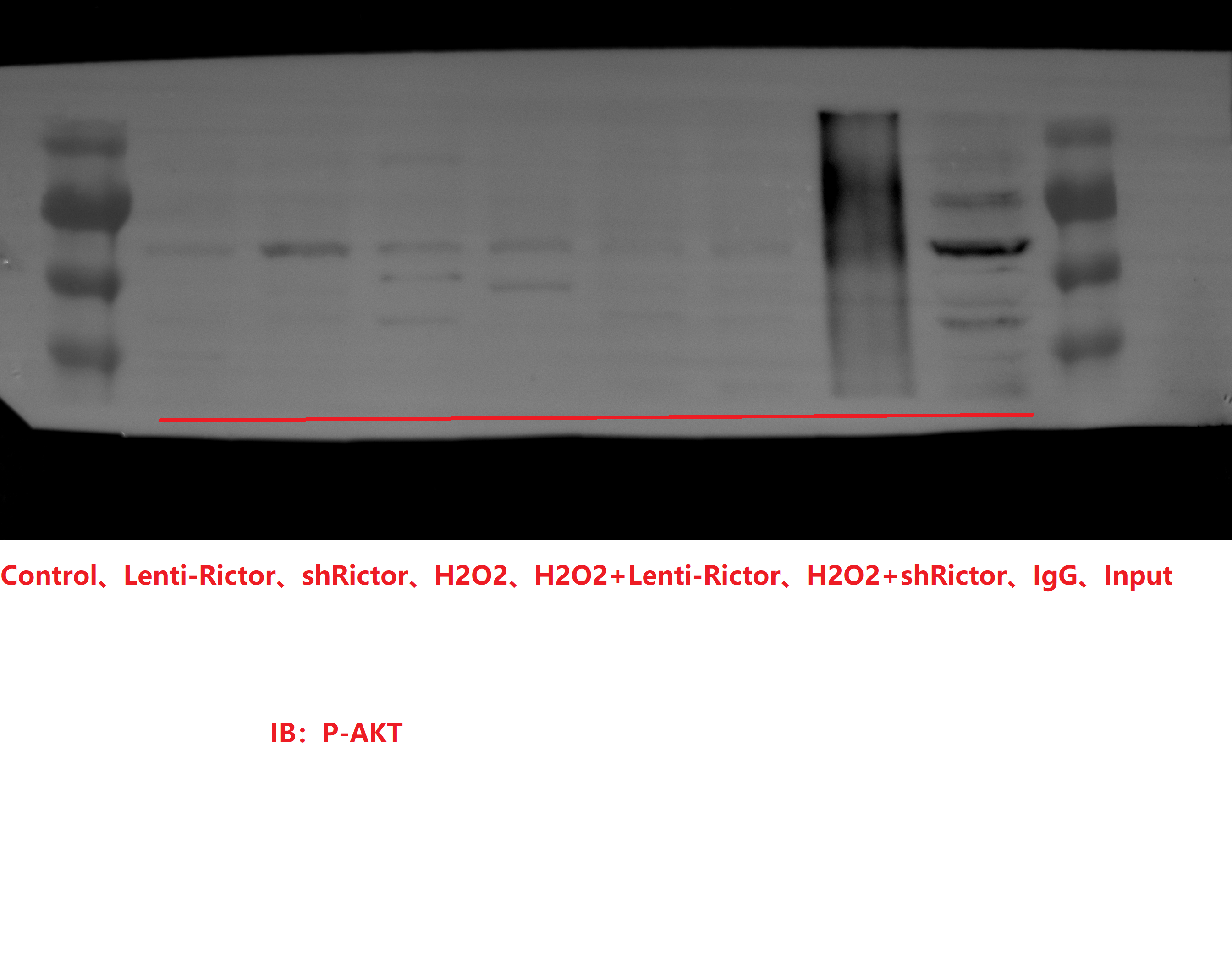

Supplement: Supplementary file 60 — original western blots [file 41420_2022_1021_MOESM60_ESM.tif]

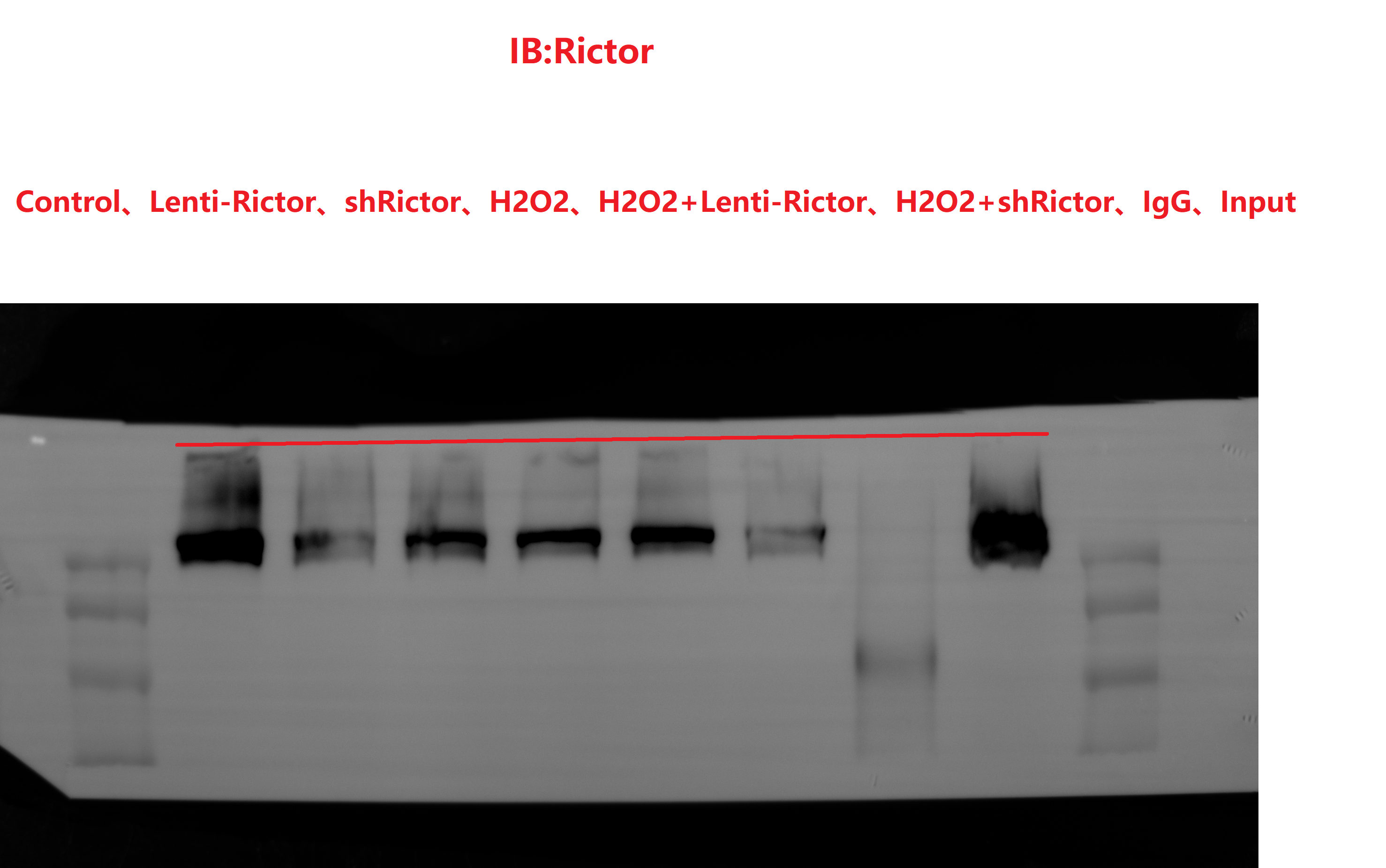

Supplement: Supplementary file 61 — original western blots [file 41420_2022_1021_MOESM61_ESM.tif]

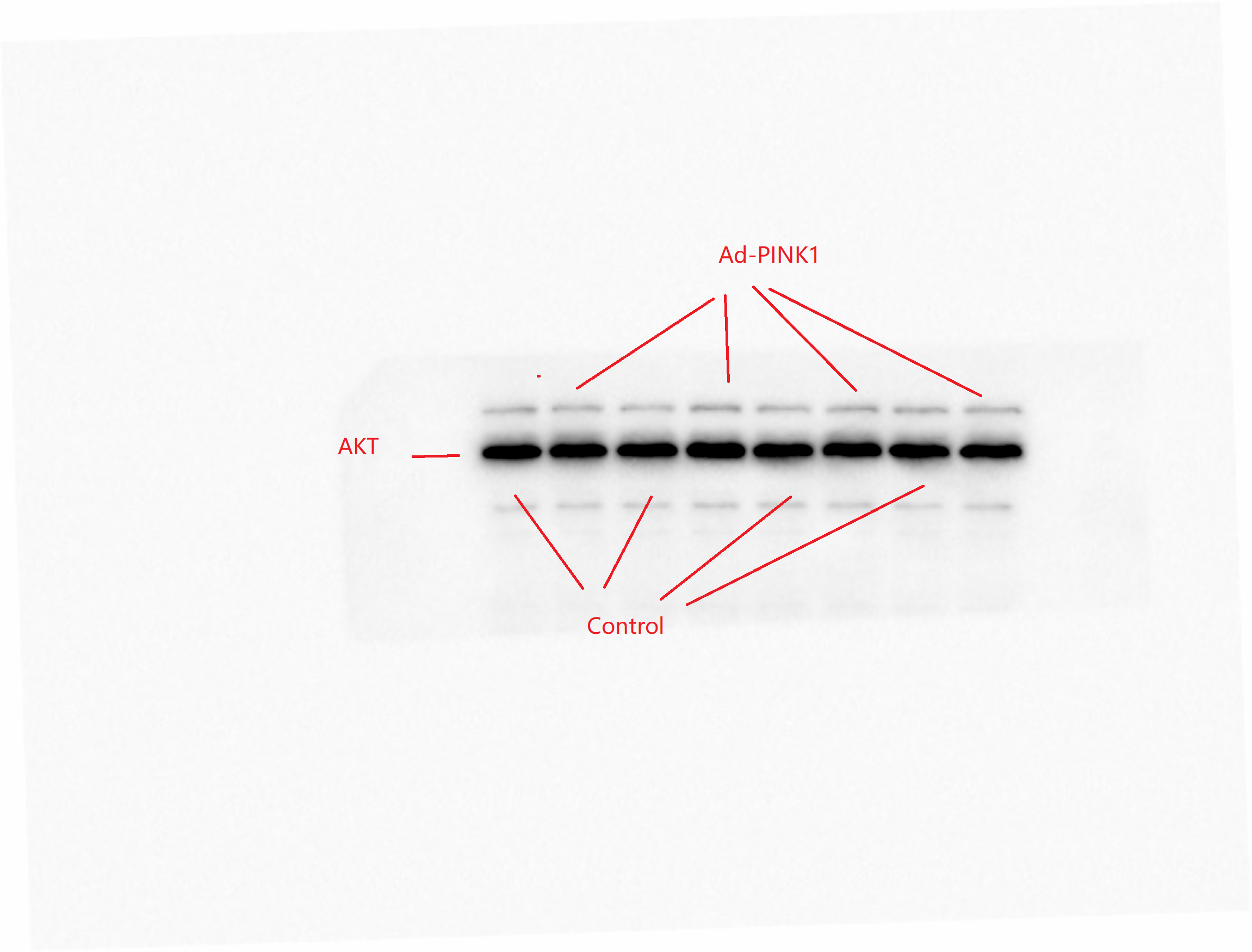

Supplement: Supplementary file 62 — original western blots [file 41420_2022_1021_MOESM62_ESM.tif]

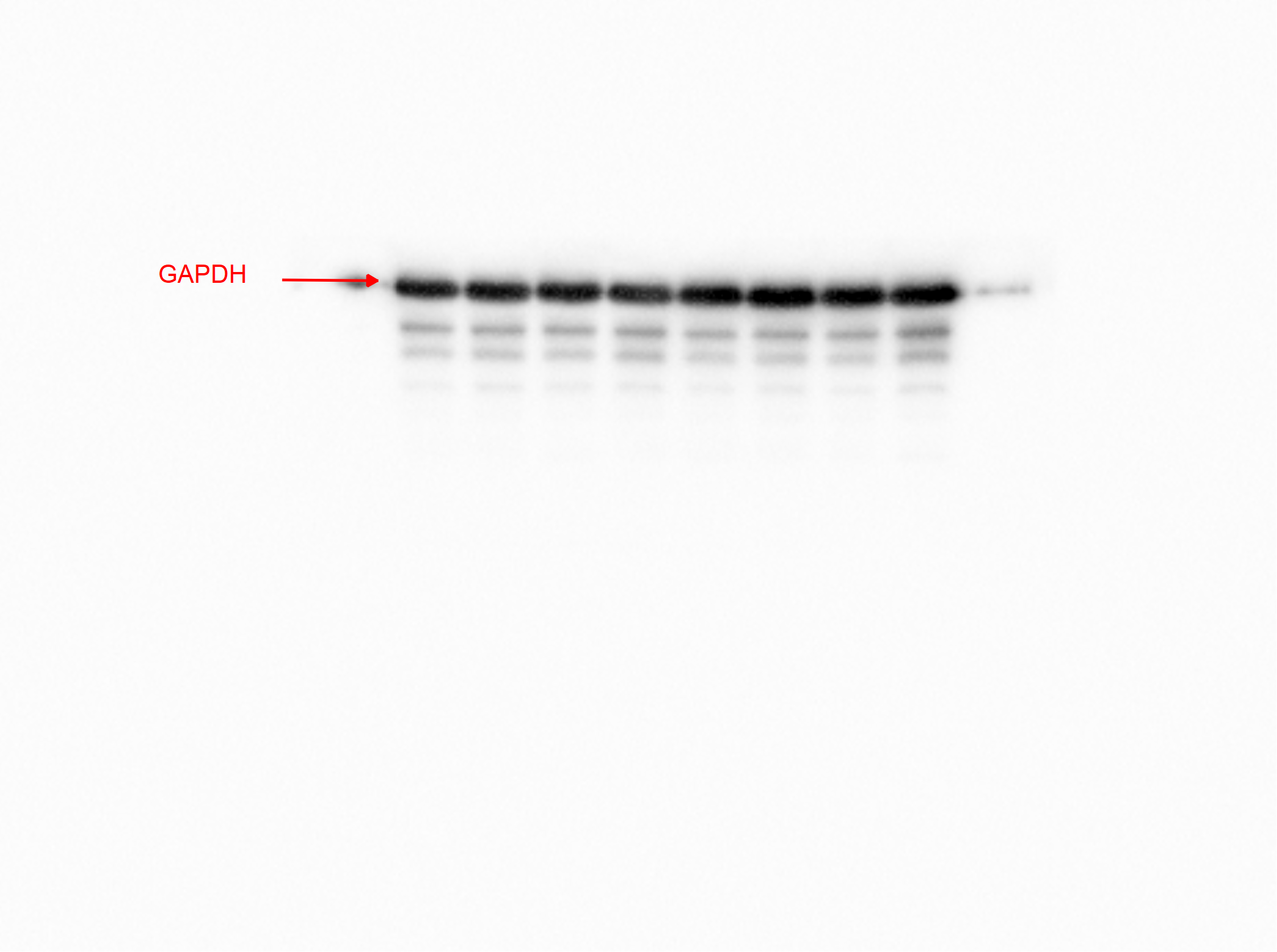

Supplement: Supplementary file 63 — original western blots [file 41420_2022_1021_MOESM63_ESM.tif]

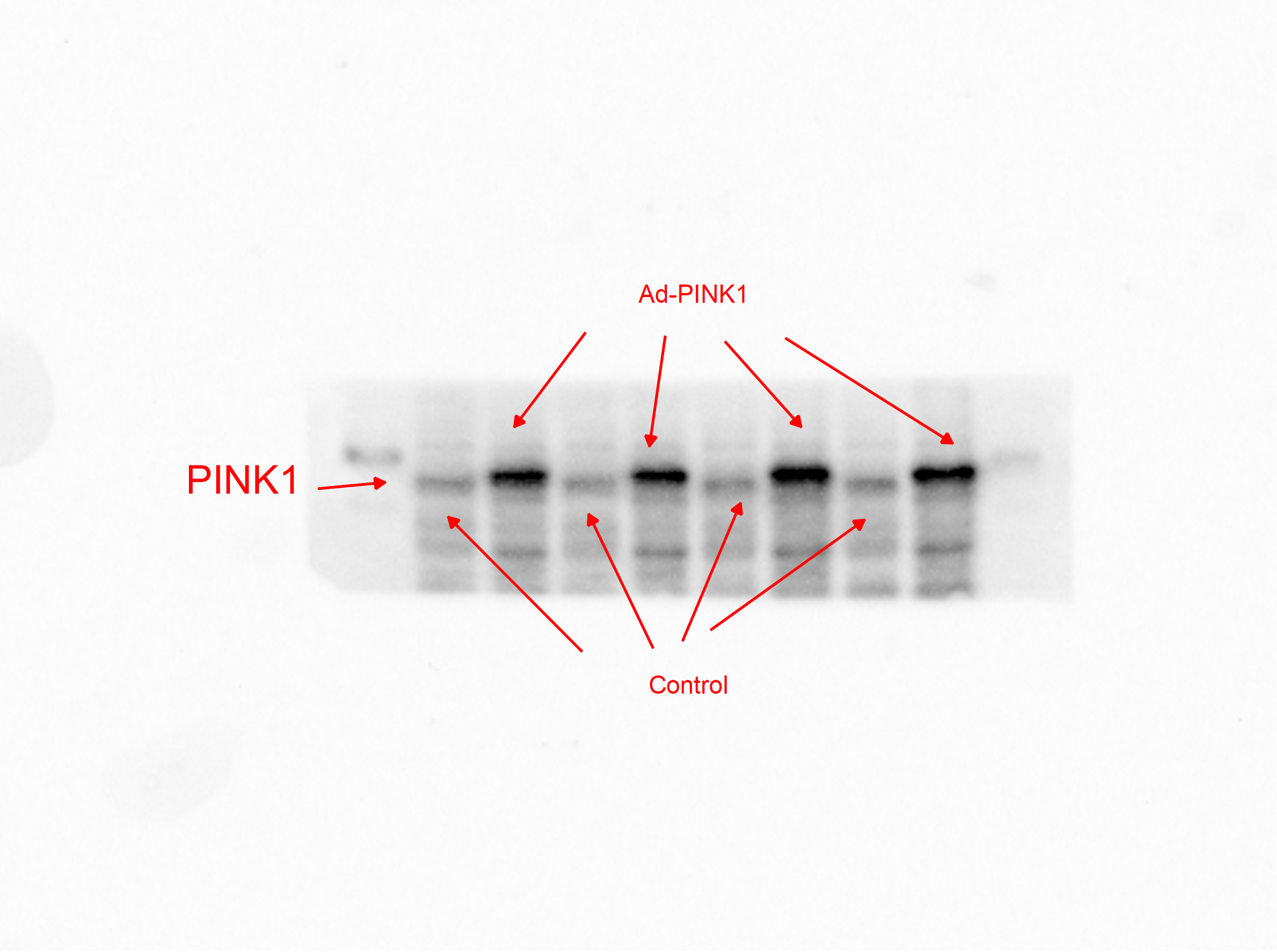

Supplement: Supplementary file 64 — original western blots [file 41420_2022_1021_MOESM64_ESM.tif]

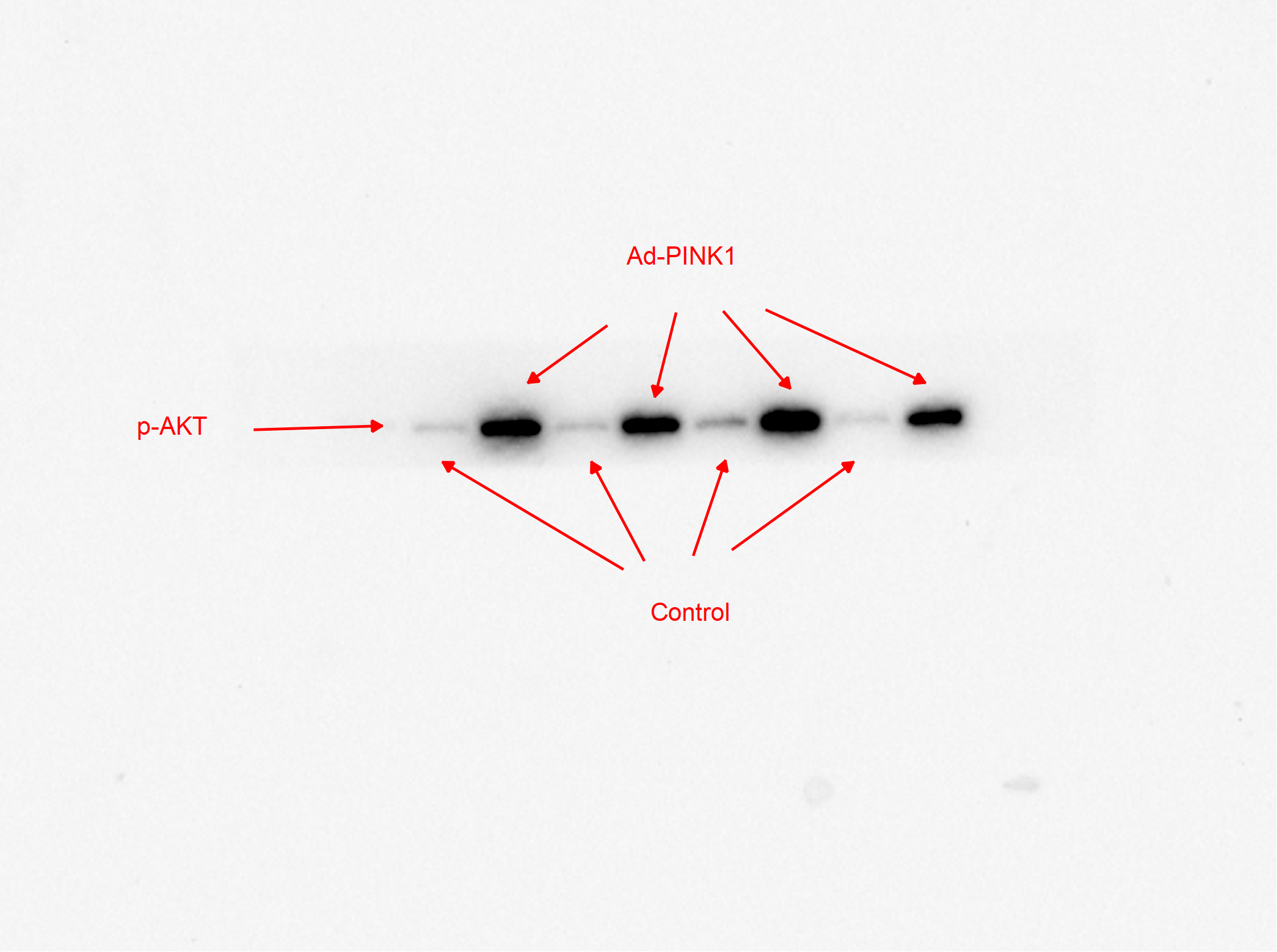

Supplement: Supplementary file 65 — original western blots [file 41420_2022_1021_MOESM65_ESM.tif]

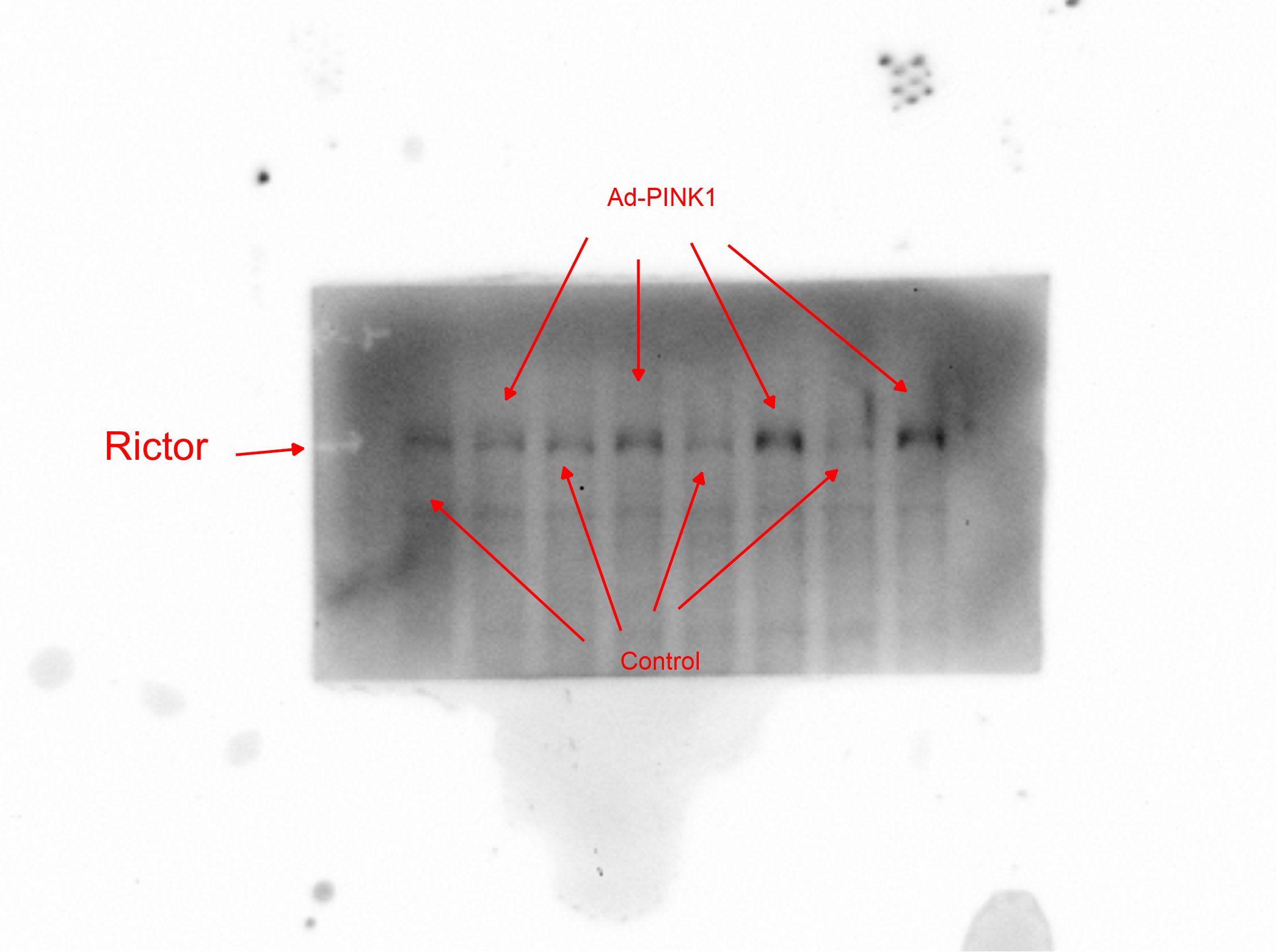

Supplement: Supplementary file 66 — original western blots [file 41420_2022_1021_MOESM66_ESM.tif]

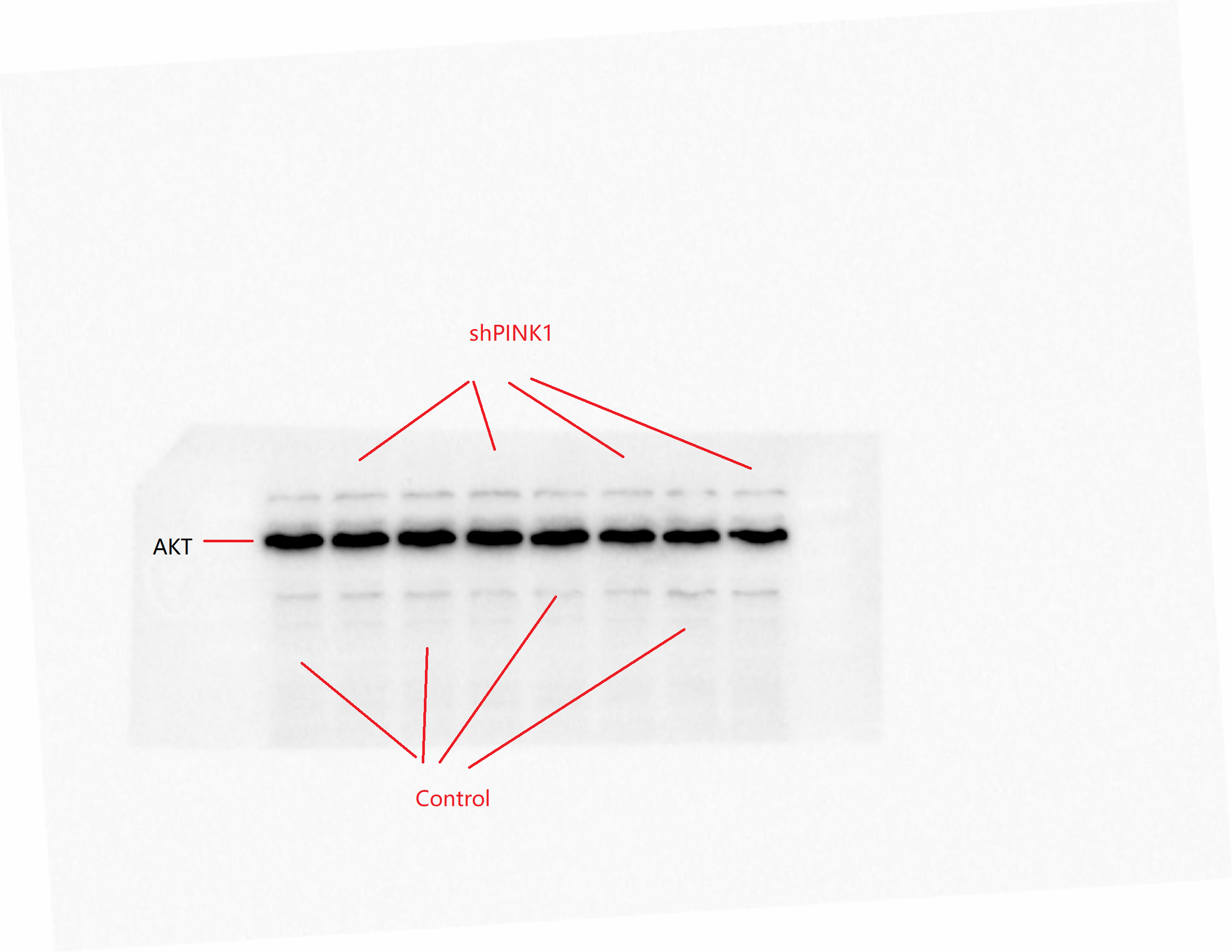

Supplement: Supplementary file 67 — original western blots [file 41420_2022_1021_MOESM67_ESM.tif]

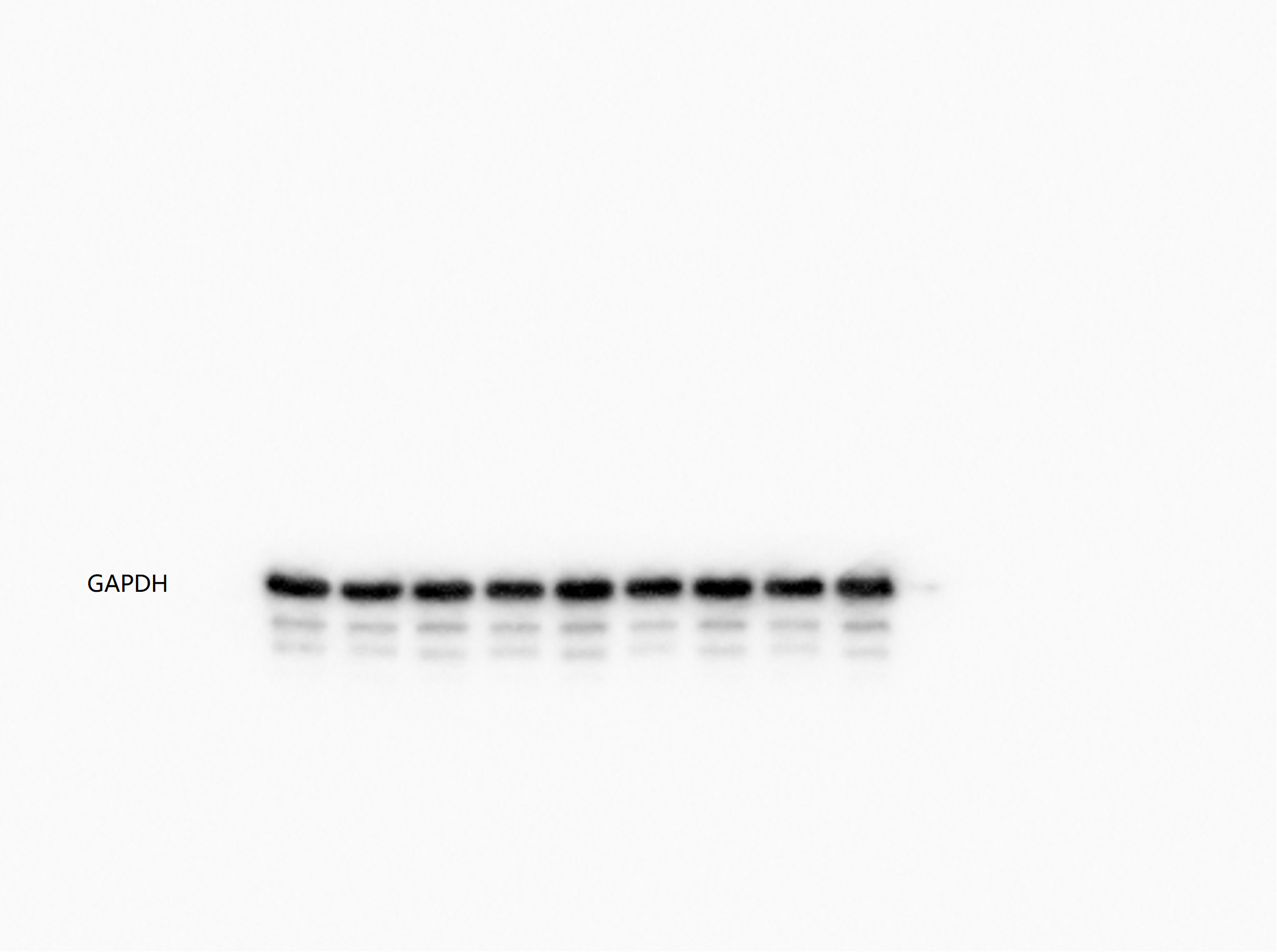

Supplement: Supplementary file 68 — original western blots [file 41420_2022_1021_MOESM68_ESM.tif]

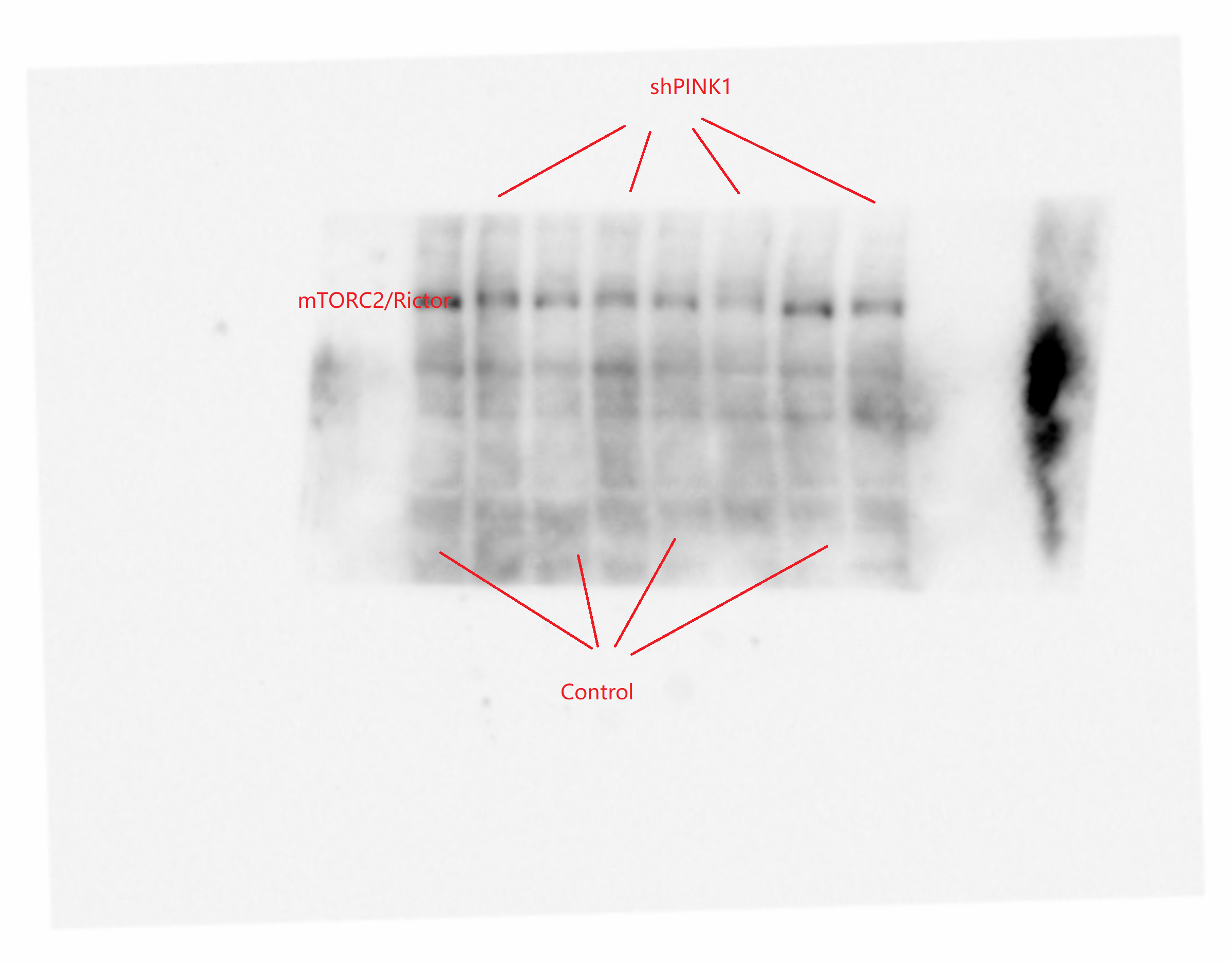

Supplement: Supplementary file 69 — original western blots [file 41420_2022_1021_MOESM69_ESM.tif]

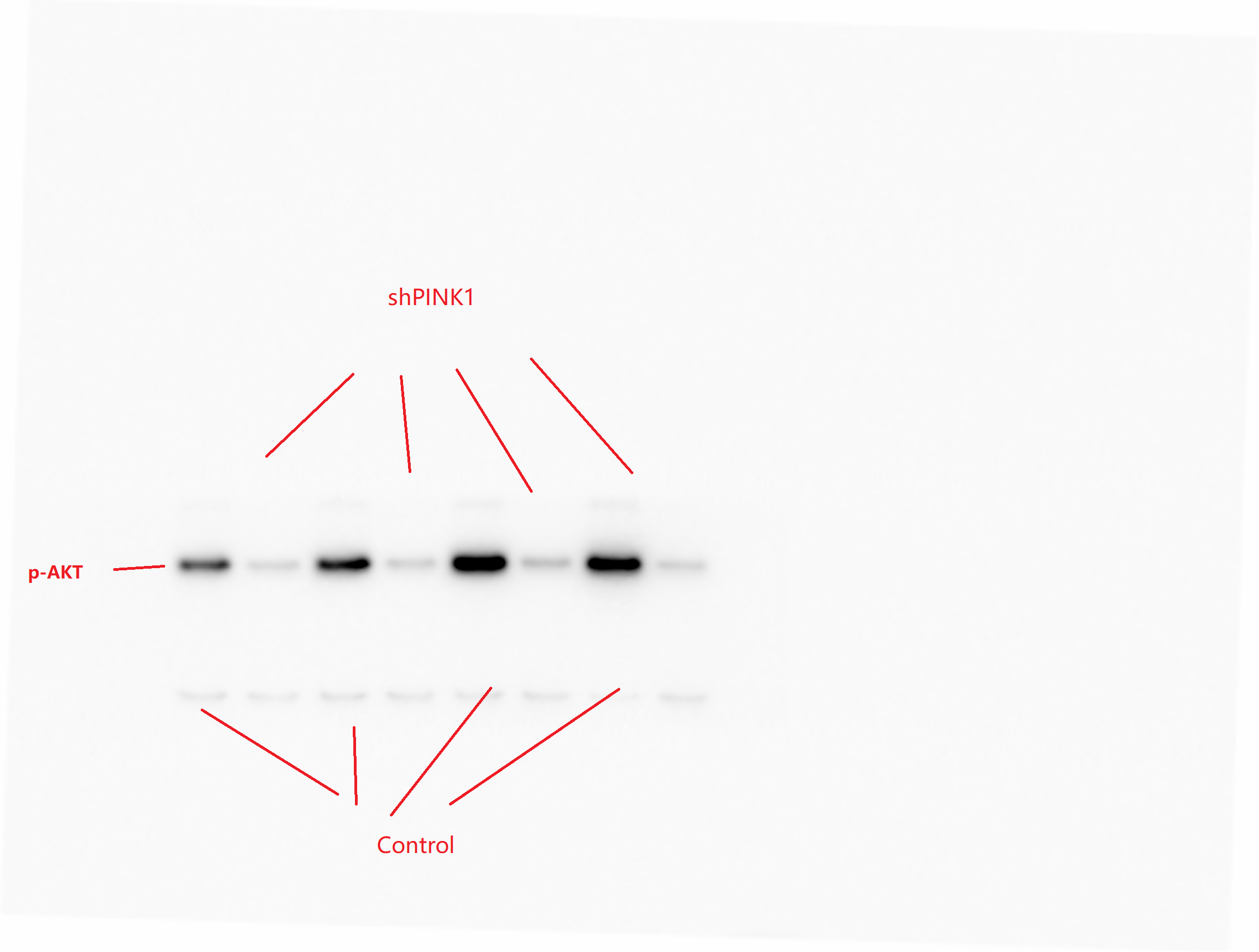

Supplement: Supplementary file 70 — original western blots [file 41420_2022_1021_MOESM70_ESM.tif]

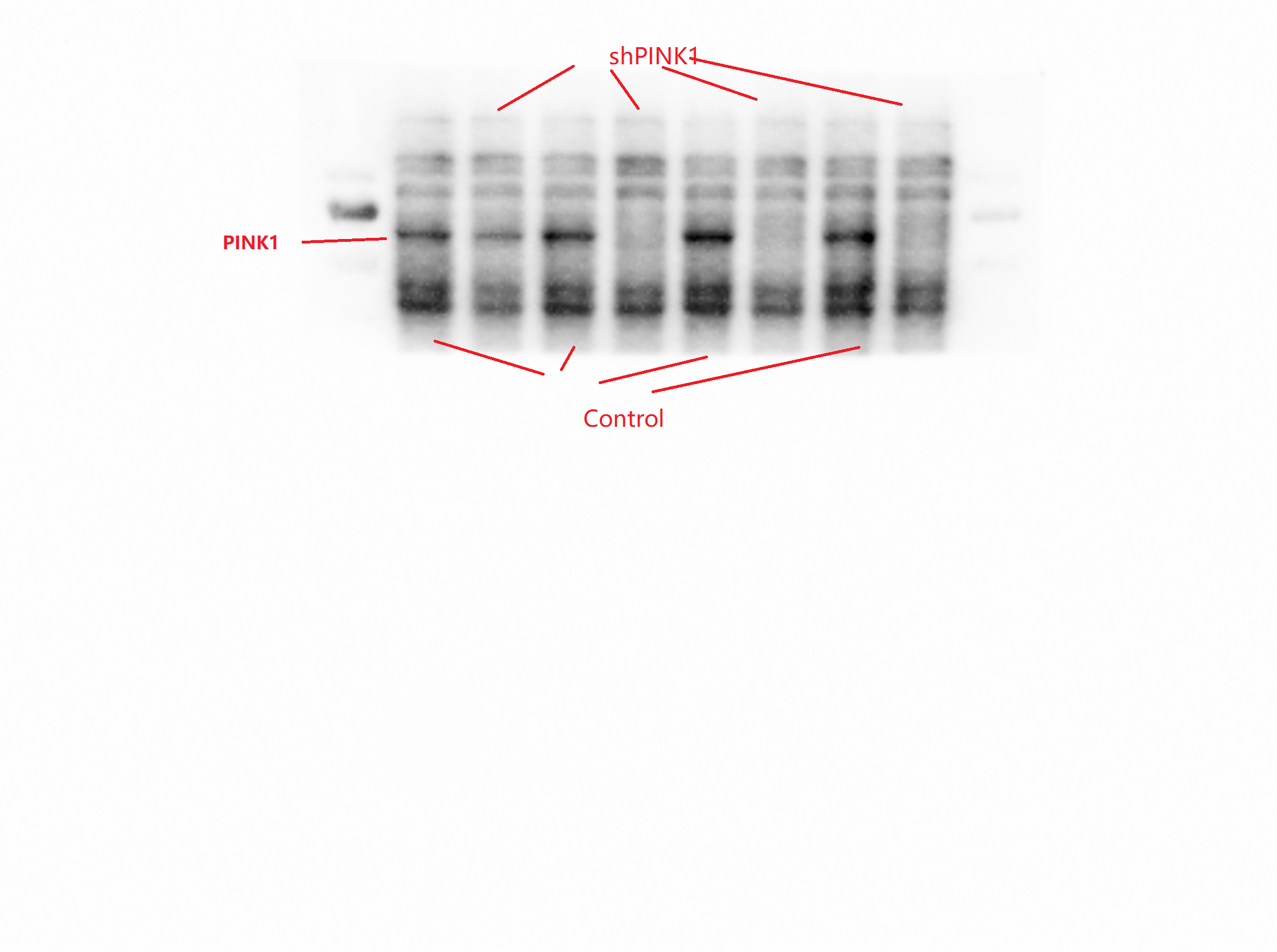

Supplement: Supplementary file 71 — original western blots [file 41420_2022_1021_MOESM71_ESM.tif]

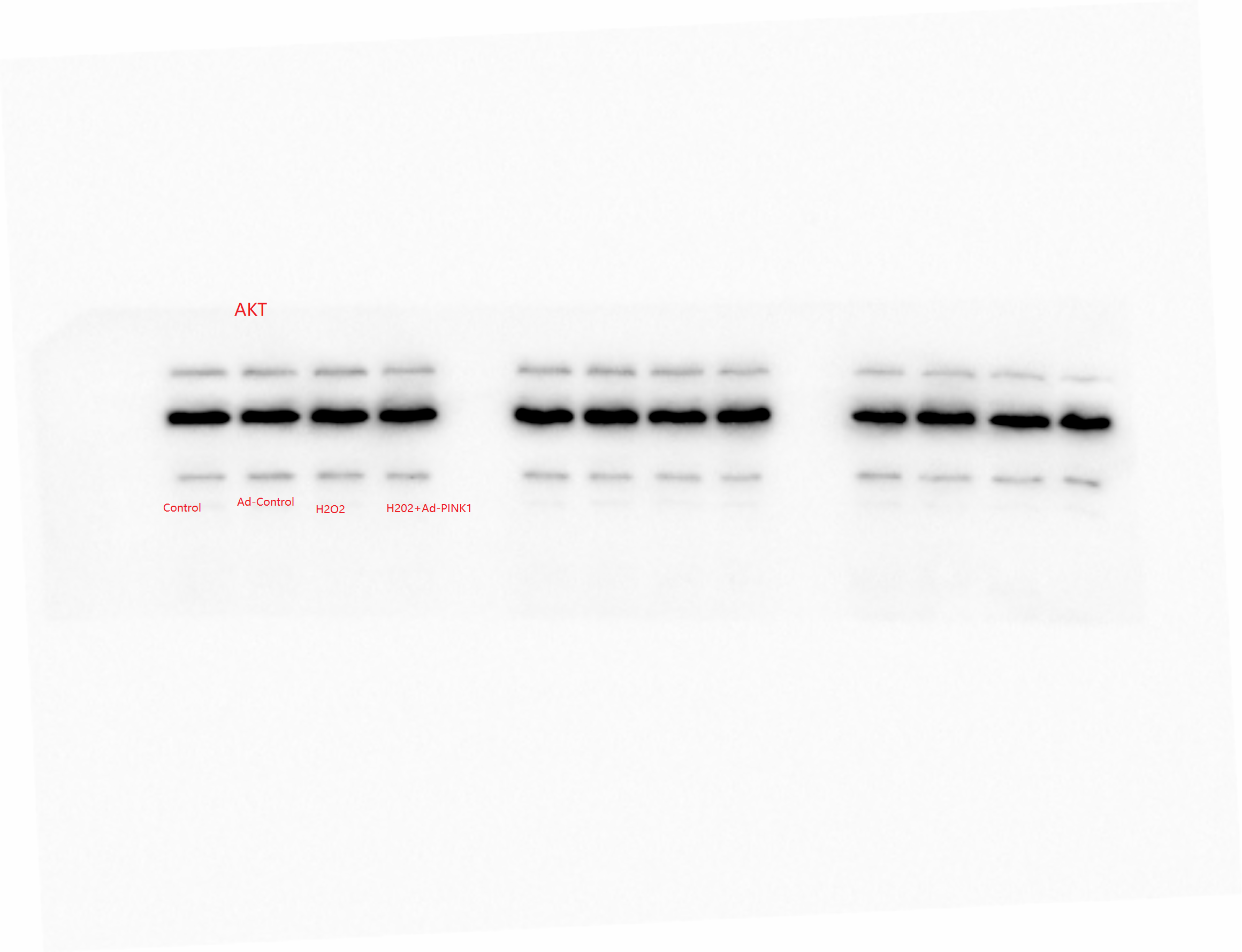

Supplement: Supplementary file 72 — original western blots [file 41420_2022_1021_MOESM72_ESM.tif]

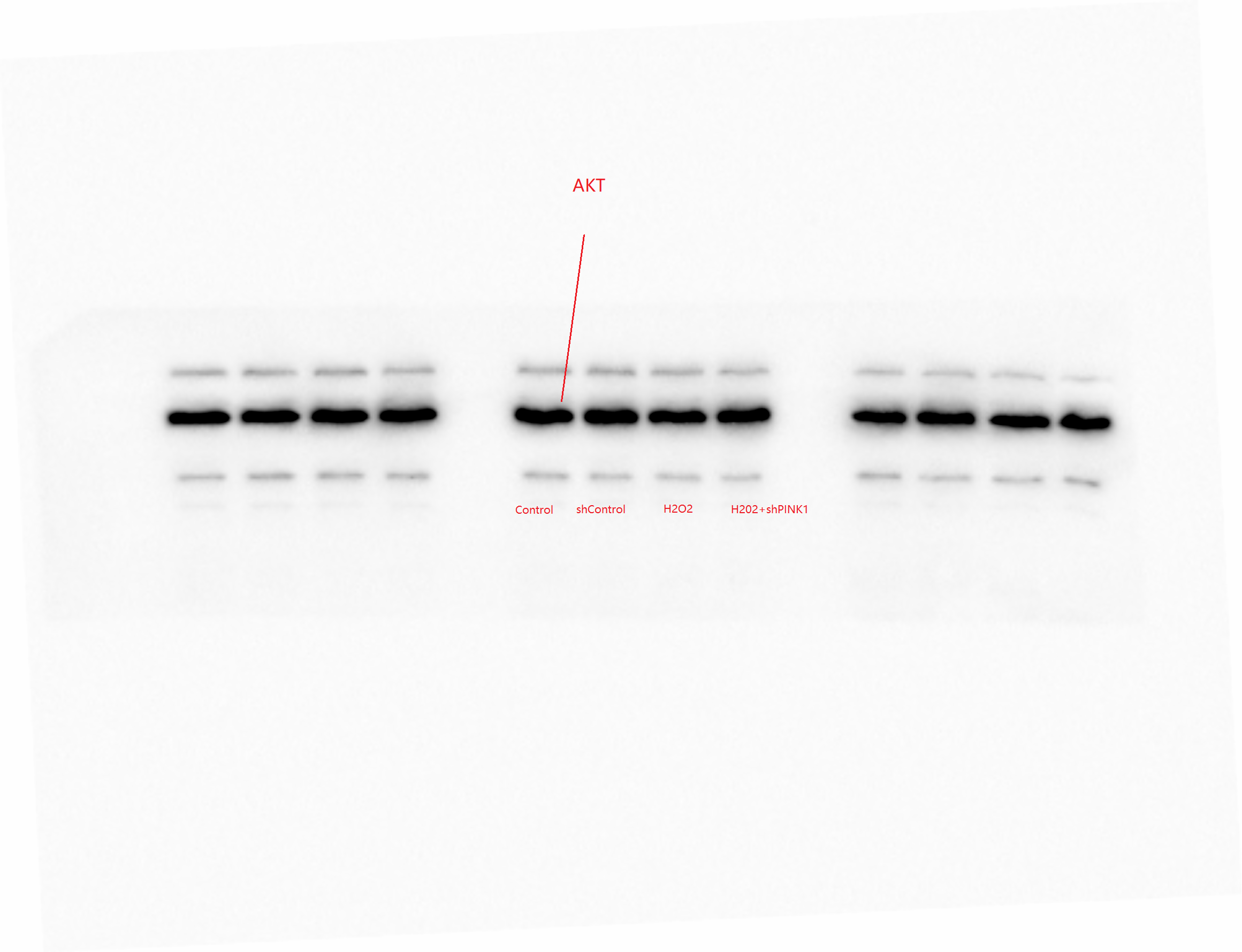

Supplement: Supplementary file 73 — original western blots [file 41420_2022_1021_MOESM73_ESM.tif]

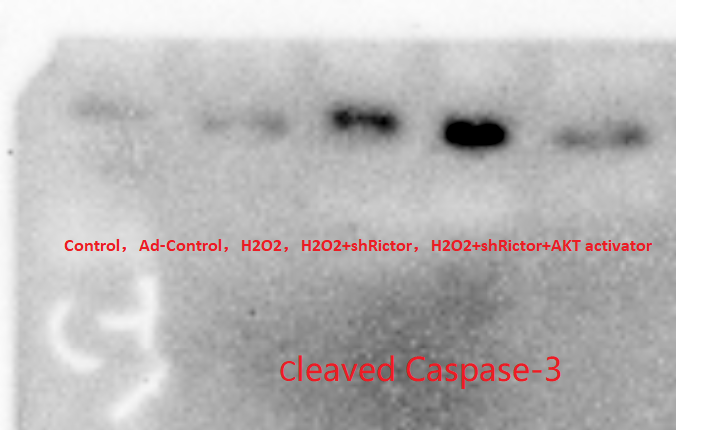

Supplement: Supplementary file 74 — original western blots [file 41420_2022_1021_MOESM74_ESM.tif]

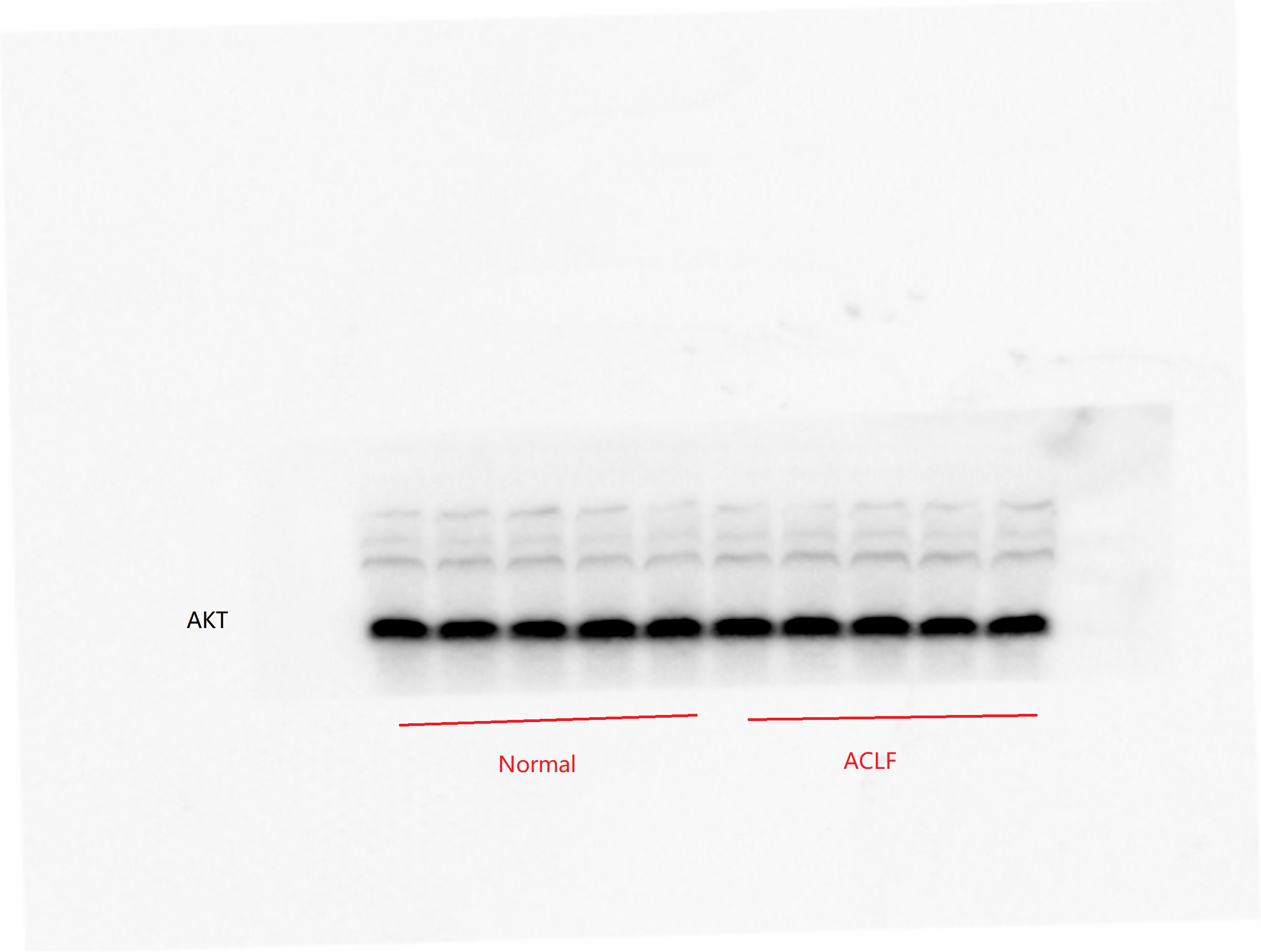

Supplement: Supplementary file 75 — original western blots [file 41420_2022_1021_MOESM75_ESM.tif]

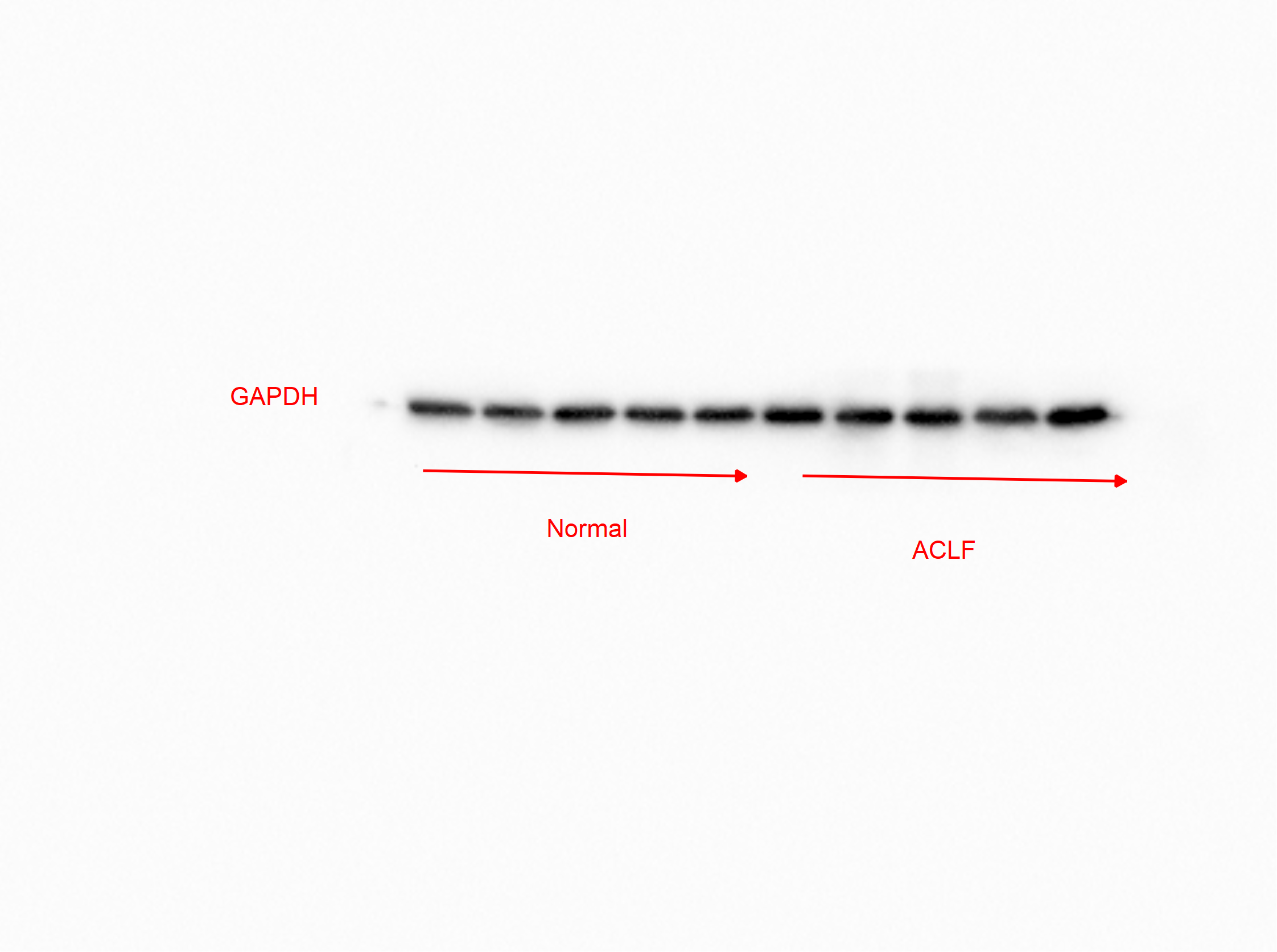

Supplement: Supplementary file 76 — original western blots [file 41420_2022_1021_MOESM76_ESM.tif]

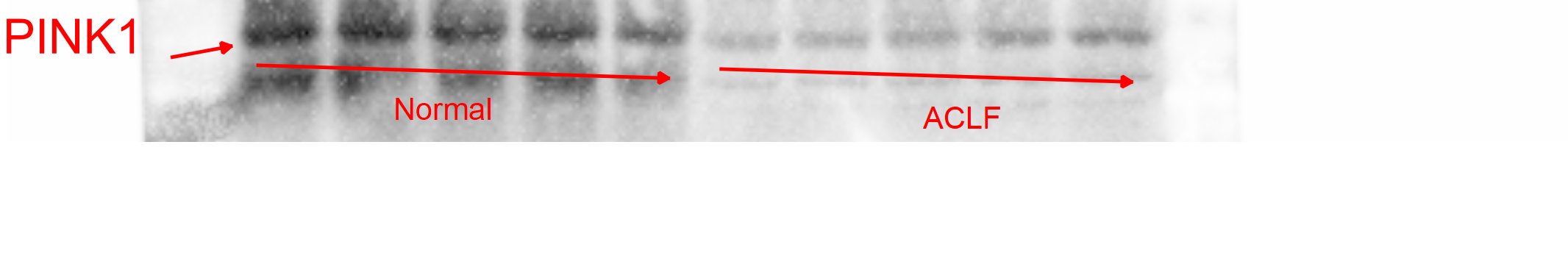

Supplement: Supplementary file 77 — original western blots [file 41420_2022_1021_MOESM77_ESM.tif]

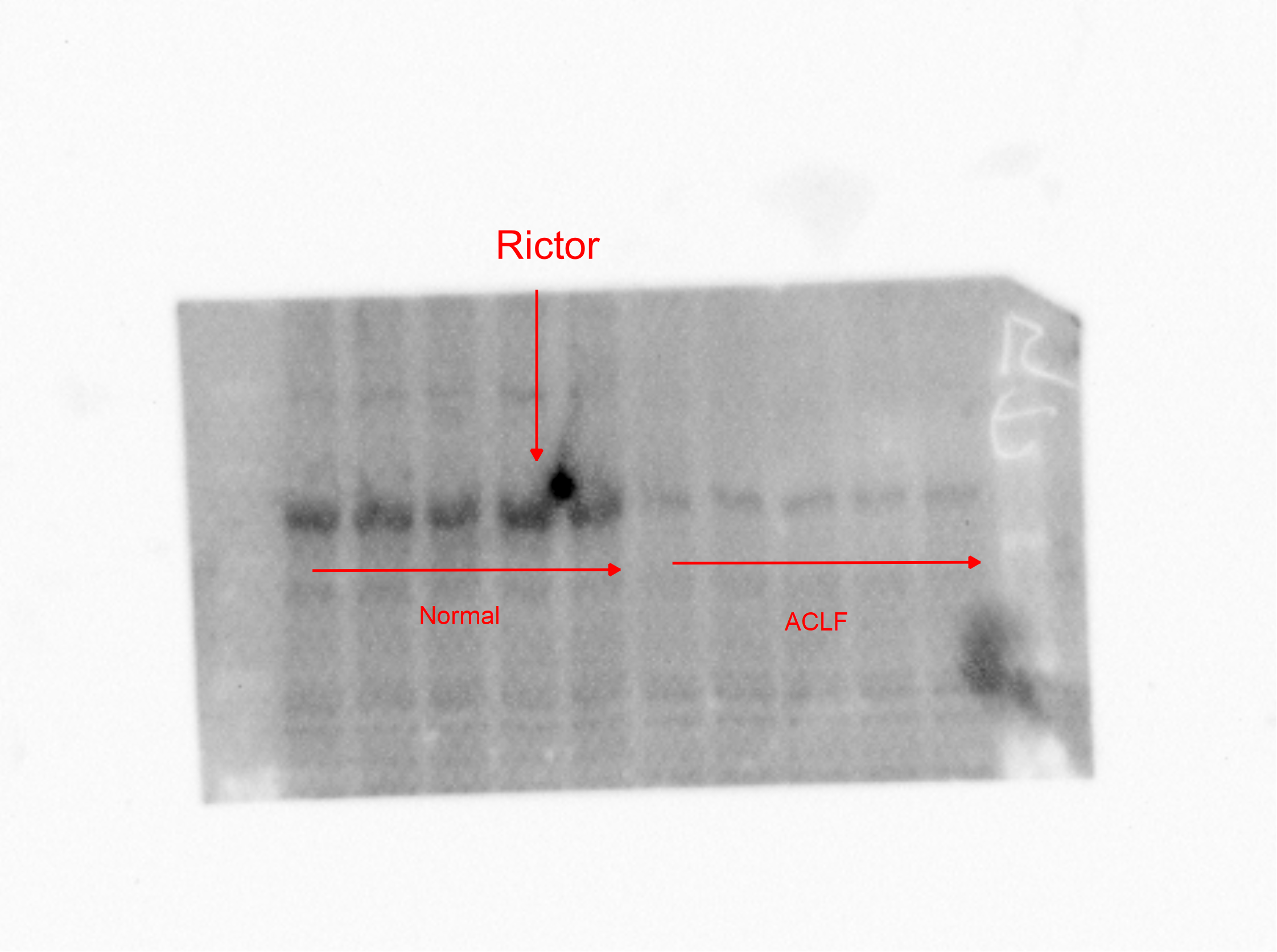

Supplement: Supplementary file 78 — original western blots [file 41420_2022_1021_MOESM78_ESM.tif]

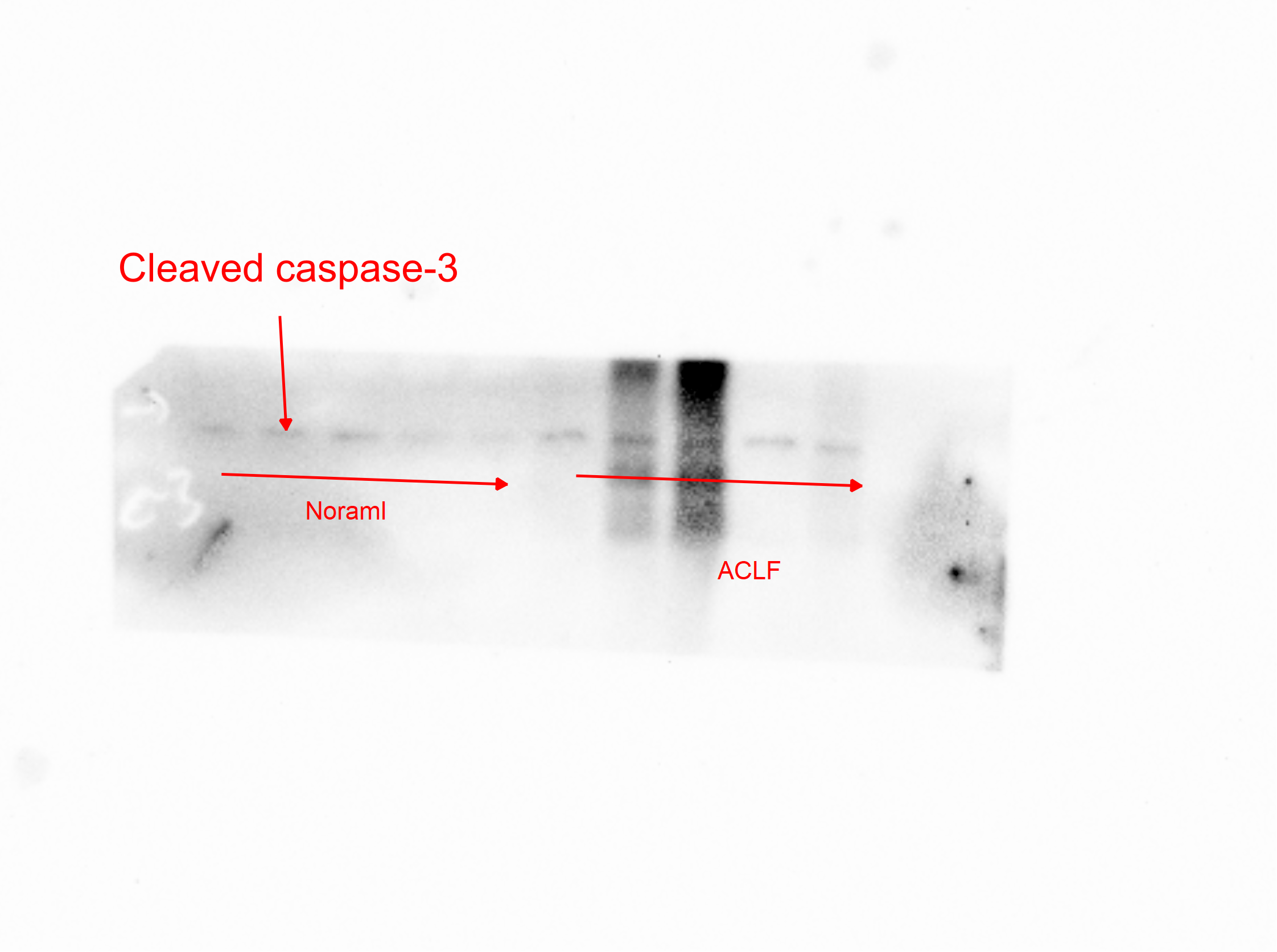

Supplement: Supplementary file 79 — original western blots [file 41420_2022_1021_MOESM79_ESM.tif]

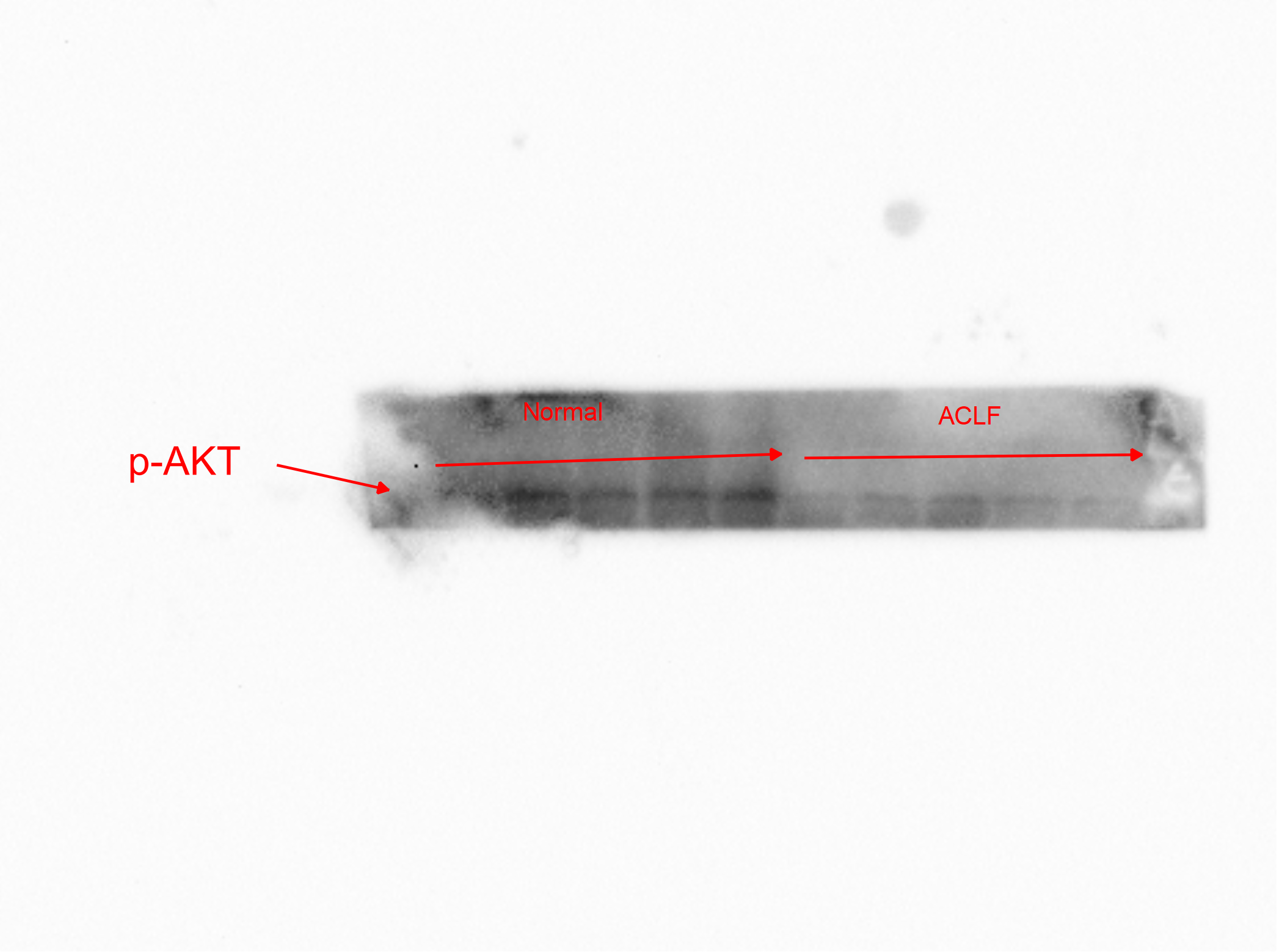

Supplement: Supplementary file 80 — original western blots [file 41420_2022_1021_MOESM80_ESM.tif]

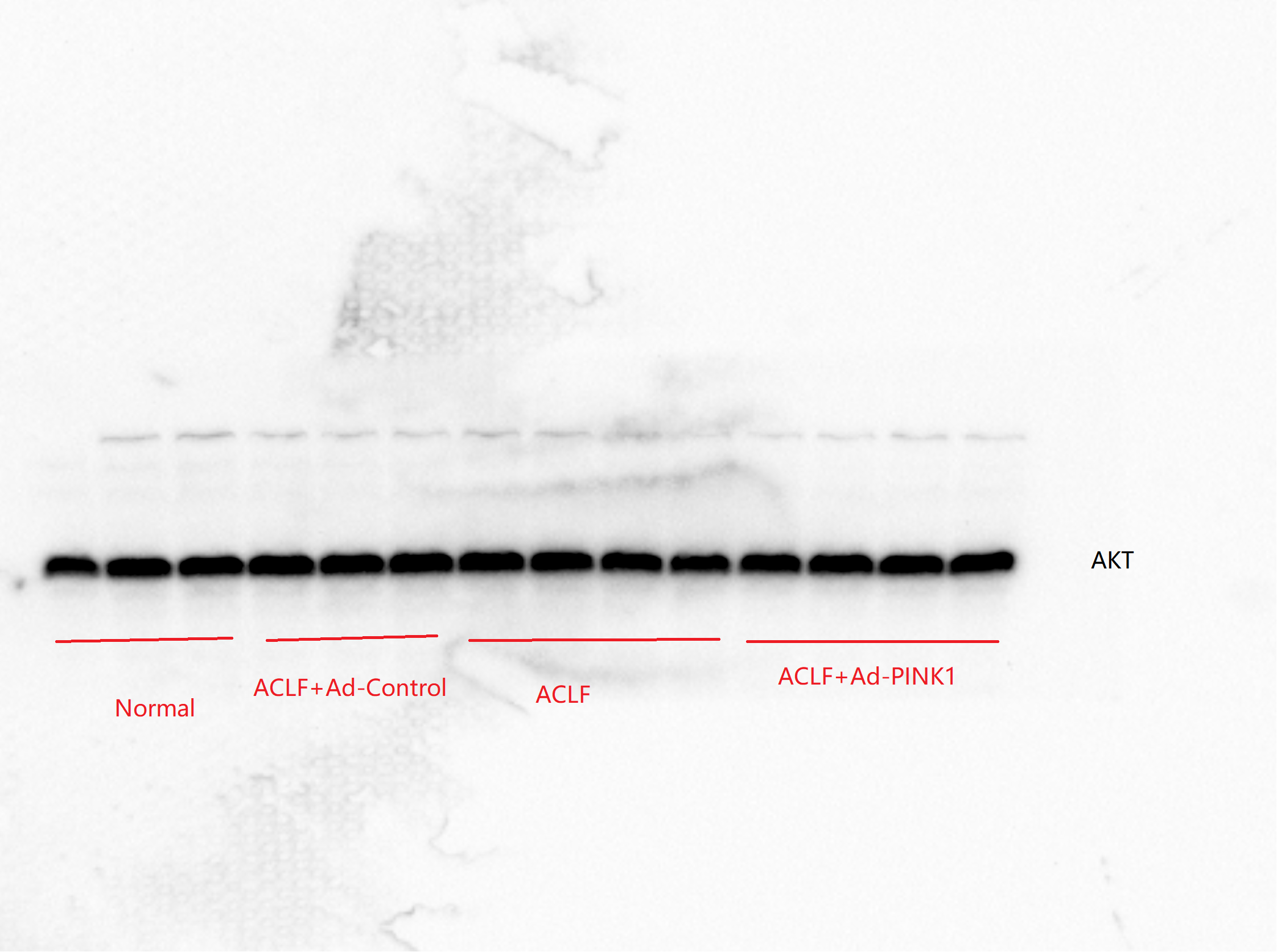

Supplement: Supplementary file 81 — original western blots [file 41420_2022_1021_MOESM81_ESM.tif]

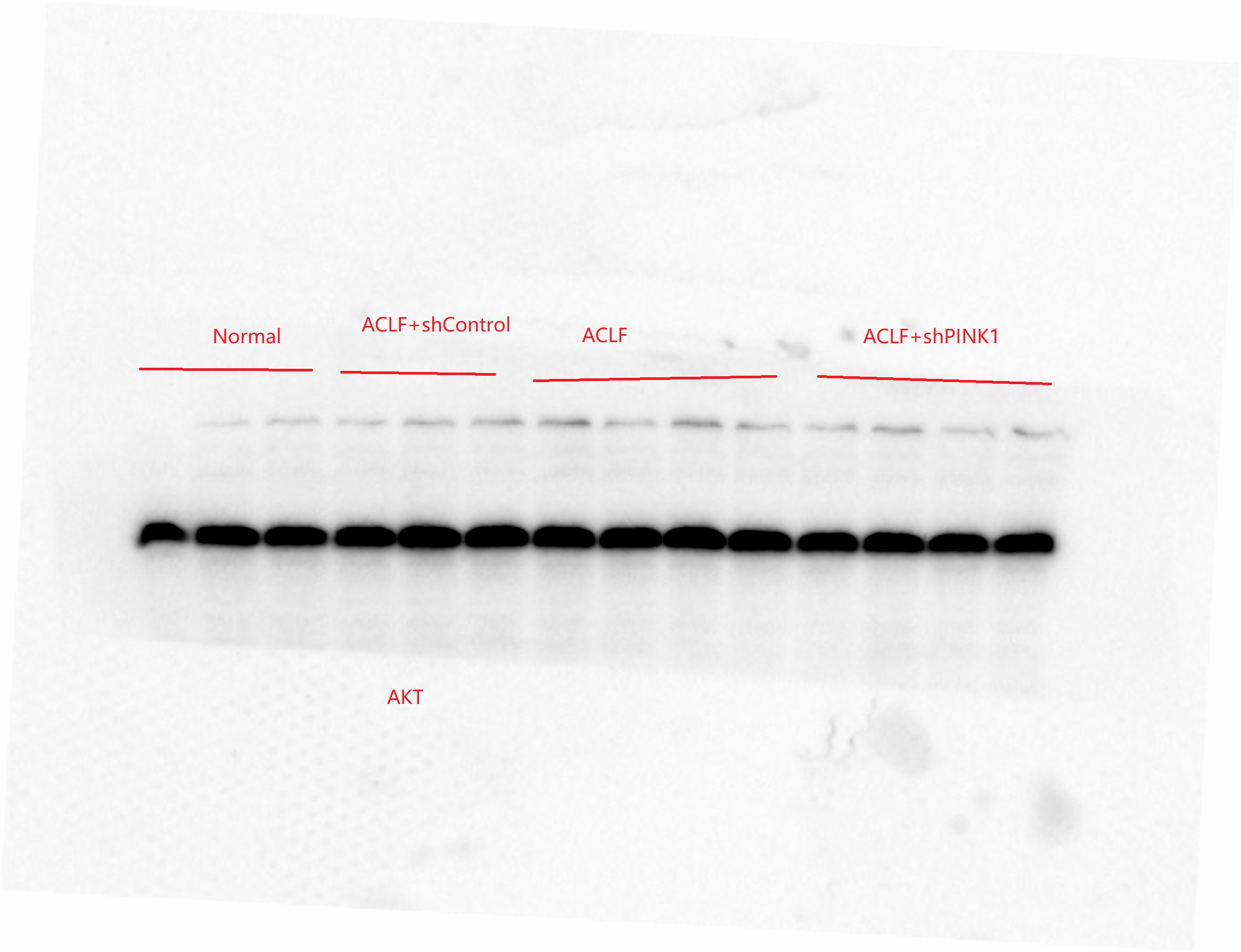

Supplement: Supplementary file 82 — original western blots [file 41420_2022_1021_MOESM82_ESM.tif]

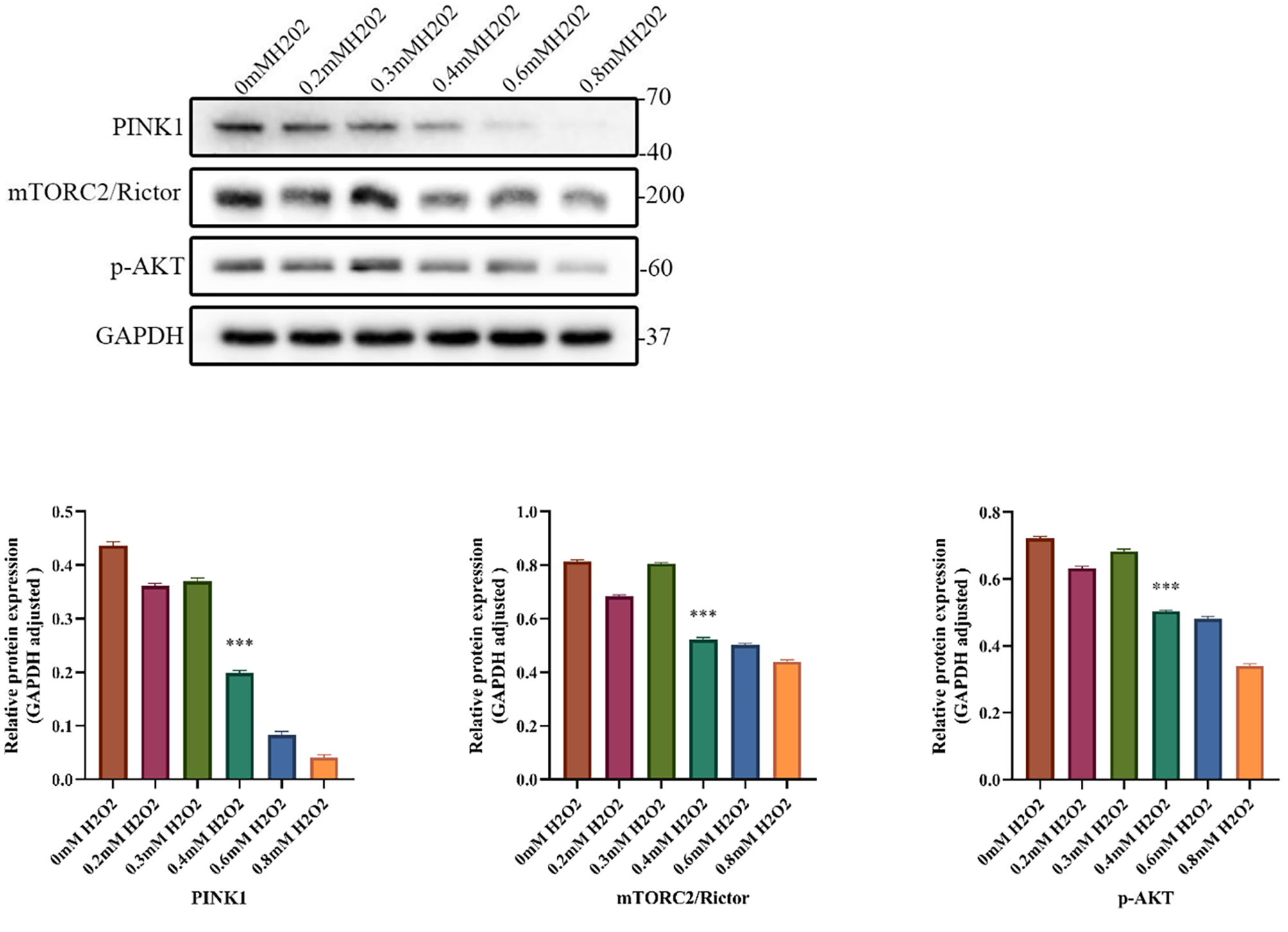

Supplement: Supplementary file 87 — Supplemental figure 1 [file 41420_2022_1021_MOESM87_ESM.tif]

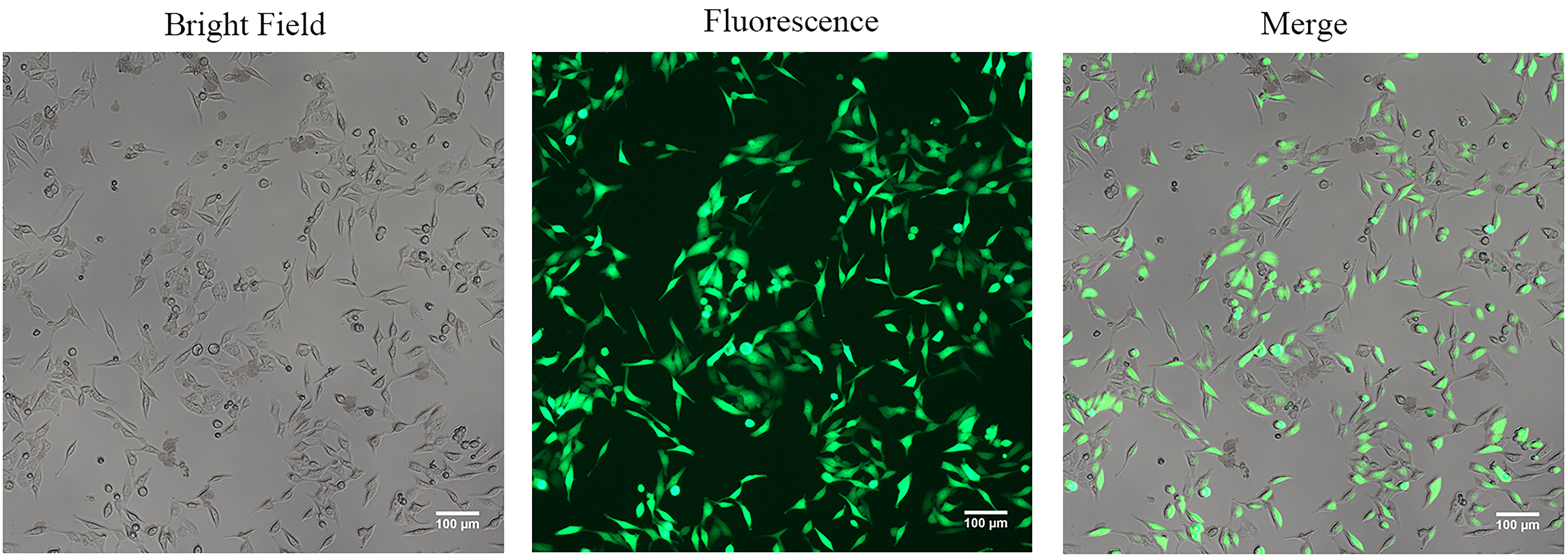

Supplement: Supplementary file 88 — Supplemental figure S1 [file 41420_2022_1021_MOESM88_ESM.tif]

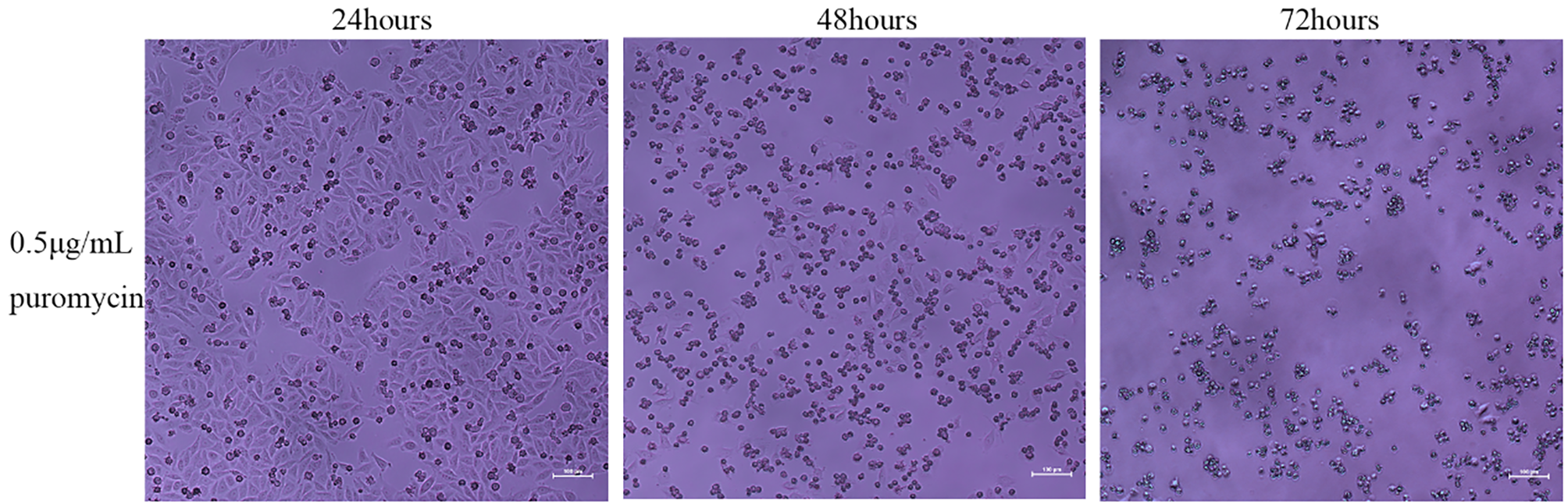

Supplement: Supplementary file 89 — Supplemental figure S2 [file 41420_2022_1021_MOESM89_ESM.tif]

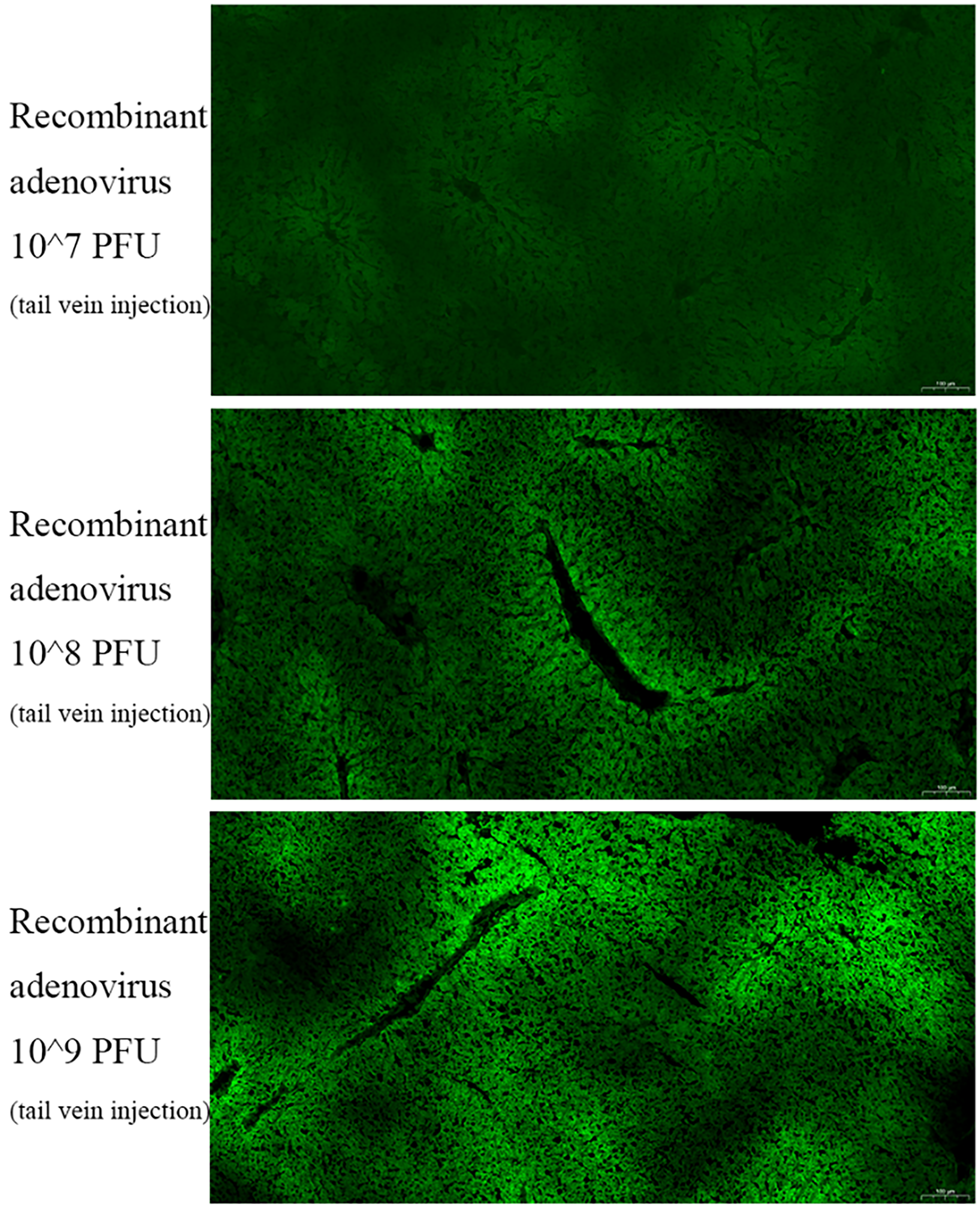

Supplement: Supplementary file 90 — Supplemental figure S3 [file 41420_2022_1021_MOESM90_ESM.tif]
