# Supplementary material for: PINK1 ameliorates acute-on-chronic liver failure by inhibiting apoptosis through mTORC2/AKT signaling
Source: Cell Death Discov. 2022 Apr 23;8:222. doi: 10.1038/s41420-022-01021-5 (PMC9035184; doi:10.1038/s41420-022-01021-5)
Supplement: Supplementary file 83 — Supplementary Table S1 [file 41420_2022_1021_MOESM83_ESM.doc]

**Supplementary Table S1** The baseline clinical data

| ID   | Name | Gender | Age | Etiology | ALT(U/L) | Donor's  ALT(U/L) |  | | --- | --- | --- | --- | --- | --- | --- | |
| --- | --- | --- | --- | --- | --- | --- | --- |
| 338973 Lvbo Yan male 48 HBV 63 43  341467 Maguang Shen male 36 HBV 219 47  341752 Xu Jun male 68 Alcoholic 14 8  325538 Tiegang Dong male 44 HBV 12 23  326745 Dianzhao Gao male 35 24 48  331242 Changlong Liu male 53 HBV 201 48  331852 Yaoxiong Zhang male 48 HBV 42 24  332745 Xuejun Zhang female 49 Alcoholic 16 41  332980 Jiangang Miao male 45 HBV 134 35  337472 Gongshuai Yu male 31 HBV 27 10  337788 Deming Liu male 38 HBV 32 36  142393 Guocang Zhang male 49 HBV 32 80  338498 Ruifeng He male 23 28  318667 Kangyu Gu male 35 HBV 244 41  318840 Guifen Song female 59 HBV 47 40  322994 Jianmin Li male 48 HBV 92  324301 Liu Fei male 40 HBV 386 64 |

| | AST(U/L) | Donor's  AST(U/L) | Tbil（umol/L） | Donor's PT(s) Donor's  Tbil（umol/L） PT(s) | | --- | --- | --- | --- | |
| --- | --- | --- | --- | --- |
| | 40 | 13 | 157.4 | 20.7 25.8 14.1 | | --- | --- | --- | --- | | 198 | 38 | 543.4 | 8.1 53.6 11.9 | | 75 | 11 | 335.8 | 18.2 41.7 12 | | 28 | 17 | 235.5 | 27.6 43.1 12.7 | | 70 | 26 | 230.2 | 54 29.2 11.8 | | 118 | 31 | 415.6 | 19.7 36.7 13.9 | | 57 | 11 | 149 | 22 21.6 15 | | 44 | 27 | 637.2 | 33.8 34.1 12.4 | | 175 | 19 | 339.7 | 19.1 28.4 12.3 | | 72 | 15 | 852.9 | 58.7 34.6 12.2 | | 64 | 21 | 517 | 27.1 21.3 12 | | 111 | 109 | 518.3 | 477.9 34.7 16.2 | | 66 |  | 405.2 | 84.7 | | 85 | 17 | 473.3 | 15 46.2 12.9 | | 70 | 22 | 157.6 | 40.2 25.5 11 | | 203 |  | 591.8 | 49.7 | | 511 | 29 | 337.1 | 15.1 73.5 13.3 | |

| | PTa(%) | Donor's PTa(%) | INR | Donor's INR | | --- | --- | --- | --- | |
| --- | --- | --- | --- | --- |
| | 33 | 72 | 2.27 | 1.26 | | --- | --- | --- | --- | | 14 | 91 | 4.64 | 1.06 | | 19 | 89 | 3.63 | 1.07 | | 17 | 81 | 3.7 | 1.13 | | 29 | 92 | 2.61 | 1.05 | | 22 | 73 | 3.28 | 1.24 | | 41 | 65 | 1.93 | 1.33 | | 24 | 86 | 2.98 | 1.11 | | 29 | 87 | 2.49 | 1.1 | | 24 | 88 | 3.09 | 1.09 | | 42 | 90 | 1.9 | 1.07 | | 23 | 59 | 3.03 | 1.44 | | 9 |  | 7.26 |  | | 16 | 81 | 4.13 | 1.15 | | 32 | 103 | 2.22 | 0.98 | | 15 |  | 4.44 |  | | 10 | 77 | 6.56 | 1.19 | |
