# Supplementary material for: PINK1 ameliorates acute-on-chronic liver failure by inhibiting apoptosis through mTORC2/AKT signaling
Source: Cell Death Discov. 2022 Apr 23;8:222. doi: 10.1038/s41420-022-01021-5 (PMC9035184; doi:10.1038/s41420-022-01021-5)
Supplement: Supplementary file 84 — Supplemental Materials and Methods [file 41420_2022_1021_MOESM84_ESM.doc]

**1. Chemicals, and** **Antibodies**

H2O2 and DAPI were purchased from Sigma. Akt (Akt inhibitor VIII or AKTi-1/2) and mTORC2 (JR-AB2-011) inhibitors, and AKT activator (SC79) were purchased from Merck. PE Annexin V Apoptosis Detection Kit I was purchased from BD Bioscience. The TUNEL kit was supplied by KeyGEN Biotech (KGA7073, Nanjing, China). The CCK8 kit was purchased from Abmole China Branch. The CCl_4_ was purchased from Macklin. The antibodies used were rabbit polyclonal anti-PINK1 (Novus Biologicals, 1:1000, BC100-494), anti-PINK1 (Cell Signaling Technology, [CST, USA], 1:1000, #6946), anti-Rictor (1:1000, #2114), anti-Rictor (CST, 1:20, #5379), anti-phospho-Ser-473 Akt (CST, 1:1000, #4060), anti-GAPDH (CST, 1:1000, #2118) and anti-cleaved caspase 3 (CST, 1:1000, #9661) primary antibodies, and HRP-labeled anti-rabbit secondary antibodies (CST, 1:1000, #7074).

**2. In vitro validation: cell experiments**

The treated cells were subjected to the following experiments: Group I (Control), L02 cells incubated at 37°C for 72 hours; Group II (Ad-Control or shControl; no-load group), L02 cells incubated with unloaded virus at a multiplicity of infection (MOI) of 100 PFU per cell at 37°C for 72 hours; Group III (H2O2-induced model), L02 cells incubated with H2O2 (0.4 mM) at 37°C for 24 hours; Group IV (H2O2+Ad-PINK1 or H2O2+shPINK1), L02 cells incubated with Ad-PINK1 or shPINK1 at a multiplicity of infection (MOI) of 100 PFU per cell at 37°C for 72 hours and H2O2 (0.4 mM) for 24 hours, as well as H2O2+Lenti-Rictor or H2O2+shRictor, Lenti-Rictor or shRictor (MOI=60 PFU) stable cells incubated with H2O2 (0.4 mM) at 37°C for 24 hours; and Group Ⅴ(H202+Ad-PINK1+shRictor), shRictor (MOI=60 PFU) stable cells incubated with Ad-PINK1 at an MOI of 100 PFU per cell at 37°C for 72hours, H2O2 (0.4 mM) for 24 hours, as well as H202+shPINK1+Lenti-Rictor, Lenti-Rictor (MOI=60 PFU) stable cells incubated with shPINK1 at an MOI of 100 PFU per cell at 37°C for 72 hours, H2O2 (0.4 mM) for 24 hours; H202+Ad-PINK1+AKT inhibitor Ⅷ, L02 cells incubated with Ad-PINK1 at an MOI of 100 PFU per cell at 37°C for 72 hours, AKT inhibitor Ⅷ (10µM) [26] for 12 hours and H2O2 (0.4 mM) for 24 hours; H202+Lenti-Rictor+AKT inhibitor Ⅷ (10µM), Lenti-Rictor (MOI=60 PFU) stable cells incubated with AKT inhibitor Ⅷ for 12 hours and H2O2 (0.4 mM) for 24 hours; H202+shRictor+AKT activator, shRictor (MOI=60 PFU) stable cells incubated with AKT activator SC79 (4µg/ml)[27] for 12 hours and H2O2 (0.4 mM) for 24 hours.

The cell line was authenticated and regularly tested for mycoplasma.

**3.Animal model of ACLF**

Animals were randomly divided into five groups (n = 5). In Group I (Normal), the rats received normal saline (6.6 mL/Kg, twice per week) for 8 weeks, with subsequent injection of normal saline (6.6 mL/kg, thrice a week) for 4 weeks. In Group II (ACLF model), the animals were intraperitoneally (i.p.) injected with vegetable oil and 20% CCl_4_ mixture (6.6 mL/kg), twice per week for 8 weeks, with subsequent injection of 20% CCl_4_ (6.6 mL/kg, thrice a week) for 4 weeks, followed by i.p. injection of LPS (10µg/kg)+D-Gal (500 mg/kg). In Group III (ACLF+Ad-Control or ACLF+shControl), the animals were administered vegetable oil and 20% CCl_4_ mixture (6.6 mL/kg), twice per week for 8 weeks, with subsequent injection of 20% CCl_4_ (6.6 mL/kg, thrice a week) for 4 weeks and no-load control viruses (5×10^8^ PFU) at 200µL per mouse by tail vein injection, once a week for 2 weeks at the beginning of the 10th week of CCl_4_ injection, followed by i.p. injection with LPS (10µg/kg)+D-Gal (500 mg/kg) at the beginning of the 12^th^ week of CCl_4_ injection. In Group Ⅳ (ACLF+Ad-PINK1 or ACLF+shPINK1), the animals received vegetable oil and 20% CCl_4_ mixture (6.6 mL/kg), twice per week for 8 weeks, with subsequent injection of 20% CCl_4_ (6.6 mL/kg, thrice a week) for 4 weeks, and Ad-PINK1 (2.5×10^8^ PFU) or shPINK1 (5.0×10^8^ PFU) [28], 200µL per mouse, once a week for 2 weeks at the beginning of the 10^th^ week of CCl_4_ injection, followed by i.p. injection with LPS (10µg/kg)+D-Gal (500 mg/kg) at the beginning of the 12^th^ week of CCl_4_ injection. In Group Ⅴ (ACLF+Ad-PINK1+Rictor inhibitor or ACLF+Ad-PINK1+AKT inhibitor Ⅷ), vegetable oil and 20% CCl_4_ mixture (6.6 mL/kg) was administered twice per week for 8 weeks, with subsequent injection of 20% CCl_4_ (6.6 mL/kg, thrice a week) for 4 weeks, Ad-PINK1 (2.5×10^8^ PFU) or shPINK1 (5.0×10^8^ PFU) and Rictor inhibitor JR-AB2-011 (20 mg/kg) [29] or AKT inhibitor Ⅷ(50 mg/kg) [30] by intraperitoneal (i.p.) injection, 200µL per mouse, once a week for 2 weeks at the beginning of the 10^th^ week of CCl_4_ injection, followed by i.p. injection with LPS (10µg/kg)+D-Gal (500 mg/kg) at the beginning of the 12^th^ week of CCl_4_ injection.

The method of randomization: Seven groups were set up. To ensure that all mice participating in the test are equally likely to be allocated to the seven groups, and make some features and interference factors that may affect the test results evenly distributed among the groups to make the groups comparable, the random number table method was adopted to allocate the mice, specifically: mice were numbered 01-35 in turn, then with the help of SAS 9.4 statistical software PROC PLAN process statement, the random number table is generated, and 35 number cards are set in sequence: 01, 02, 03,..., 34, 35. According to this allocation method, the 35 number cards are allocated to 7 groups according to the random number table, and finally the groups planned according to the mouse number is allocated to the corresponding groups. According to this method, each mouse was allocated in these 7 groups.

Table 1. The randomization number

| Group | Randomisation number | Group | Randomisation number | Group | Randomisation number |
| --- | --- | --- | --- | --- | --- |
| 1 | 3 | 3 | 1 | 5 | 11 |
| 1 | 6 | 3 | 2 | 5 | 13 |
| 1 | 9 | 3 | 28 | 5 | 20 |
| 1 | 19 | 3 | 29 | 5 | 25 |
| 1 | 32 | 3 | 31 | 5 | 27 |
| 2 | 7 | 4 | 4 | 6 | 5 |
| 2 | 15 | 4 | 10 | 6 | 8 |
| 2 | 16 | 4 | 14 | 6 | 23 |
| 2 | 17 | 4 | 18 | 6 | 24 |
| 2 | 30 | 4 | 34 | 6 | 33 |
| 7 | 12 | 7 | 22 | 7 | 35 |
| 7 | 21 | 7 | 26 |  |  |

The data collectors, outcome assessors, and the data analysts were blinded throughout the intervention process and until the end of the data analysis.

**4. CCK8 assay**

For the CCK8 assay, it was used according to the manufacturers instructions. Exponentially growing cells (5×10^4^/well) were seeded into 96-well plates, which were then incubated at 37°C with 5% CO2 for 24 h to 72 h. Subsequently, 10μl of CCK-8 solution (Abmole, USA) was added to 90μl culture medium. After 1-4 h at 37°C, absorbance was measured at a wavelength of 450 nm on a Thermo Scientific Multiskan GO spectrophotometer (ThermoFisher Scientific). The experiments were performed in triplicate, 3-6 times independently.

**5. TUNEL assay**

The experiment was operated according to kit instruction. L02 cells (5×10^4^) were seeded into 12-well plates and co-incubated with different drugs. After fixation with 4% paraformaldehyde for 10 min at room temperature, the cells were permeabilized with 1% Triton X-100 for 5 min; each sample was covered with 50 mL of TdT enzyme reaction solution and incubated for 1 h at 37ºC in a humidified box. After washing with fresh PBS, the cells were covered with 50 mL of streptavidin-fluorescein labeling buffer for 30 min at 37ºC. Finally, 10µl DAPI solution was added to label nuclei for 5 min. Apoptotic cells labeled with green fluorescence were observed by confocal laser scanning microscopy (CLSM, Leica, Germany).

Apoptosis in liver tissue samples was assessed following the manufacturer's protocol. Briefly, the samples were deparaffinized and rehydrated, followed by two incubations with xylene for 15-20 min. Dehydration was performed with pure ethanol for 10 min twice, followed by dehydration with a gradient ethanol of 95%, 90%, 80%, and 70% ethanol, respectively, for 5 min each. For antigen retrieval, proteinase K working solution was added to cover the slides, followed by incubation at 37℃ for 25 min. Permeabilization was performed by addition of permeabilization working solution to cover the tissue, followed by incubation at room temperature for 20 min. DAPI counterstaining was performed, and the coverslip was treated with anti-fade mounting medium. Finally, the sections were observed under a fluorescence microscope and images were collected.

**6. Western blot and coimmunoprecipitation (Co-IP) analyses**

Western blot: Briefly, mouse liver tissue (approximately 30 mg) or cells were incubated with RIPA lysis buffer (R0010, Solarbio, China). Protein concentrations were measured with the bicinchoninic acid (BCA) Protein Assay kit (PA101-01, Biomed, China). Proteins were separated by SDS-PAGE gel (3540124, Bio-Rad) and then electrophoretically transferred onto PVDF membranes (1620177, Bio-Rad). The membranes were incubated with primary antibodies overnight at 4°C and further incubated with HRP-conjugated secondary antibodies for 1 hour at 37°C. Protein bands were visualized using the ChemiDcoTM XRS ^+^ Imager-Bio-Rad imaging tool (Bio-Rad). The average intensities of various standard protein bands were quantified with ImageJ, and these results were normalized with GAPDH. GraphPad Prism 8.0 was used to generate curves.

Coimmunoprecipitation (Co-IP): The cell samples were incubated with IP lysis buffer (Servicebio, #G2038-100ML). Next, equal amounts of protein were incubated with 1.0μg rabbit IgG (1:100, Servicebio) as a polyclonal-isotype control, 10μL rabbit anti-PINK (1:50, CST), 10μL rabbit anti-Rictor (1:20, CST) or 10μL rabbit anti-p-AKT (1:50, CST) for 4 h at 4°C. Subsequently, the mixture (20μL) was incubated with 30μL protein A+G agarose beads (Millipore, IP05) at 4°C overnight. The mixtures were centrifuged (2000 g, 5 min, 4°C), and the beads were rinsed 4 times with phosphate-buffered saline (PBS). Next, immunocomplexes were boiled with 1×Western blot loading buffer. Total protein was extracted and boiled for 10 min at 100℃. Western blot was performed with the antibodies mentioned above.
