# Supplementary material for: PINK1 ameliorates acute-on-chronic liver failure by inhibiting apoptosis through mTORC2/AKT signaling
Source: Cell Death Discov. 2022 Apr 23;8:222. doi: 10.1038/s41420-022-01021-5 (PMC9035184; doi:10.1038/s41420-022-01021-5)
Supplement: Supplementary file 85 — Supplemental Result [file 41420_2022_1021_MOESM85_ESM.doc]

**H2O2-induced cell model**

To verify whether the H2O2-induced model conforms to the characteristics of ACLF, we examined the related proteins by Western blotting. We found that the expression levels of PINK1, mTORC2/Rictor, p-AKT and cleaved caspase3 in the 0.4 mM H2O2-induced cell model were consistent with the characteristics of ACLF (Supplemental Figure 1). Therefore, 0.4 mM H2O2 stimulation in L02 cells could well meet the ACLF cell protein expression, representing an ACLF cell simulation model, for the follow-up study of molecular mechanisms.
