# Supplementary material for: PINK1 ameliorates acute-on-chronic liver failure by inhibiting apoptosis through mTORC2/AKT signaling
Source: Cell Death Discov. 2022 Apr 23;8:222. doi: 10.1038/s41420-022-01021-5 (PMC9035184; doi:10.1038/s41420-022-01021-5)
Supplement: Supplementary file 86 — SUPPLEMENTAL LEGENDS [file 41420_2022_1021_MOESM86_ESM.doc]

**Supplemental legends**

**Supplemental figure 1 Effect of different concentrations of H2O2 on target proteins**

Different concentrations of H2O2 (0, 0.2, 0.3, 0.4, 0.6 and 0.8mM) were used to treat L02 cells in vitro. Protein expression levels of PINK1, mTORC2/Rictor, and p-AKT in H2O2-induced L02 cells were determined by Western blotting and quantitated with the Image J software. *, p<0.05; **, p<0.01; ***, p<0.001; ****, p<0.0001; ns, not significant.

**Supplemental figure S1** Fluorescence microscopy was also used to observe the transfected cells (scale bar, 100µm)

**Supplemental figure S2** After adding 0.5 µg/ml puromycin for 48 hours, widespread cell death was found in L02 cell lines, and almost complete cell death was seen after 72 hours (scale bar, 100µm)

**Supplemental figure S3** Ice-frozen tissues sections were examined by fluorescent microscopy to determine the amount of infectious virus (scale bar, 100 µm)
